# Sex Differences in Acute Coronary Syndromes: A Scoping Review Across the Care Continuum

**DOI:** 10.5334/gh.1410

**Published:** 2025-03-11

**Authors:** Anna Marzà-Florensa, Pauline Kiss, Dina Mohamed Youssef, Sara Jalali-Farahani, Fernando Lanas, Mariachiara di Cesare, José Ramón González Juanatey, Sean Taylor, Alicia Uijl, Diederick E. Grobbee, Sarah Des Rosiers, Pablo Perel, Sanne A. E. Peters

**Affiliations:** 1 Global Public Health and Bioethics, Julius Center for Health Sciences and Primary Care, University Medical Center Utrecht, Utrecht University, Utrecht, The Netherlands; 2 Institute of Public Health & Wellbeing, University of Essex, United Kingdom; 3 Universidad de La Frontera, Chile; 4 University Hospital, Santiago de Compostela, Spain; 5 World Heart Federation, Geneva, Switzerland; 6 Department of Cardiology, Amsterdam University Medical Centers, Amsterdam Cardiovascular Sciences, University of Amsterdam, Amsterdam, The Netherlands; 7 Division of Cardiology, Department of Medicine, Karolinska Institutet, Stockholm, Sweden; 8 The Novartis Foundation, Basel, Switzerland; 9 Department of Non Communicable Disease Epidemiology, London School of Hygiene & Tropical Medicine, United Kingdom; 10 The George Institute for Global Health, School of Public Health, Imperial College London, London, United Kingdom; 11 The George Institute for Global Health, University of New South Wales, Sydney, New South Wales, Australia

**Keywords:** acute coronary syndrome, sex differences, global health, cardiovascular disease, pre-hospital care, diagnosis

## Abstract

**Introduction:**

Optimal diagnosis and management of acute coronary syndrome (ACS) is essential to improve clinical outcomes and prognosis. Sex disparities in ACS care have been reported in the literature, but evidence gaps remain. This review aims to map and to summarize the global evidence on sex differences in the provision of care across the ACS continuum.

**Methods:**

A systematic literature search was conducted in Pubmed, EMBASE, and the World Health Organization Global Index Medicus. The search was restricted to original research articles published between January 1, 2013, and August 30^th^, 2023, and with a full-text available in English, Spanish, Dutch, or French. The search terms and key words covered five aspects of the ACS care continuum: pre-hospital care, diagnosis, treatment, in-hospital events, and discharge.

**Results:**

Of the 15,033 identified articles, 446 articles (median percentage of women per study: 29%), reporting on 1,483 outcomes, were included. Most studies were conducted in high-income regions (65%). Studies reported on pre-hospital care (8%), diagnosis (9%), treatment (45%), discharge (14%) and events (24%). For 45% of outcomes, results favored men, 5% favored women, and 50% showed mixed results or no sex difference. ACS care aspects with the largest sex differences were pre-hospital care (58% of the outcomes favored men vs 7% favored women) and diagnosis (70% favored men vs 2% favored women).

**Conclusion:**

Studies on sex differences in ACS mainly come from high-income regions. Sex differences in ACS management are widely reported and mainly unfavorable to women, especially in the early phases of pre-hospital care and diagnosis.

## Introduction

Ischemic heart disease (IHD) is the leading cause of death and disability in men and women globally (estimated 16% of total deaths in 2019), and most of the burden of IHD is caused by acute coronary syndrome (ACS) (1). ACS is characterized by a sudden block of blood supply to the heart muscle and encompasses a spectrum of conditions that include patients presenting with recent changes in clinical symptoms or signs; it can occur with changes on 12-lead electrocardiogram (ECG) or with acute elevations in cardiac troponin concentrations. Patients presenting with suspected ACS may eventually receive a diagnosis of acute myocardial infarction (AMI) or unstable angina (UA) (2).

There are sex differences in the presentation, management, and outcomes of ACS. ACS occurs at an older age in women than in men, and women present more often with additional symptoms besides chest pain (3). Also, despite higher mortality rates, women are less likely to receive guideline-recommended medications (4,5,6,7,8).

Previous reviews have compiled the available evidence on sex differences in diverse aspects of ACS, including risk factors (8), symptom presentation (3), management (4,8), outcomes (4,5), and care disparities in different regions (9). There are few reports of sex disparities in ACS outcomes from low- and middle-income countries (9), which face the highest burden of ACS (1). Indeed, age-standardised cardiovascular disease (CVD) death rates are the highest in Central/Eastern Europe, Central Asia and the North-Africa and Middle East region (10). Although existing reviews have investigated specific topics of ACS care, there is a lack of comprehensive analyses that consider the entire care continuum of ACS and include diverse geographic and economic contexts. Yet, to drive improvements in the care for ACS globally, it is essential to have a contextualized understanding of sex disparities in ACS.

This study therefore aimed to provide a comprehensive overview of the available literature on sex differences across the care continuum for ACS in multiple regions. With this goal, studies reporting sex differences in ACS provision of care globally, from pre-hospital care to hospital discharge, were reviewed and mapped.

## Methods

This study was pre-registered in Prospero (registration number CRD42023446481) and conducted in accordance with the PRISMA guidelines for scoping reviews (11) (**Supplementary Material 1**).

### ACS care continuum

A framework was developed to review five phases of the ACS care continuum: pre-hospital care, diagnosis, treatment, in-hospital events, and discharge (Table 1).

**Table 1:** ACS care continuum framework.


PHASE	TOPICS	SPECIFIC OUTCOMES REPORTED

**Pre-hospital**	Contact with health services	Contacting medial services, emergency medical services activation.

	Time to medical attention	Symptom onset to first medical contact, emergency services arrival time, scene time, ambulance time, scene to hospital time, symptom onset to presentation, pre-hospital delays.

	Diagnosis	Assessment for ACS, tests ordered, pre-hospital ECG.

	Treatment	Preadmission treatment, sending mobile ICU, Receiving protocol for MI, pre-hospital thrombolysis, referral to specialist, referral to hospital, use of medication (antithrombotics, analgesia, nitrates).

	Events	Cardiogenic shock, cardiac arrest, mortality.

	Other	Emergency medical services transport, use of pre-notification.

**Diagnosis**	Time	Time from door to ECG, time to angiography.

	Biomarkers	Use of biomarker testing, troponin testing.

	ECG	Use of ECG.

	Angiography	Use of angiography, radial access.

	Echocardiography	Use of echocardiography.

	Imaging	Imaging, coronary computed tomography, IVUS, OCT, FFR.

**Treatment**	Time	Symptom onset to angiography, time from admission to evaluation, ECG to angiography, time to treatment, first medical contact to PCI, Door to balloon time, time to CABG, in-hospital delay. Length of stay, mechanical support, CRP, circulatory support, extra corporeal membrane oxygenation, admission to hospital ward, CCU or ICU, transfer to cath lab.

	Revascularization	Receiving revascularization, invasive management, PCI, radial access, radial-to-femoral crossover, use of drug eluting stents, use of bare metal stents, plain balloon angioplasty, thrombus aspiration, CABG, medical management.

	Reperfusion (STEMI patients)	Reperfusion, thrombolysis, Fibrinolysis, PCI, stent implantation, drug-eluting stents, intra-aortic balloon-pump, aspiration thrombectomy, CABG, angioplasty.

	Antithrombotic medications	Medication use, evidence-based medications, guideline recommended medications, optimal medical management: antithrombotics, antiplatelet, aspirin, clopidogrel, prasugrel, ticagrelor, glycoprotein *IIb/IIIa* inhibitors, P2Y12 inhibitors, dual antiplatelet therapy, anticoagulants, heparin, fondaparinaux, warfarin, bivalirudin.

	Antihypertensive medications	Medication use, evidence-based medications, guideline recommended medications, optimal medical management: antihypertensives, beta-blockers, ACE-inhibitors, ARBs, calcium channel blockers, diuretics, vasodilators.

	Lipid-lowering medications	Medication use, evidence-based medications, guideline recommended medications, optimal medical management: lipid-lowering, statins, ezetimibe.

	Other medications	Medication use, evidence-based medications, guideline recommended medications, optimal medical management: anti-ischemic, nitrates, opioids, oral antidiabetics, insulin, traditional Chinese medicine.

**Events**	Complications	Cardiovascular complications, stroke, shock, bleeding, cardiac arrest, reinfarction, mitral regurgitation, atrioventricular block, ventricular fibrillation, ventricular tachycardia, arrythmia, pericardial effusion, cardiac tamponade, Mechanical complications, free wall rupture, papillary muscle rupture, ventricular septal rupture, coronary perforation or dissection, side branch occlusion, femoral pseudoaneurism, Respiratory failure, acute kidney injury, nephropathy, pneumonia, gastrointestinal bleeding, prolonged ventilation, CABG-related complications, transradial access failure, stent complications, major adverse cardiovascular and cerebrovascular events, net adverse cardiovascular events.

	Mortality	In-hospital mortality.

**Discharge**	Antithrombotic medications	Medication use, evidence-based medications, guideline recommended medications, optimal medical management: antithrombotics, antiplatelet, aspirin, clopidogrel, prasugrel, ticagrelor, glycoprotein *IIb/IIIa* inhibitors, P2Y12 inhibitors, dual antiplatelet therapy, anticoagulants, heparin, fondaparinaux, warfarin, bivalirudin.

	Antihypertensives medications	Medication use, evidence-based medications, guideline recommended medications, optimal medical management: antihypertensives, beta-blockers, ACE-inhibitors, ARBs, calcium channel blockers, diuretics, vasodilators.

	Lipid-lowering medications	Medication use, evidence-based medications, guideline recommended medications, optimal medical management: lipid-lowering, statins, ezetimibe.

	Other medications	Medication use, evidence-based medications, guideline recommended medications, optimal medical management: anti-ischemic, nitrates, opioids, oral antidiabetics, insulin, traditional Chinese medicine.

	Advice	Advice on diet and exercise, smoking cessation counseling.

	Cardiac rehabilitation	Referral and uptake of cardiac rehabilitation.

	Other	Discharge on the same day, discharge to skilled nurse facility.


ACS = acute coronary syndrome; ECG = electrocardiogram; ICU = intensive care unit; MI = myocardial infarction; IVUS = intravascular ultrasound; OCT = optical coherence tomography; FFR = fractional flow reserve; CABG = coronary artery bypass grafting; PCI = percutaneous coronary intervention; ACE-inhibitors = angiotensin-converting enzyme inhibitors; ARBs = angiotensin receptor blockers; CCU = cardiac care unit.

The pre-hospital phase includes the time to receive medical attention, use of pre-hospital diagnostic tests, and treatment strategies during the acute phase. The diagnostic phase includes the use of diagnostic tests such as biomarkers, electrocardiogram (ECG), angiography, and imaging techniques. The treatment phase includes in-hospital time to treatment, use of medications (i.e., antithrombotics, antihypertensives, lipid-lowering medication and other medications), revascularization, and, in STEMI patients, reperfusion. The events phase includes in-hospital complications and in-hospital mortality. The discharge phase includes the prescription of medications, lifestyle advice, and referral to cardiac rehabilitation.

#### Search and eligibility criteria

A search was conducted in Pubmed, EMBASE, and World Health Organization Global Index Medicus databases for each phase in the framework (**Supplementary Material 2**). Searches were made for studies with a study population described by one of the following diagnostic terms: ACS, MI (unspecified), heart attack/infarct, UA, STEMI or NSTEMI. The search was limited to original research articles published between January 1^st^ 2013 and August 30^th^ 2023 and with full text available in English, Spanish, Dutch or French. Studies were included in the review if they fulfilled the following criteria: they included patients with ACS, the abstract reported a statistical comparison of sex differences in at least one of the five phases as defined by the framework, and they included at least 1000 ACS patients.

#### Screening, data extraction and statistical analyses

Search results were transferred to Endnote and duplicates were removed. The software Rayyan CQRI (12) was used for title and abstract screening. Three independent reviewers (AMF, PK, SJF) screened the study titles and abstracts. Ten percent of the articles were screened in duplicate to reduce inter-reviewer variability and disagreements were discussed until a consensus was reached. Full text screening of selected articles was also done by the three reviewers.

Data extraction on study characteristics and results of included articles was done by three independent reviewers (AMF, PK, DMY), with 10% overlap. Countries were grouped into seven regions according to the Global Burden of Disease 2019 classification (1).

The extracted information was mapped by reporting the number of articles by topic, region, and sample size of the included studies. Results on reported sex differences were grouped into four categories, based on statistical tests: favors men, favors women, no sex differences, or mixed findings. When an outcome favored men (or women), it indicated shorter time in men (or women) (for example, shorter times to medical attention, shorter length of stay in the hospital, or a higher frequency of procedures or treatment provided). The category ‘mixed findings’ was used when findings on one outcome (e.g., antihypertensives) varied for different subcategories of that outcome. For example, a study could report higher prescription of angiotensin-receptor blocker in men and no sex differences for ACE-inhibitors. The category ‘no sex differences’ was used when no statistically significant sex difference was observed (p-value for significance >0.05). The following text provides a guide for interpreting the direction of sex differences in the various outcomes in the review, and **Supplementary Material 3** shows examples of outcomes favoring men, favoring women, mixed, and without differences, for the different phases and topics of ACS care continuum.

**Sex differences direction interpretation:**

– **Favors men:** indicates shorter time to medical attention, diagnostic tests, procedures or treatments in time-related outcomes; shorter length of stay; higher frequency of tests, procedures or treatments provided; or lower occurrence of events in men compared to women.
– **Favors women:** indicates shorter time to medical attention, diagnostic tests, procedures or treatments in time-related outcomes; shorter length of stay; higher frequency of tests, procedures or treatments provided; or lower occurrence of events in women compared to men.
– **Mixed:** indicates sex differences in different directions for sub-categories of an outcome that are not reported individually.
– **No difference:** indicates that sex differences reported for the outcome are not statistically significant (significance is defined at p < 0.05).

Descriptive analyses were used in this study, including summary statistics and frequency counts. A sensitivity analysis on the subset of studies that conducted statistically adjusted analyses was carried out.

## Results

**Figure 1:**
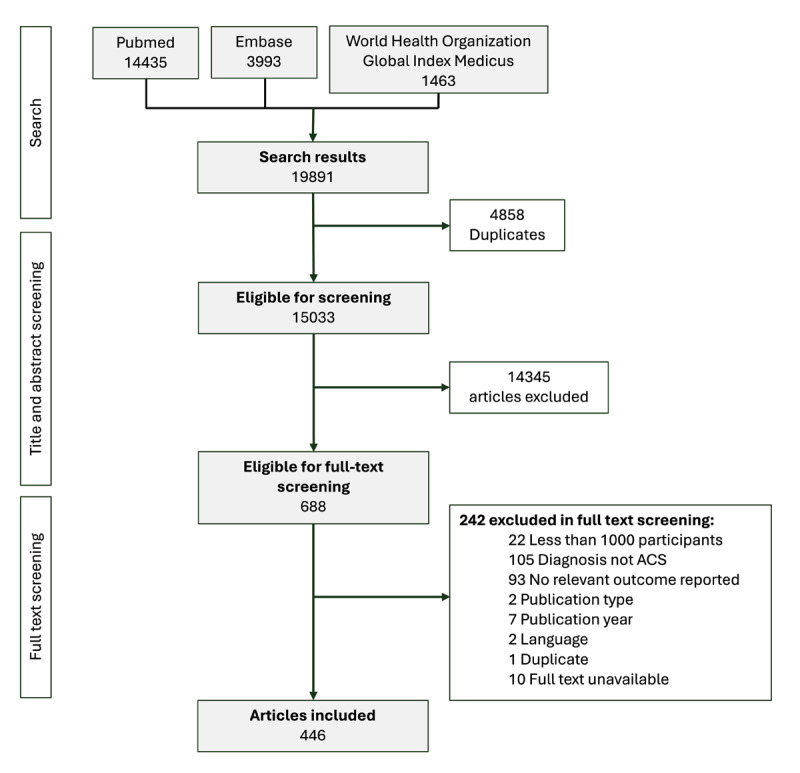
Flowchart of study selection.

The search yielded 15,033 unique articles, of which 688 were screened full-text. Of these, 446 articles met the eligibility criteria and were included (Figure 1, **Supplementary Materials 4–5**).

### Study characteristics

**Figure 2:**
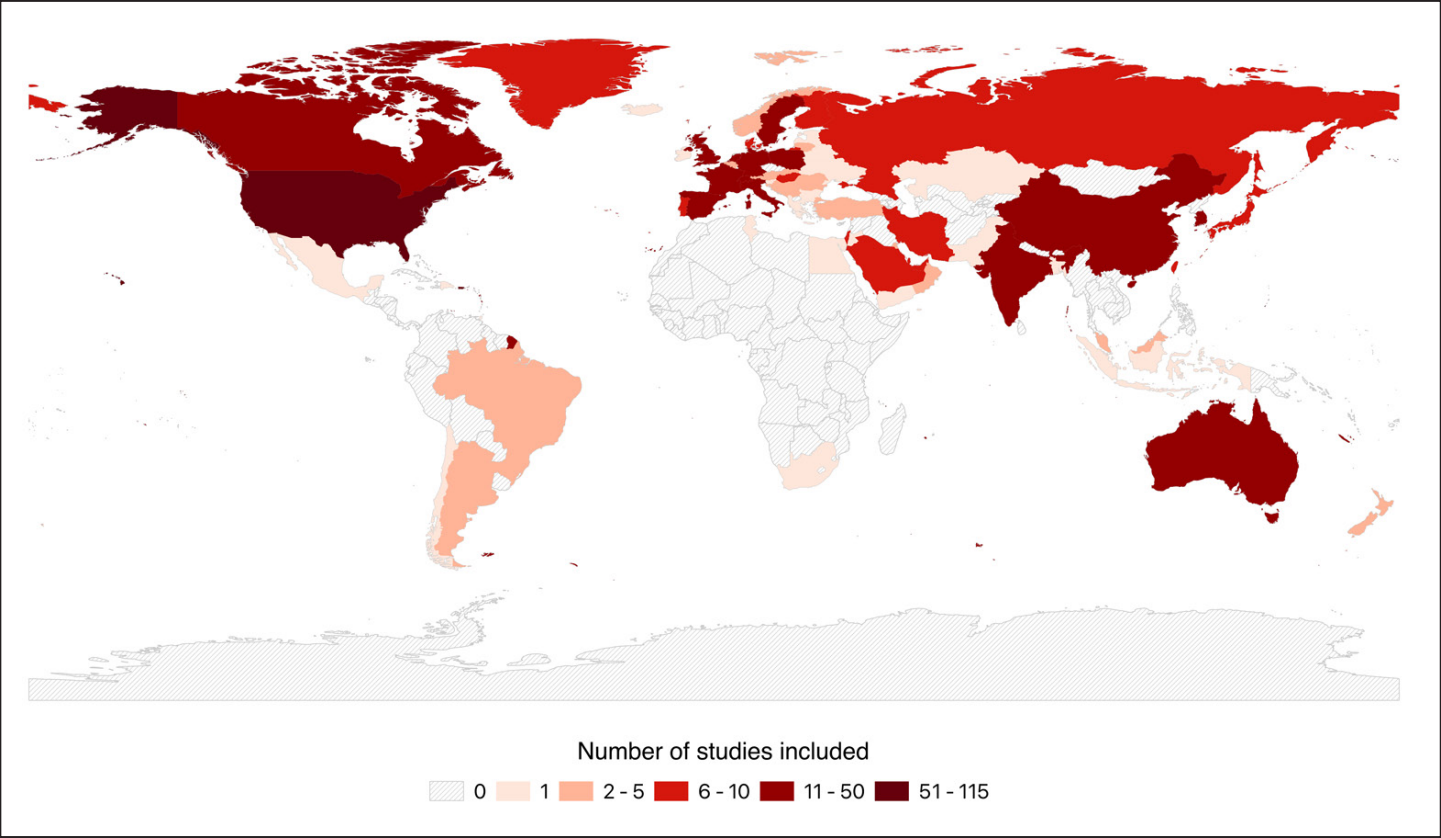
Map with number of publications by country.

Data on 32,875,226 study participants was reported (median per study: 7,597 [Interquartile range (IQR): 2,600–43,272]) derived out of 163 unique databases. The median percentage of women was 29% [IQR 24–36] and the study years ranged from 1972 to 2022. The included studies covered 73 countries: 65% from high-income regions (Figure 2); 9% from South-East Asia, East Asia, and Oceania; 6% from North Africa and Middle East, 6% from Central Europe, Eastern Europe and Central Asia; 3% from South Asia; and 2% from Latin America and the Caribbean. Nine percent of the studies were conducted in multiple regions.

Patients in the included studies had a diagnosis of ACS (33% of studies), myocardial infarction (MI) (unspecified) (31% of studies), acute ST-elevation myocardial infarction (STEMI) (28% of studies), or Non-STEMI (NSTEMI) (4% of studies). Four percent of studies included ACS patients undergoing a medical procedure (e.g., percutaneous coronary intervention (PCI), coronary artery bypass graft surgery (CABG)) or manifestation of ACS (e.g., cardiogenic shock, cardiac arrest).

Overall, studies reported on 1,483 outcomes, with 8% on pre-hospital care, 9% on diagnosis, 45% on treatment, 24% on events, and 14% on discharge.

#### Sex differences in ACS across the care continuum

**Figure 3:**
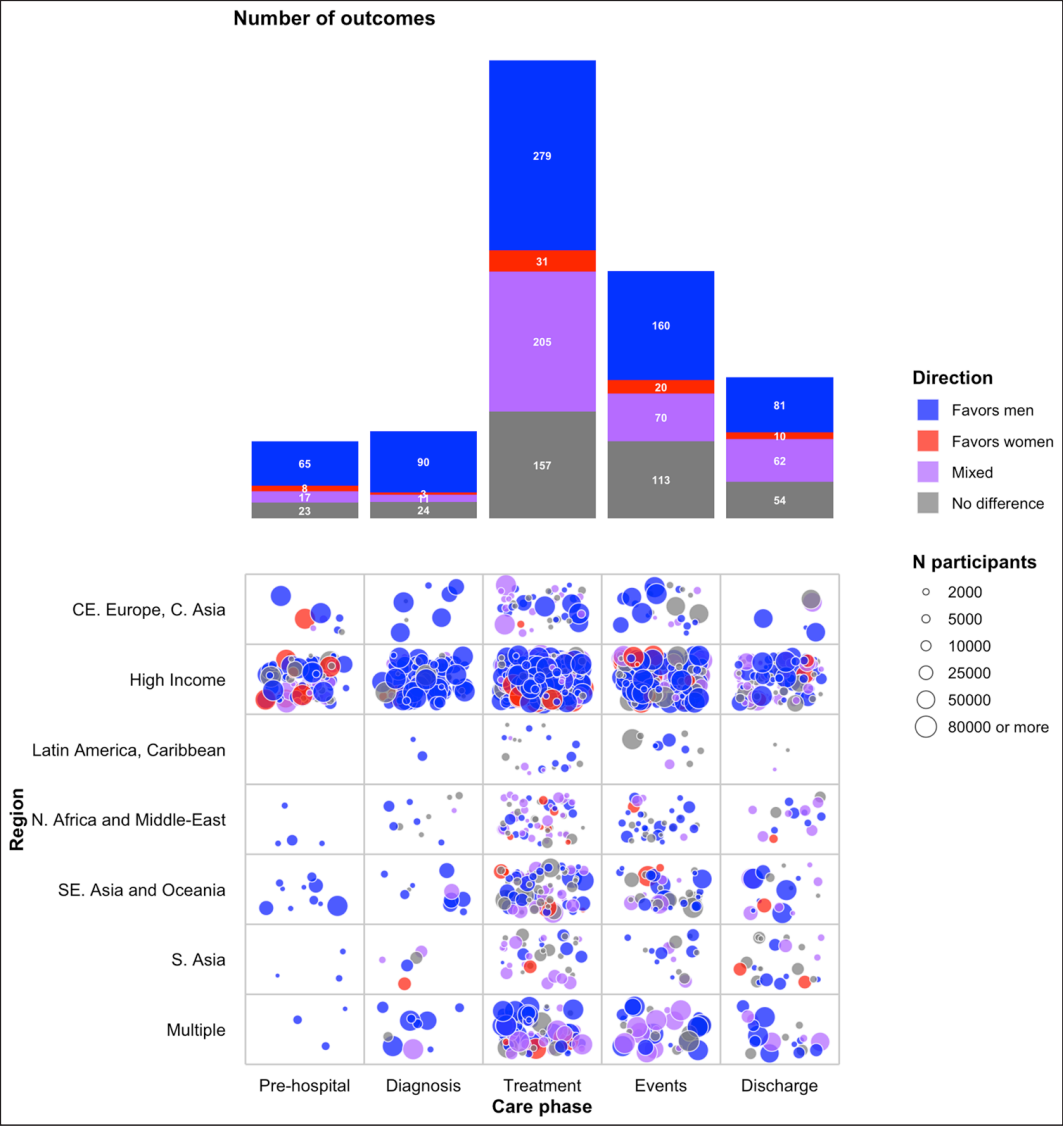
Mapping of the available evidence by region and care phase.

From all outcomes combined, 45% favored men, 5% favored women, 25% showed mixed results, and 25% showed no difference. Figure 3 shows the number of outcomes by care phase and their direction. The outcomes by care phase, region, and direction are shown in **Supplementary Material 6**.

##### Pre-hospital care

The 94 studies with 113 outcomes in the pre-hospital phase related to time to contact and/or medical attention (63%), diagnosis (12%), pre-hospital treatment (10%), pre-hospital events (10%), contacting health services (3%), and others (2%).

**Figure 4:**
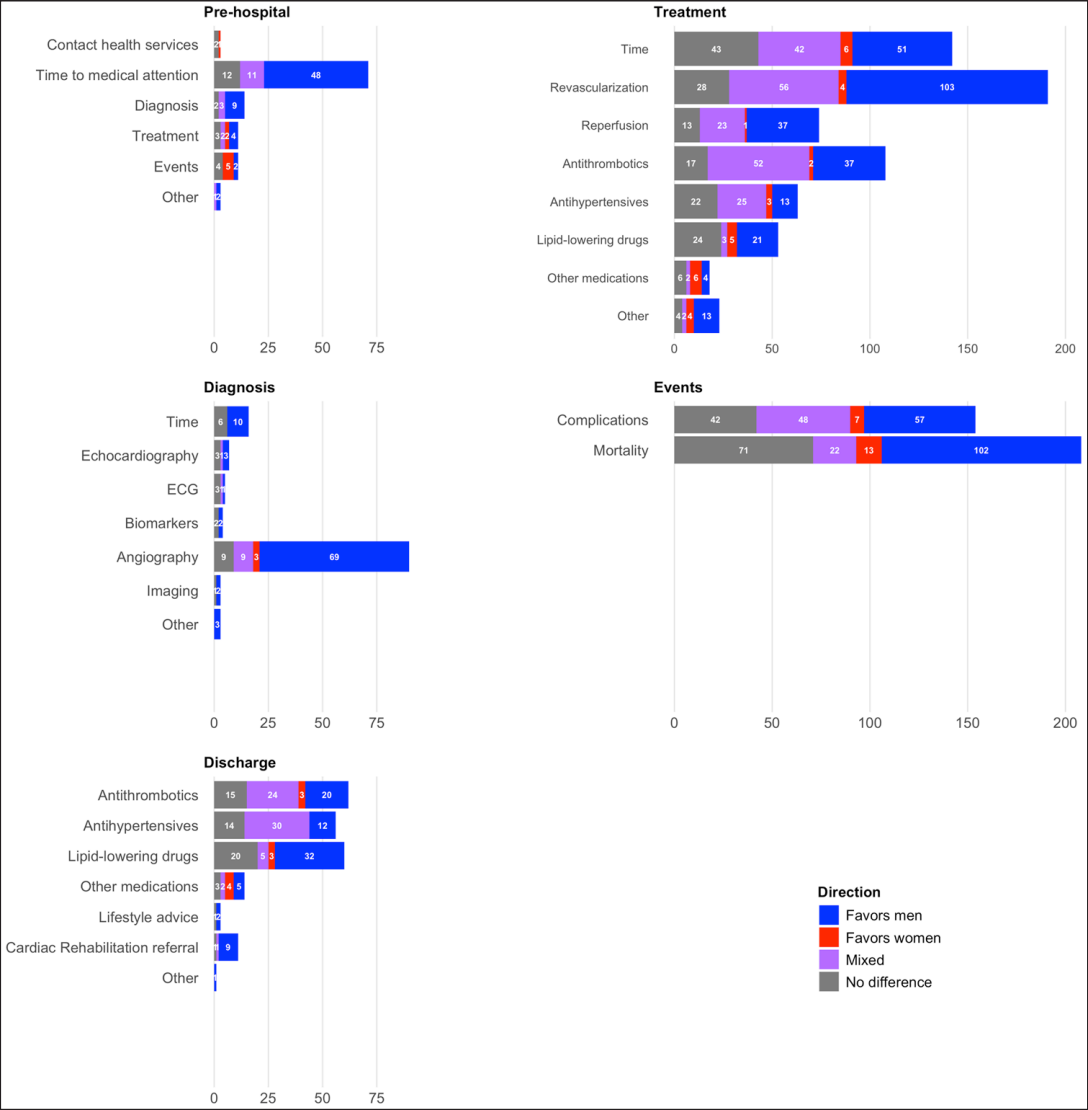
Number of outcomes and direction by care topic and phase.

Overall, 58% of the outcomes favored men, 7% favored women, 15% were mixed, and 20% showed no sex difference. For the time to contact and/or medical attention, 68% of the outcomes favored men, 15% were mixed (i.e., some outcomes favoring men and some favoring women), and 17% showed no difference (Figure 4).

##### Diagnosis

Of the 105 studies with 128 outcomes in the diagnostic phase, 70% reported on the use of angiography. Other outcomes were time to diagnosis (13%), use of echocardiography (6%), use of ECG (4%), biomarker tests (3%), imaging (2%), others (2%).

Seventy percent of outcomes favored men, 2% favored women, 9% were mixed, and 19% showed no sex difference. For angiography, 77% of the outcomes favored men, 3% favored women, and ten percent showed mixed outcomes or no difference (Figure 4).

##### Treatment

Of the 298 studies with 672 outcomes on treatment, 29% were on revascularization, 21% on time to receive treatment, and 11% on reperfusion among STEMI patients. Thirty-six percent of outcomes reported medication use, of which 45% were on antithrombotics, 26% on antihypertensives, 22% on lipid-lowering drugs, and 7% on other medications.

Overall, 41% of the outcomes favored men, 5% favored women, 31% were mixed, and 23% showed no sex difference. Fifty-four percent of the outcomes on revascularization showed higher use in men, 2% showed higher use in women, 29% showed mixed evidence, and 15% showed no sex difference. Similar results were seen for the use of reperfusion among STEMI patients. For pharmacological treatment: 34% of the outcomes on antithrombotics favored men, 2% favored women, 48% were mixed, and 16% showed no sex difference. For antihypertensives, 20% of the outcomes favored men, 5% favored women, 40% showed mixed results, and 35% showed no sex difference. For lipid-lowering medications, 40% of the outcomes showed that medications were more often given to men, 9% favored women, six percent were mixed, and 45% showed no sex difference (Figure 4).

##### Events

There were 255 studies covering 362 outcomes related to in-hospital events, with mortality reported in 57% of the outcomes and complications reported in 43%.

Forty-nine percent showed lower mortality in men, 6% showed lower mortality in women, 11% were mixed, and 34% of the outcomes showed no difference. Thirty-seven percent of the outcomes related to in-hospital complications showed lower occurrence of events in men, 5% showed lower occurrence in women, 31% were mixed, and 27% showed no sex difference (Figure 4).

##### Discharge

Of the 76 studies with 207 outcomes on hospital discharge, 93% reported on medications, 5% on cardiac rehabilitation, and 1% on advice.

Overall, 39% of outcomes favored men, five percent favored women, 30% of the outcomes showed mixed evidence, and 26% showed no sex difference. Thirty-two percent of the outcomes on antithrombotics favored men, 5% favored women, 39% were mixed, and 24% showed no difference. As for the antihypertensive medications, 21% of the outcomes favored men, 54% of the evidence was mixed, and 25% showed no difference. Fifty-three percent of the outcomes showed that lipid-lowering medications were prescribed more to men at discharge, while 5% favored women, nine percent were mixed, and 33% of the outcomes showed no sex difference (Figure 4).

#### Sensitivity analysis

In total, 41% of the outcomes were adjusted for possible confounders, with 76% of the studies (n = 337) reporting at least one adjusted outcome. All care phases had more unadjusted than adjusted outcomes with the exception of the events phase (70% of outcomes were adjusted) (**Supplementary Materials 7**).

Adjusted outcomes showed overall similar directions as the main analysis (i.e., favoring men), with lower percentages of mixed results and higher percentages of absence of sex differences.

## Discussion

This study provides a global overview of the evidence from 446 articles reporting sex differences in 1,483 outcomes in the provision of care across the ACS continuum. The study provides several important insights. First, the majority of data on sex differences in ACS comes from high-income regions. Second, most of the evidence focused on the treatment and event phases, while less than 20% of the studies reported on the pre-hospital and diagnostic phases. Third, major sex differences were found across the ACS care continuum, with 45% of findings favoring men, 5% favoring women, and 50% finding no or mixed sex differences. Finally, while only 40% of outcomes were adjusted for other important covariates that might explain the sex differences, the general patterns of ACS care favoring men persisted across adjusted outcomes.

This study showed a disparity in evidence distribution across the globe. While data from 73 different countries were included, only one study included Sub-Saharan Africa (South Africa), and only 2% of studies were conducted in the Latin America and Caribbean region. This review also showed sex differences in ACS across the care continuum, most commonly to the disadvantage of women. In the pre-hospital phase, we found longer pre-hospital times in women than in men. Higher patient awareness of ACS symptoms is a key factor in reducing time to treatment (13). Compared with men, women are more likely to underestimate their own risk of ACS (14). Health care providers may also underestimate the risk in women. Women may also be more likely to misinterpret ACS symptoms and attribute them to non-cardiac origin (15). Anxiety and unwillingness to trouble the family are other factors that could explain the greater delays in women (16). Timely pre-hospital diagnosis is another important target, as a study found that women have longer delay times to receiving pre-hospital ECG than men (17). Public campaigns, such as the HELP campaign by the Swiss Heart Foundation, might be an effective tool to increase symptom awareness in the general population (18,19,20). A recent study from Switzerland found that pre-hospital delays between the onset of heart attack symptoms and hospital admission have been steadily decreasing over the last two decades, and more greatly in women, leading to a reduction in the sex gap between 2002 and 2019. This gap even disappeared in 2019 after adjustment for patient characteristics (19).

Recent studies have debunked the misperception that the symptoms of ACS are different between women and men (9,21). Instead, while the main symptoms are the same and include chest pain, shortness of breath, and discomfort in the (left) arm, there are also additional symptoms, such as nausea, tiredness, and cold sweat, that are more common in women (3). Timely recognition and diagnosis of ACS is needed to avoid delays in treatment initiation and cardiac intervention (9). The findings highlight the underutilization of invasive cardiac evaluation in women. The reasons behind the sex gap in diagnostic coronary angiography remain elusive. A Canadian study showed that more women than men are not referred for diagnostic coronary angiography because their physician found that the benefit of the invasive strategy was not supported by evidence (22). Moreover, a study from Denmark investigating the use of diagnostic coronary angiography (DCA) in individuals with acute MI found that the decision not to refer to a DCA was clinically justified in more than 80% of the cases, for example, because the test was already performed, was declined by the patient, or because of high age. However, women were overrepresented in the remaining 20% among whom non-referral was not justified (23). It has also been suggested that women are less likely to accept recommendations for cardiac diagnostic procedures than men (24). This could stem from women’s underestimation of their risk and disease severity (24). The results of this study show that women are less likely to undergo cardiac revascularization than men. The GENESIS-PRAXY study found that anxiety, more risk factors, feminine traits (i.e., as defined by a high Bem feminity score (25)), and lack of chest pain at presentation were determinants of reduced access to care among young ACS patients (26).

For medications, the evidence was predominantly mixed for antithrombotics and antihypertensives. This observation can be explained by the heterogeneity in prescribing patterns found in the literature: women are more likely to receive dual platelet therapy with clopidogrel than prasugrel or ticagrelor, due to the increased bleeding risk (27). Likewise, women are more likely to be prescribed diuretics but less likely to be prescribed ACE-inhibitors, compared to men (28). The direction of the outcomes on lipid-lowering drugs more distinctly favored men. Despite evidence of similar effectiveness of statin therapy for both sexes (29), women are less likely to be prescribed statins than men. The findings also highlighted sex disparities in the referral for cardiac rehabilitation. Despite evidence of equal benefits for both sexes (30), women are less likely to be referred by their physician. This can be partly explained by doctor’s skepticism about the added value of these programs, as well as concerns that women are too elderly or have too many comorbidities to participate in cardiac rehabilitation (31).

The worse prognosis following ACS in women compared with men can be explained by the older age at admission, pre-hospital delays, and disparity in the provision of PCI (32,33,34,35). A French study looking at sex differences in hospital mortality of acute MI patients performed simulations of the expected mortality in women (33). They showed that sex disparities in the provision of reperfusion accounted for one quarter of the modifiable excess mortality (33). Women have a higher likelihood of experiencing major cardiovascular events and bleeding (35,36). Potential explanations include pre-operative characteristics, smaller artery size and the higher prevalence of microvascular dysfunction in women (35).

With increasing urbanization and population growth and aging, the burden of cardiovascular disease has shifted to Low and Middle Income Country (LMIC) settings, causing substantial pressure on already over-burdened health care systems (37,38). The CVD epidemic is associated with increasing socioeconomic costs, with a rise in disability and lowering of productivity that reinforce health inequalities (38). In addition, women are disproportionally affected by poverty and limited access to health care in these regions, and awareness about cardiovascular disease risk in women is low (14). While progress in cardiovascular health research is concentrated in high-income countries, about four in every five CVD deaths occur in LMICs (10). Substantive effort is needed to achieve an equitable distribution of prevention, diagnosis, and treatment of CVDs. Although this review has a global scope and shows widespread sex disparities, the drivers of these differences are specific to care phases and to regions. Future research investigating sex disparities in ACS care provision in localized contexts would therefore allow the identification of specific challenges, and support the development of improvements that are actionable and effective locally.

To our knowledge, this is the first review on sex differences in the care of patients with acute coronary syndromes, from pre-hospital care to hospital discharge. Strengths of this study include the broad and global focus on sex comparisons across the ACS care continuum. The quality of the review was optimized by implementing an ACS framework, selecting studies with larger sample sizes that included statistical comparisons, using contemporary data and by the collaboration of four independent reviewers. The consistency between the main results and the sensitivity analysis reinforces the robustness of the findings. Limitations of the study are associated with its wide focus and include the heterogeneity in study populations and study designs that precluded a meta-analysis as well as the inability to identify explanations for the sex differences found. Although, potential confounders, such as disease severity, were not studied, the results of a sensitivity analysis conducted with the publications that performed covariate adjustment were similar to the main analysis. Despite the large number of articles reviewed, evidence from some world regions was limited, affecting the generalizability of the results. A comprehensive search was conducted in multiple databases. However, the possibility that other relevant articles could have been identified by searching additional databases cannot be excluded. The search restriction to studies that statistically compared sex differences might have induced publication bias. Although measures were taken to prevent the inclusion of multiple articles reporting on the same patients, possible patient overlap cannot be completely ruled out. Another limitation was that a risk of bias assessment for the included studies was not conducted. However, this approach is in line with the scoping reviews methodology, which aims to map the breadth of evidence on a topic rather than assess the quality of individual studies.

In conclusion, this comprehensive review shows that literature on sex differences in ACS care comes mainly from high-income regions. Sex differences in ACS management are present across the full continuum of care and are largely unfavorable to women, especially in the early phases of pre-hospital and diagnosis. This study will contribute to raising awareness amongst policy makers and health authorities on the existing disparities. Efforts should focus on closing the evidence gaps and actions are needed to optimize ACS care in women and men.

## Additional File

The additional file for this article can be found as follows:

10.5334/gh.1410.s1 Supplementary files. Supplementary Material 1 to 7.
