# Supplementary material for: Sex Differences in Acute Coronary Syndromes: A Scoping Review Across the Care Continuum
Source: Glob Heart. 2025 Mar 11;20(1):26. doi: 10.5334/gh.1410 (PMC11908429; doi:10.5334/gh.1410)
Supplement: Supplementary files. — Supplementary Material 1 to 7. [file gh-20-1-1410-s1.pdf]

**Supplementary Material 1. Preferred Reporting Items for Systematic reviews and Meta-Analyses extension for Scoping Reviews (PRISMA-ScR) Checklist.**

| SECTION | ITEM | PRISMA-ScR CHECKLIST ITEM | REPORTED ON PAGE # |
|---|---|---|---|
| **TITLE** | | | |
| Title | 1 | Identify the report as a scoping review. | 1 |
| **ABSTRACT** | | | |
| Structured summary | 2 | Provide a structured summary that includes (as applicable): background, objectives, eligibility criteria, sources of evidence, charting methods, results, and conclusions that relate to the review questions and objectives. | 2 |
| **INTRODUCTION** | | | |
| Rationale | 3 | Describe the rationale for the review in the context of what is already known. Explain why the review questions/objectives lend themselves to a scoping review approach. | 3 |
| Objectives | 4 | Provide an explicit statement of the questions and objectives being addressed with reference to their key elements (e.g., population or participants, concepts, and context) or other relevant key elements used to conceptualize the review questions and/or objectives. | 3 |
| **METHODS** | | | |
| Protocol and registration | 5 | Indicate whether a review protocol exists; state if and where it can be accessed (e.g., a Web address); and if available, provide registration information, including the registration number. | 4 |
| Eligibility criteria | 6 | Specify characteristics of the sources of evidence used as eligibility criteria (e.g., years considered, language, and publication status), and provide a rationale. | 4 |
| Information sources* | 7 | Describe all information sources in the search (e.g., databases with dates of coverage and contact with authors to identify additional sources), as well as the date the most recent search was executed. | 4 |
| Search | 8 | Present the full electronic search strategy for at least 1 database, including any limits used, such that it could be repeated. | Suppl. Material 2 |
| Selection of sources of evidence† | 9 | State the process for selecting sources of evidence (i.e., screening and eligibility) included in the scoping review. | 4-5 |
| Data charting process‡ | 10 | Describe the methods of charting data from the included sources of evidence (e.g., calibrated forms or forms that have been tested by the team before their use, and whether data charting was done independently or in duplicate) and any processes for obtaining and confirming data from investigators. | 5 |
| Data items | 11 | List and define all variables for which data were sought and any assumptions and simplifications made. | 4-5-6, Table 1 |
| Critical appraisal of individual sources of evidence§ | 12 | If done, provide a rationale for conducting a critical appraisal of included sources of evidence; describe the methods used and how this information was used in any data synthesis (if appropriate). | / |
| Synthesis of results | 13 | Describe the methods of handling and summarizing the data that were charted. | 6 |
| **RESULTS** | | | |
| Selection of sources of evidence | 14 | Give numbers of sources of evidence screened, assessed for eligibility, and included in the review, with reasons for exclusions at each stage, ideally using a flow diagram. | 7, Figure 1 |
| Characteristics of sources of evidence | 15 | For each source of evidence, present characteristics for which data were charted and provide the citations. | Suppl. Material 4-5 |
| Critical appraisal within sources of evidence | 16 | If done, present data on critical appraisal of included sources of evidence (see item 12). | / |
| Results of individual sources of evidence | 17 | For each included source of evidence, present the relevant data that were charted that relate to the review questions and objectives. | / |

| SECTION | ITEM | PRISMA-ScR CHECKLIST ITEM | REPORTED ON PAGE # |
|---|---|---|---|
| Synthesis of results | 18 | Summarize and/or present the charting results as they relate to the review questions and objectives. | 7-10, Figures 2-4 |
| **DISCUSSION** | | | |
| Summary of evidence | 19 | Summarize the main results (including an overview of concepts, themes, and types of evidence available), link to the review questions and objectives, and consider the relevance to key groups. | 11 |
| Limitations | 20 | Discuss the limitations of the scoping review process. | 14 |
| Conclusions | 21 | Provide a general interpretation of the results with respect to the review questions and objectives, as well as potential implications and/or next steps. | 14 |
| **FUNDING** | | | |
| Funding | 22 | Describe sources of funding for the included sources of evidence, as well as sources of funding for the scoping review. Describe the role of the funders of the scoping review. | / |

JBI = Joanna Briggs Institute; PRISMA-ScR = Preferred Reporting Items for Systematic reviews and Meta-Analyses extension for Scoping Reviews.

* Where *sources of evidence* (see second footnote) are compiled from, such as bibliographic databases, social media platforms, and Web sites.

† A more inclusive/heterogeneous term used to account for the different types of evidence or data sources (e.g., quantitative and/or qualitative research, expert opinion, and policy documents) that may be eligible in a scoping review as opposed to only studies. This is not to be confused with *information sources* (see first footnote).

‡ The frameworks by Arksey and O'Malley (6) and Levac and colleagues (7) and the JBI guidance (4, 5) refer to the process of data extraction in a scoping review as data charting.

§ The process of systematically examining research evidence to assess its validity, results, and relevance before using it to inform a decision. This term is used for items 12 and 19 instead of "risk of bias" (which is more applicable to systematic reviews of interventions) to include and acknowledge the various sources of evidence that may be used in a scoping review (e.g., quantitative and/or qualitative research, expert opinion, and policy document).

**Supplementary Material 2. Search strategy and results.**

## Pubmed

1. Pre-hospital phase

| | |
|---|---|
| Search # | 1 |
| Database | Pubmed |
| Date | 30-08-2023 |
| Results | 448 |

| # | Category | Search string |
|---|---|---|
| 1 | **Study population** | |
| 2 | ACS | ("acute coronary syndrome*"[Title/Abstract] OR "ACS" [Title/Abstract] OR |
| 3 | MI | "myocardial infarction"[Title/Abstract] OR MI[Title/Abstract] OR infarct*[Title/Abstract] OR “heart attack”[Title/Abstract] OR “heart infarct*”[Title/Abstract] OR “cardiac infarct*”[Title/Abstract] OR |
| 4 | UA | "unstable angina"[Title/Abstract] OR UA[Title/Abstract] OR |
| 5 | (N)STE | STEMI[Title/Abstract] OR "ST-elevat*"[Title/Abstract] OR NSTEMI[Title/Abstract] or "nST-elevat*"[Title/Abstract] OR |
| 6 | Mesh | "Acute coronary syndrome"[Mesh] OR "Myocardial Infarction"[Mesh] |
| 7 | **Sex** | ) AND ( |
| 8 | Sex/gender | sex[Title/Abstract] OR “sex-“[Title/Abstract] OR "gender*"[Title/Abstract] OR |
| 9 | Mesh | “Sex factors”[Mesh] |
| 10 | **Phase** | ) AND ( |
| 11 | Pre-hospital | “prehospital” [Title/Abstract] OR “pre-hospital” [Title/Abstract] OR “out of hospital” [Title/Abstract] OR “out-of-hospital”[Title/Abstract] OR |
| 12 | Pre-admission | preadmission[Title/Abstract] OR pre-admission[Title/Abstract] OR |
| 13 | Ambulance | ambulance[Title/Abstract] OR |
| 14 | Home | home[Title/Abstract] |
| 15 | **Outcomes** | ) AND ( |
| 16 | Symptoms | symptom*[Title/Abstract] OR |
| 17 | Diagnosis | diagnosis[Title/Abstract] OR ECG[Title/Abstract] OR electrocardio*[Title/Abstract] OR |
| 18 | Treatment | care[Title/Abstract] OR defibrill*[Title/Abstract] OR treat*[Title/Abstract] OR manage*[Title/Abstract] OR |
| 19 | Fibrinolysis | fibrinolysis[Title/Abstract] OR thrombolysis[Title/Abstract] OR antiplatelet*[Title/Abstract] OR anti-platelet[Title/Abstract] OR aspirin[Title/Abstract] OR acetylsalicylic[Title/Abstract] OR anticoagulant[Title/Abstract] OR ”anti-platelet”[Title/Abstract] OR “platelet aggregation inhibitor*"[Title/Abstract] OR “platelet antiaggregant*"[Title/Abstract] OR “platelet inhibitor*"[Title/Abstract] OR |
| 21 | Anti-ischemic | anti-ischemic[Title/Abstract] OR antiischemic[Title/Abstract] OR |
| 22 | Beta-blocker | beta-blockers[Title/Abstract] OR |
| 23 | Nitrate | “nitrate*”[Title/Abstract] OR |
| 24 | Time | “Time”[Title/Abstract] OR “Time to treatment”[Title/Abstract] OR “Time to hospital”[Title/Abstract] OR “delay*”[Title/Abstract]) |

| | | |
|---|---|---|
| 25 | **Filters** | AND ((2013/1/1:2023/12/31[pdat]) AND (french[Filter] OR dutch[Filter] OR english[Filter] OR spanish[Filter])) NOT (Case reports[pt] OR Clinical Conference[pt] OR "animals"[mh:noexp]) |

2. Diagnosis phase

| | |
|---|---|
| Search # | 2 |
| Database | Pubmed |
| Date | 30-8-2023 |
| Results | 7090 |

| # | Category | Search string |
|---|---|---|
| 1 | **Study population** | |
| 2 | ACS | ("acute coronary syndrome*"[Title/Abstract] OR "ACS"[Title/Abstract] OR |
| 3 | MI | "myocardial infarction"[Title/Abstract] OR MI[Title/Abstract] OR infarct*[Title/Abstract] OR “heart attack”[Title/Abstract] OR “heart infarct*”[Title/Abstract] OR “cardiac infarct*”[Title/Abstract] OR |
| 4 | UA | "unstable angina"[Title/Abstract] OR UA[Title/Abstract] OR |
| 5 | (N)STE | STEMI[Title/Abstract] OR "ST-elevat*"[Title/Abstract] OR NSTEMI[Title/Abstract] or "nST-elevat*"[Title/Abstract] OR |
| 6 | Mesh | "Acute coronary syndrome"[Mesh] OR "Myocardial Infarction"[Mesh] |
| 7 | **Sex** | ) AND ( |
| 8 | Sex/gender | sex[Title/Abstract] OR “sex-“[Title/Abstract] OR "gender*"[Title/Abstract] OR |
| 9 | Mesh | “Sex factors”[Mesh] |
| 15 | **Outcomes** | ) AND ( |
| | Diagnosis | Diagn*[Title/Abstract] OR Underdiag* [Title/Abstract] OR misdiag* [Title/Abstract] "physical examination" [Title/Abstract] OR |
| | ECG | ECG[Title/Abstract] OR EKG[Title/Abstract] OR electrocardio*[Title/Abstract] OR |
| | Biomarkers | biomarker*[Title/Abstract] OR tropon*[Title/Abstract] OR |
| | Echocardiography | echocardio*[Title/Abstract] OR ultrasound[Title/Abstract] OR |
| | Tomography | CT[Title/Abstract] OR CCT[Title/Abstract] OR tomograph*[Title/Abstract] OR |
| | MR | CMR[Title/Abstract] OR MR[Title/Abstract] or "magnetic reson*"[Title/Abstract] OR |
| | PET or SPECT | PET[Title/Abstract] OR SPECT[Title/Abstract] OR |
| | Radiograph | radiograph*[Title/Abstract] OR "X-ray"[Title/Abstract] OR |
| | Angiography | CCTA[Title/Abstract] or ICA[Title/Abstract] OR angiography[Title/Abstract] OR |
| | Risk scores | "risk score*"[Title/Abstract] OR score*[Title/Abstract] OR "risk strat*"[Title/Abstract] OR "risk categor*"[Title/Abstract] OR |
| | Mesh | Electrocardiography[Mesh] OR Troponin[Mesh] OR “Cardiac Imaging Techniques"[Mesh]) |
| | **Filters** | AND ((2013/1/1:2023/12/31[pdat]) AND (french[Filter] OR dutch[Filter] OR english[Filter] OR spanish[Filter])) NOT (Case reports[pt] OR Clinical Conference[pt] OR "animals"[mh:noexp]) |

3. Treatment phase

| | |
|---|---|
| Search # | 3 |
| Database | Pubmed |
| Date | 30-08-23 |
| Results | 7160 |

| # | Category | Search string |
|---|---|---|
| 1 | **Study population** | |
| 2 | ACS | ("acute coronary syndrome*"[Title/Abstract] OR "ACS"[Title/Abstract] OR |
| 3 | MI | "myocardial infarction"[Title/Abstract] OR MI[Title/Abstract] OR infarct*[Title/Abstract] OR “heart attack”[Title/Abstract] OR “heart infarct*”[Title/Abstract] OR “cardiac infarct*”[Title/Abstract] OR |
| 4 | UA | "unstable angina"[Title/Abstract] OR UA[Title/Abstract] OR |
| 5 | (N)STE | STEMI[Title/Abstract] OR "ST-elevat*"[Title/Abstract] OR NSTEMI[Title/Abstract] or "nST-elevat*"[Title/Abstract] OR |
| 6 | Mesh | "Acute coronary syndrome"[Mesh] OR "Myocardial Infarction"[Mesh] |
| 7 | **Sex** | ) AND ( |
| 8 | Sex/gender | sex[Title/Abstract] OR “sex-“[Title/Abstract] OR "gender*"[Title/Abstract] OR |
| 9 | Mesh | “Sex factors”[Mesh] |
| 10 | **Outcomes** | ) AND ( |
| | Drug | Drug*[Title/Abstract] OR medicat*[Title/Abstract] OR pharmaco*[Title/Abstract] OR treat*[Title/Abstract] OR care*[Title/Abstract] OR manage*[Title/Abstract] OR |
| | Anti-thrombotic | antithrombotic*[Title/Abstract] OR anti-thrombotic*[Title/Abstract] OR anticoagulant*[Title/Abstract] OR anti-coagulant*[Title/Abstract] OR antiplatelet*[Title/Abstract] OR anti-platelet*[Title/Abstract] OR aspirin[Title/Abstract] OR “acetylsalicylic acid”[Title/Abstract] OR antiplatelet*[Title/Abstract] OR anti-platelet*[Title/Abstract] OR aspirin[Title/Abstract] OR “acetylsalicylic acid”[Title/Abstract] OR “P2Y12 receptor inhibitor*”[Title/Abstract] OR fibrinoly*[Title/Abstract] OR |
| | Anti-anginal | Anti-anginal[Title/Abstract] OR antianginal [Title/Abstract] OR |
| | Anti-ischemic | Anti-ischemic[Title/Abstract] OR antiischemic[Title/Abstract] OR |
| | Nitrate | “nitrate*”[Title/Abstract]OR |
| | Beta-blockers | “beta-blocker*”[Title/Abstract] OR “beta blocker*”[Title/Abstract] OR |
| | Revascularization | PCI[Title/Abstract] OR “percutaneous coronary intervention”[Title/Abstract] OR CABG[Title/Abstract] OR “coronary artery bypass graft*”[Title/Abstract] OR |
| | Mesh | “Percutaneous Coronary Intervention”[Mesh] OR “Coronary Artery Bypass”[Mesh] ) |
| | **Filters** | AND ((2013/1/1:2023/12/31[pdat]) AND (french[Filter] OR dutch[Filter] OR english[Filter] OR spanish[Filter])) NOT (Case reports[pt] OR Clinical Conference[pt] OR "animals"[mh:noexp]) |

4. Discharge phase

| | |
|---|---|
| Search # | 4 |
| Database | Pubmed |
| Date | 30-8-23 |
| Results | 1479 |

| # | Category | Search string |
|---|---|---|
| 1 | **Study population** | |
| 2 | ACS | ("acute coronary syndrome*"[Title/Abstract] OR "ACS" [Title/Abstract] OR |
| 3 | MI | "myocardial infarction"[Title/Abstract] OR MI[Title/Abstract] OR infarct*[Title/Abstract] OR "heart attack"[Title/Abstract] OR "heart infarct*"[Title/Abstract] OR "cardiac infarct*"[Title/Abstract] OR |
| 4 | UA | "unstable angina"[Title/Abstract] OR UA[Title/Abstract] OR |
| 5 | (N)STE | STEMI[Title/Abstract] OR "ST-elevat*"[Title/Abstract] OR NSTEMI[Title/Abstract] or "nST-elevat*"[Title/Abstract] OR |
| 6 | Mesh | "Acute coronary syndrome"[Mesh] OR "Myocardial Infarction"[Mesh] |
| 7 | **Sex** | ) AND ( |
| 8 | Sex/gender | sex[Title/Abstract] OR "sex-"[Title/Abstract] OR "gender*"[Title/Abstract] OR |
| 9 | Mesh | "Sex factors"[Mesh] |
| 10 | **Phase** | ) AND ( |
| | Discharge | Discharge*[Title/Abstract] |
| 10 | **Outcomes** | ) AND ( |
| | Drug prescription | Drug*[Title/Abstract] OR medicat*[Title/Abstract] OR pharmaco*[Title/Abstract] OR prescr*[Title/Abstract] OR treat*[Title/Abstract] OR care*[Title/Abstract] OR manage*[Title/Abstract] OR |
| | Advice | advice*[Title/Abstract] OR lifestyle*[Title/Abstract] OR |
| | Cardiac rehabilitation | "cardiac rehabilitation"[Title/Abstract] OR rehabilitation[Title/Abstract] OR referral[Title/Abstract] OR |
| | Mesh | "Cardiac Rehabilitation"[Mesh] ) |
| | **Filters** | AND ((2013/1/1:2023/12/31[pdat]) AND (french[Filter] OR dutch[Filter] OR english[Filter] OR spanish[Filter])) NOT (Case reports[pt] OR Clinical Conference[pt] OR "animals"[mh:noexp]) |

**Embase**

1. Pre hospital

| | |
|---|---|
| Search # | 5 |
| Database | Embase |
| Date | 30-8-2023 |
| Results | 72 |

| # | Category | Search string |
|---|---|---|
| 1 | **Study population** | |
| 2 | ACS | ('acute coronary syndrome':ti,ab OR 'ACS':ti,ab OR |
| 3 | MI | 'myocardial infarction':ti,ab OR 'MI':ti,ab OR 'infarct*':ti,ab OR 'heart attack':ti,ab OR 'heart infarct*':ti,ab OR 'cardiac infarct*':ti,ab OR |
| 4 | UA | 'unstable angina':ti,ab OR 'UA':ti,ab OR |
| 5 | (N)STE | 'STEMI':ti,ab OR 'ST-elevat*':ti,ab OR 'NSTEMI':ti,ab OR 'nST-elevat*':ti,ab OR |
| 6 | Mesh | 'Acute coronary syndrome'/exp OR 'heart infarction'/exp |
| 7 | **Sex** | ) AND ( |
| 8 | Sex/gender | 'sex':ti,ab OR 'sex-':ti,ab OR 'gender*':ti,ab OR |
| 9 | Mesh | 'Sex factor'/exp |

| | | |
|---|---|---|
| 10 | **Phase** | ) AND ( |
| 11 | Pre-hospital | 'prehospital':ti,ab OR 'pre-hospital':ti,ab OR 'out of hospital':ti,ab OR 'out-of-hospital':ti,ab OR |
| 12 | Pre-admission | 'preadmission':ti,ab OR 'pre-admission':ti,ab OR |
| 13 | Ambulance | 'ambulance':ti,ab OR |
| 14 | Home | 'home':ti,ab |
| 15 | **Outcomes** | ) AND ( |
| 16 | Symptoms | 'symptom*':ti,ab OR |
| 17 | Diagnosis | 'diagnosis':ti,ab OR 'ECG':ti,ab OR 'electrocardio*':ti,ab OR |
| 18 | Treatment | 'care*':ti,ab OR 'defibrill*':ti,ab OR 'treat*':ti,ab OR 'manage*':ti,ab OR |
| 19 | Fibrinolysis | 'fibrinolysis':ti,ab OR 'thrombolysis':ti,ab OR 'antiplatelet*':ti,ab OR 'anti-platelet':ti,ab OR 'aspirin':ti,ab OR 'acetylsalicylic':ti,ab OR 'anticoagulant':ti,ab OR 'anti-platelet':ti,ab OR 'platelet aggregation inhibitor*':ti,ab OR 'platelet antiaggregant*':ti,ab OR 'platelet inhibitor*':ti,ab OR |
| 20 | Mesh | 'Anticoagulant agent'/exp OR ‘fibrinolytic agent’/exp OR |
| 21 | Anti-ischemic | 'anti-ischemic':ti,ab OR 'antiischemic':ti,ab OR |
| 22 | Beta-blocker | 'beta-blockers':ti,ab OR |
| 23 | Nitrate | 'nitrate*':ti,ab OR 'dinitrate*':ti,ab OR 'isosorbide mononitrate':ti,ab OR 'isosorbide-5-mononitrate':ti,ab OR 'nitroglycerin':ti,ab OR 'glyceryl trinitrate':ti,ab OR |
| 24 | Time | 'Time':ti,ab OR 'Time to treatment':ti,ab OR 'Time to hospital':ti,ab OR 'delay*':ti,ab<br>) |
| 25 | **Filters** | AND ([french]/lim OR [dutch]/lim OR [english]/lim OR [spanish]/lim) AND [2013-2023]/py NOT ('conference abstract'/it OR 'conference review'/it OR 'case report'/de) AND [embase]/lim NOT ([embase]/lim AND [medline]/lim) AND 'human'/de |

2. Diagnosis

| | |
|---|---|
| Search # | 6 |
| Database | Embase |
| Date | 30-8-2023 |
| Results | 1882 |

| # | Category | Search string |
|---|---|---|
| 1 | **Study population** | |
| 2 | ACS | ('acute coronary syndrome':ti,ab OR 'ACS':ti,ab OR |
| 3 | MI | 'myocardial infarction':ti,ab OR 'MI':ti,ab OR 'infarct*':ti,ab OR 'heart attack':ti,ab OR 'heart infarct*':ti,ab OR 'cardiac infarct*':ti,ab OR |
| 4 | UA | 'unstable angina':ti,ab OR 'UA':ti,ab OR |
| 5 | (N)STE | 'STEMI':ti,ab OR 'ST-elevat*':ti,ab OR 'NSTEMI':ti,ab OR 'nST-elevat*':ti,ab OR |
| 6 | Mesh | 'Acute coronary syndrome'/exp OR ‘heart infarction'/exp |
| 7 | **Sex** | ) AND ( |
| 8 | Sex/gender | 'sex':ti,ab OR 'gender*':ti,ab OR 'sex-':ti,ab OR |
| 9 | Mesh | 'Sex factor'/exp |
| 15 | **Outcomes** | ) AND ( |

| | | |
|---|---|---|
| 16 | Diagnosis | 'Diagn*':ti,ab OR 'Underdiag*':ti,ab OR 'misdiag*':ti,ab OR 'physical examination':ti,ab OR |
| 17 | ECG | 'ECG':ti,ab OR 'EKG':ti,ab OR 'electrocardio*':ti,ab OR |
| 18 | Biomarkers | 'biomarker*':ti,ab OR 'tropon*':ti,ab OR 'high-sensitivity-troponin*':ti,ab OR 'hs-troponin*':ti,ab OR |
| 19 | Echocardiography | 'echocardio*':ti,ab OR 'ultrasound':ti,ab OR |
| 20 | Tomography | 'CT':ti,ab OR 'CCT':ti,ab OR 'tomograph*':ti,ab OR |
| 21 | MR | 'CMR':ti,ab OR 'MR':ti,ab OR 'magnetic reson*':ti,ab OR |
| 22 | PET or SPECT | 'PET':ti,ab OR 'SPECT':ti,ab OR |
| 23 | Radiograph | 'radiograph*':ti,ab OR 'X-ray':ti,ab OR |
| 24 | Angiography | 'CCTA':ti,ab OR 'ICA':ti,ab OR 'angiography':ti,ab OR |
| 25 | Risk scores | 'risk score*':ti,ab OR 'score':ti,ab OR 'GRACE':ti,ab OR 'EUROSCORE':ti,ab OR 'risk strat*':ti,ab OR 'risk categor*':ti,ab OR |
| | Mesh | 'Electrocardiography'/exp OR 'Troponin'/exp OR 'Cardiac Imaging'/exp ) |
| | **Filters** | AND ([french]/lim OR [dutch]/lim OR [english]/lim OR [spanish]/lim) AND [2013-2023]/py NOT ('conference abstract'/it OR 'conference review'/it OR 'case report'/de) AND [embase]/lim NOT ([embase]/lim AND [medline]/lim) AND 'human'/de |

3. Treatment

| | |
|---|---|
| Search # | 7 |
| Database | Embase |
| Date | 30-8-23 |
| Results | 1879 |

| # | Category | Search string |
|---|---|---|
| 1 | **Study population** | |
| 2 | ACS | ('acute coronary syndrome':ti,ab OR 'ACS':ti,ab OR |
| 3 | MI | 'myocardial infarction':ti,ab OR 'MI':ti,ab OR 'infarct*':ti,ab OR 'heart attack':ti,ab OR 'heart infarct*':ti,ab OR 'cardiac infarct*':ti,ab OR |
| 4 | UA | 'unstable angina':ti,ab OR 'UA':ti,ab OR |
| 5 | (N)STE | 'STEMI':ti,ab OR 'ST-elevat*':ti,ab OR 'NSTEMI':ti,ab OR 'nST-elevat*':ti,ab OR |
| 6 | Mesh | 'Acute coronary syndrome'/exp OR ‘heart infarction'/exp |
| 7 | **Sex** | ) AND ( |
| 8 | Sex/gender | 'sex':ti,ab OR 'gender*':ti,ab OR 'sex-':ti,ab OR |
| 9 | Mesh | 'Sex factor'/exp |
| 15 | **Outcomes** | ) AND ( |
| 16 | Drug | 'Drug*':ti,ab OR 'medicat*':ti,ab OR 'pharmaco*':ti,ab OR treat*:ti,ab OR care*:ti,ab OR 'manage*':ti,ab OR |
| 17 | Anti-thrombotic | 'antithrombotic*':ti,ab OR 'anti-thrombotic*':ti,ab OR 'anticoagulant*':ti,ab OR 'anti-coagulant*':ti,ab OR 'antiplatelet*':ti,ab OR 'anti-platelet*':ti,ab OR |
| 18 | Anti-anginal | 'aspirin':ti,ab OR 'acetylsalicylic acid':ti,ab OR 'P2Y12 receptor inhibitor*':ti,ab OR 'clopidogrel':ti,ab OR 'ticagrelor':ti,ab OR 'prasugrel':ti,ab OR 'Cangrelor':ti,ab OR 'GP IIb/IIIa receptor inhibitor*':ti,ab OR 'fibrinoly*':ti,ab OR |
| 19 | Anti-ischemic | 'Anti-anginal':ti,ab OR 'antianginal':ti,ab OR 'Anti-ischemic':ti,ab OR 'antiischemic':ti,ab OR |
| 20 | Nitrate | 'nitrate*':ti,ab OR 'isosorbide dinitrate*':ti,ab OR 'isosorbide mononitrate*':ti,ab OR 'nitroglycerin':ti,ab OR |

| | | |
|---|---|---|
| 21 | Beta-blockers | 'beta-blocker*':ti,ab OR 'beta blocker*':ti,ab OR 'atenolol':ti,ab OR 'bisoprolol':ti,ab OR 'carvedilol':ti,ab OR 'propanolol':ti,ab OR 'PCI':ti,ab OR |
| 22 | Revascularization | 'percutaneous coronary intervention':ti,ab OR 'CABG':ti,ab OR 'coronary artery bypass graft*':ti,ab OR |
| 23 | Mesh | 'Percutaneous Coronary Intervention'/exp OR 'Coronary Artery Bypass'/exp) |
| | **Filters** | AND ([french]/lim OR [dutch]/lim OR [english]/lim OR [spanish]/lim) AND [2013-2023]/py NOT ('conference abstract'/it OR 'conference review'/it OR 'case report'/de) AND [embase]/lim NOT ([embase]/lim AND [medline]/lim) AND 'human'/de |

4. Discharge and post-discharge

| | |
|---|---|
| Search # | 8 |
| Database | Embase |
| Date | 27-7-2023 |
| Results | 338 |

| # | Category | Search string |
|---|---|---|
| 1 | **Study population** | |
| 2 | ACS | ('acute coronary syndrome':ti,ab OR 'ACS':ti,ab OR |
| 3 | MI | 'myocardial infarction':ti,ab OR 'MI':ti,ab OR 'infarct*':ti,ab OR 'heart attack':ti,ab OR 'heart infarct*':ti,ab OR 'cardiac infarct*':ti,ab OR |
| 4 | UA | 'unstable angina':ti,ab OR 'UA':ti,ab OR |
| 5 | (N)STE | 'STEMI':ti,ab OR 'ST-elevat*':ti,ab OR 'NSTEMI':ti,ab OR 'nST-elevat*':ti,ab OR |
| 6 | Mesh | 'Acute coronary syndrome'/exp OR ‘heart infarction'/exp |
| 7 | **Sex** | ) AND ( |
| 8 | Sex/gender | 'sex':ti,ab OR 'gender*':ti,ab OR 'sex-':ti,ab OR |
| 9 | Mesh | 'Sex factor'/exp |
| 15 | **Phase** | ) AND ( |
| 16 | | 'Discharge*':ti,ab |
| 17 | **Outcomes** | ) AND ( |
| 18 | Drug prescription | 'Drug*':ti,ab OR 'medicat*':ti,ab OR 'pharmaco*':ti,ab OR 'prescr*':ti,ab OR 'treat*':ti,ab OR care*:ti,ab OR 'manage*':ti,ab OR |
| 19 | Advice | 'advice*':ti,ab OR 'lifestyle*':ti,ab OR |
| 20 | Cardiac rehabilitation | 'cardiac rehabilitation':ti,ab OR 'rehabilitation':ti,ab OR 'referral':ti,ab OR |
| 22 | Mesh | 'heart Rehabilitation'/exp ) |
| | **Filters** | AND ([french]/lim OR [dutch]/lim OR [english]/lim OR [spanish]/lim) AND [2013-2023]/py NOT ('conference abstract'/it OR 'conference review'/it OR 'case report'/de) AND [embase]/lim NOT ([embase]/lim AND [medline]/lim) AND 'human'/de |

**WHO Global Index Medicus**

1. Pre-hospital phase

| | |
|---|---|
| Search # | 9 |
| Database | WHO Global Index Medicus |

| | |
|---|---|
| Date | 30-8--2023 |
| Results | 515 |

tw:(tw:(("acute coronary syndrome" OR "acute coronary syndromes" OR acs OR "myocardial infarction" OR mi OR infarct* OR “heart attack” OR "unstable angina" OR ua OR stemi OR “st elevation” OR “st elevated” OR nstemi OR nst-elevation OR “st segment” OR “non-st elevation” OR “n-st elevated” OR “non-st elevated” )) OR mh:("Acute Coronary Syndrome" OR "Myocardial Infarction"))

AND (tw:(sex OR gender OR sex-))

AND (tw:(“prehospital” OR “pre-hospital” OR “out of hospital” OR “out-of-hospital” OR preadmission OR pre-admission OR ambulance OR home ))

AND (tw:(symptom* OR diagnosis OR ecg OR electrocardio* OR care OR defibrill* OR treat* OR care* OR manage* OR fibrinolysis OR thrombolysis OR antiplatelet* OR anti-platelet OR aspirin OR acetylsalicylic OR anticoagulant OR "anti-platelet" OR "platelet aggregation inhibitor" OR "platelet aggregation inhibitors" OR "platelet antiaggregant" OR "platelet antiaggregants" OR "platelet inhibitor" OR "platelet inhibitors" anti-ischemic OR antiischemic OR beta-blockers OR nitrate* OR "Time" OR "Time to treatment" OR "Time to hospital" OR delay*))

AND (year_cluster:[2013 TO 2023]) AND ( la:("en" OR "es" OR "fr"))

2. Diagnosis phase

| | |
|---|---|
| Search # | 10 |
| Database | WHO Global Index Medicus |
| Date | 30-8-2023 |
| Results | 606 |

tw:( ("acute coronary syndrome" OR "acute coronary syndromes" OR acs OR "myocardial infarction" OR mi OR infarct* OR “heart attack” OR "unstable angina" OR ua OR stemi OR “st elevation” OR “st elevated” OR nstemi OR nst-elevation OR “st segment” OR “non-st elevation” OR “n-st elevated” OR “non-st elevated” ) OR mh:("Acute Coronary Syndrome" OR "Myocardial Infarction"))

AND (tw:(sex OR gender OR sex-))

AND (tw:(diagn* OR underdiag* OR misdiag* "physical examination" OR ecg OR ekg OR electrocardio* OR biomarker* OR tropon* OR echocardio* OR ultrasound OR ct OR cct OR tomograph* OR cmr OR mr OR "magnetic resonance" OR pet OR spect OR radiograph* OR "X-ray" OR ccta OR ica OR angiography OR or score OR "risk stratification" OR "risk category" OR "risk categories"))

AND (year_cluster:[2013 TO 2023]) AND ( la:("en" OR "es" OR "fr"))

3. Treatment phase

| | |
|---|---|
| Search # | 11 |
| Database | WHO Global Index Medicus |
| Date | 30-8-23 |
| Results | 325 |

tw:( ("acute coronary syndrome" OR "acute coronary syndromes" OR acs OR "myocardial infarction" OR mi OR infarct* OR “heart attack” OR "unstable angina" OR ua OR stemi OR “st elevation” OR “st elevated” OR nstemi OR nst-elevation OR “st segment” OR “non-st elevation” OR “n-st elevated” OR “non-st elevated” ) OR mh:("Acute Coronary Syndrome" OR "Myocardial Infarction"))

AND (tw:(sex OR gender OR sex-))

AND

(tw:(drug* OR medicat* OR treat* OR care* OR manage*OR pharmaco* OR antithrombotic* OR anti-thrombotic* OR anticoagulant* OR anti-coagulant* OR antiplatelet* OR anti-platelet* OR aspirin OR "acetylsalicylic acid" OR antiplatelet* OR anti-platelet* OR aspirin OR "P2Y12 receptor inhibitor" OR "P2Y12 receptor inhibitors" OR fibrinoly* OR anti-anginal OR antianginal OR anti-ischemic OR antiischemic OR "nitrate" OR "nitrates" OR "beta-blocker" OR "beta-blockers" OR "beta blocker" OR "beta blockers" OR pci OR "percutaneous coronary intervention" OR cabg OR "coronary artery bypass graft" ) OR mh:("Percutaneous Coronary Intervention" OR "coronary artery bypass"))

AND (year_cluster:[2013 TO 2023]) AND ( la:("en" OR "es" OR "fr"))

4. Discharge phase

| Search # | 12 |
|---|---|
| Database | WHO Global Index Medicus |
| Date | 30-08-23 |
| Results | 32 |

tw:( ("acute coronary syndrome" OR "acute coronary syndromes" OR acs OR "myocardial infarction" OR mi OR infarct* OR “heart attack” OR "unstable angina" OR ua OR stemi OR “st elevation” OR “st elevated” OR nstemi OR nst-elevation OR “st segment” OR “non-st elevation” OR “n-st elevated” OR “non-st elevated” ) OR mh:("Acute Coronary Syndrome" OR "Myocardial Infarction"))

AND (tw:(sex OR gender OR sex-))

AND
(tw:(discharge*))

AND

((tw:(drug* OR medicat* OR pharmaco* OR prescr* OR treat* OR manage* OR care* OR advice* OR lifestyle* OR “cardiac rehabilitation” OR rehabilitation OR referral)) OR (mh:("Cardiac rehabilitation")))

AND (year_cluster:[2013 TO 2023]) AND ( la:("en" OR "es" OR “fr”))

**Supplementary Material 3. Examples of outcomes.**

| Phase | Topics | Direction | Example |
|---|---|---|---|
| Pre-hospital | Time to medical attention | Favors men | Women have longer time from symptom onset to admission (1) |
| | | Mixed | Females had longer call-to-hospital time, scene time, and ECG to hospital time. No significant sex differences in scene-to ECG time (2). |
| | | No differences | No significant differences in ambulance time (call to scene, on scene, on scene to hospital, and call to hospital) in multivariable analysis (3). |
| | Diagnosis and treatment | Favors men | Females were less likely to receive preadmission treatment p <0.001 (4). |
| | | Favors women | Women more likely to use emergency medical services (5). |
| | | Mixed | Women less likely to be assessed for ACS, MI, and cardiac arrest. No significant differences in agnina (6). |
| | | No differences | No association between aspirin administration by gender (7). |
| | Events | Favors men | Female sex associated with pre-hospital death in multivariate analyses (8). |
| | | Favors women | Women less frequently had a prehospital cardiac arrest compared to men (9). |
| | | No differences | No sex differences in out-of-hospital cardiac arrest (10). |
| Diagnosis | Time | Favors men | Women were less likely to have ECG <10min (p=0.013) (11). |
| | Diagnostic tests including biomarkers, ECG, angiography, echochardiography, imaging | Favors men | Women were less likely to receive ECG (12). |
| | | Favors women | Women were more likely to have angiography (13). |
| | | Mixed | Women were less likely to receive invasive angiography, there were no sex differences in stress testing, women were more likely to receive echocardiography (14). |
| | | No differences | There were no sex differences in ECG in the adjusted analysis (15). |
| Treatment | Time | Favors men | Female sex was associated with defined as door to balloon time >90 or >120 minutes for transfer or door to needle time>30 minutes (16). |
| | | Favors women | Women had shorter length of stay (12 ± 14 vs. 13 ± 14 days)(17). |
| | | Mixed | More women had total ischemic time >4h, there were no significant differences in door to balloon time >120min (18). |
| | | No differences | There were no significant sex differences in length of stay (p = 0.093). (19) |
| | Revascularization and reperfusion (in STEMI patients) | Favors men | Less women underwent PCI and CABG. p<0.01 (20) |
| | | Favors women | Women more likely to have drug-eluting stents (21) |
| | | No differences | Not significant differences in PCI, CABG and no interevntion rates (22) |
| | Medication | Favors men | Women were less likely to receive thrombolysis (23). |
| | | Favors women | Women received less beta-blockers and ACE inhibitors (24). |
| | | Mixed | Women were less likely to receive aspirin, P2Y12 antagonist, dual-antiplatelet therapy, and beta-blockers, but more likely to receive statins. (25) |
| | | No differences | No significant differences in optimal medical therapy (26). |
| | Other | Favors men | Mechanical circulatory support was used more frequently in men, p < 0.001 (27). |
| | | Favors women | Use of extra-corporeal membrane oxygenation is significantly higher in women (28). |
| | | Mixed | Women more likely to undergo preprocedural shock and circulatory support less likely to undergo preprocedural ventialtion (29). |
| | | No differences | There were no sex differences in ionotrope IV use (30). |
| Events | | Favors men | Women had higher in-hospital mortality ( p<0.001)(31). |

| | | | |
|---|---|---|---|
| | | Favors women | Females had lower mortality (32). |
| | Complications and in-hospital mortality | Mixed | Atrial fibrillation (in NSTEMI and STEMI) and cardiogenic shock (STEMI) were more common in women (p<0.001). Among NSTEMI patients, differences in cardiogenic shock not significant (33). |
| | | No differences | There were no significant sex differences in in-hospital mortality (34). |
| Discharge | Medication prescription | Favors men | Women were less likely to be discharged on beta blockers (35). |
| | | Favors women | Women were prescribed more statins (36). |
| | | Mixed | Women had lower prescriptions of ASA, clopidogrel, beta-blockers, lipid-lowering drugs. There were no significant differences for anticoagulants, triple therapy, and ACE inhibitors (37). |
| | | No differences | Not significant differences in dual antiplatelet therapy, statin, beta-blockers, ACEI/ARB (38). |
| | Provision of advice | Favors men | Less women received dietary advice (39). |
| | | No differences | There were no significant sex differences in advice on diet and exercise at discharge. (40) |
| | Cardiac rehabilitation | Favors men | Women had lower referral to CR (41). |
| | | No differences | There were no significant differences in cardiac rehabilitation (39). |

## References


1. Hao Y, Liu J, Liu J, Yang N, Smith SC, Huo Y, et al. Sex Differences in In-Hospital Management and Outcomes of Patients With Acute Coronary Syndrome. Circulation. 2019 Apr 9;139(15):1776–85.
2. Alrawashdeh A, Nehme Z, Williams B, Smith K, Stephenson M, Bernard S, et al. Factors associated with emergency medical service delays in suspected ST-elevation myocardial infarction in Victoria, Australia: A retrospective study. Emergency Medicine Australasia. 2020 Oct 9;32(5):777–85.
3. Coventry LL, Bremner AP, Jacobs IG, Finn J. Myocardial Infarction: Sex Differences in Symptoms Reported to Emergency Dispatch. Prehospital Emergency Care. 2013 Mar 6;17(2):193–202.
4. Leurent G, Garlantézec R, Auffret V, Hacot JP, Coudert I, Filippi E, et al. Gender differences in presentation, management and inhospital outcome in patients with ST-segment elevation myocardial infarction: Data from 5000 patients included in the ORBI prospective French regional registry. Arch Cardiovasc Dis. 2014 May;107(5):291–8.
5. Quinn T, Johnsen S, Gale CP, Snooks H, McLean S, Woollard M, et al. Effects of prehospital 12-lead ECG on processes of care and mortality in acute coronary syndrome: a linked cohort study from the Myocardial Ischaemia National Audit Project. Heart. 2014 Jun 15;100(12):944–50.
6. Hsu B, Carcel C, Wang X, Peters SAE, Randall DA, Havard A, et al. Sex differences in emergency medical services management of patients with myocardial infarction: analysis of routinely collected data for over 110,000 patients. Am Heart J. 2021 Nov;241:87–91.
7. Tataris KL, Mercer MP, Govindarajan P. Prehospital aspirin administration for acute coronary syndrome (ACS) in the USA: an EMS quality assessment using the NEMSIS 2011 database. Emergency Medicine Journal. 2015 Nov;32(11):876–81.
8. Toshima T, Hirayama A, Watanabe T, Goto J, Kobayashi Y, Otaki Y, et al. Unmet needs for emergency care and prevention of prehospital death in acute myocardial infarction. J Cardiol. 2021 Jun;77(6):605–12.
9. Wilkinson C, Bebb O, Dondo TB, Munyombwe T, Casadei B, Clarke S, et al. Sex differences in quality indicator attainment for myocardial infarction: A nationwide cohort study. Heart. 2019 Apr 1;105(7):516–23.
10. Velders MA, Boden H, van Boven AJ, van der Hoeven BL, Heestermans AACM, Cannegieter SC, et al. Influence of Gender on Ischemic Times and Outcomes After ST-Elevation Myocardial Infarction. Am J Cardiol. 2013 Feb;111(3):312–8.
11. Chou LP, Zhao P, Kao C, Chen YH, Jong GP. Women were noninferior to men in cardiovascular outcomes among patients with ST-segment elevation myocardial infarction treated with primary percutaneous coronary intervention from Taiwan acute coronary syndrome full-spectrum registry. Medicine. 2018 Oct;97(43):e12998.
12. Lawesson SS, Alfredsson J, Fredrikson M, Swahn E. A gender perspective on short- and long term mortality in ST-elevation myocardial infarction — A report from the SWEDEHEART register. Int J Cardiol. 2013 Sep;168(2):1041–7.
13. Díez-Villanueva P, García-Acuña JM, Raposeiras-Roubin S, Barrabés JA, Cordero A, Martínez-Sellés M, et al. Prognosis Impact of Diabetes in Elderly Women and Men with Non-ST Elevation Acute Coronary Syndrome. J Clin Med. 2021 Sep 26;10(19):4403.
14. Arora S, Stouffer GA, Kucharska-Newton AM, Qamar A, Vaduganathan M, Pandey A, et al. Twenty Year Trends and Sex Differences in Young Adults Hospitalized With Acute Myocardial Infarction. Circulation. 2019 Feb 19;139(8):1047–56.
15. Banco D, Chang J, Talmor N, Wadhera P, Mukhopadhyay A, Lu X, et al. Sex and Race Differences in the Evaluation and Treatment of Young Adults Presenting to the Emergency Department With Chest Pain. J Am Heart Assoc. 2022 May 17;11(10).
16. Gupta A, Barrabes JA, Strait K, Bueno H, Porta-Sánchez A, Acosta-Vélez JG, et al. Sex Differences in Timeliness of Reperfusion in Young Patients With ST-Segment–Elevation Myocardial Infarction by Initial Electrocardiographic Characteristics. J Am Heart Assoc. 2018 Mar 20;7(6).
17. Vallabhajosyula S, Ya'Qoub L, Dunlay SM, Vallabhajosyula S, Vallabhajosyula S, Sundaragiri PR, Jaffe AS, Gersh BJ, Kashani K. Sex disparities in acute kidney injury complicating acute myocardial infarction with cardiogenic shock. ESC Heart Fail. 2019 Aug;6(4):874-877. doi: 10.1002/ehf2.12482. Epub 2019 Jul 4. PMID: 31271517; PMCID: PMC6676281.
18. Gevaert SA, De Bacquer D, Evrard P, Renard M, Beauloye C, Coussement P, et al. Renal dysfunction in STEMI-patients undergoing primary angioplasty: higher prevalence but equal prognostic impact in female patients; an observational cohort study from the Belgian STEMI registry. BMC Nephrol. 2013 Dec 18;14(1):62.
19. Wei J, Mehta PK, Grey E, Garberich RF, Hauser R, Bairey Merz CN, et al. Sex-based differences in quality of care and outcomes in a health system using a standardized STEMI protocol. Am Heart J. 2017 Sep;191:30–6.
20. Bucholz EM, Strait KM, Dreyer RP, Lindau ST, D'Onofrio G, Geda M, et al. Editor's Choice-Sex differences in young patients with acute myocardial infarction: A VIRGO study analysis. Eur Heart J Acute Cardiovasc Care. 2017 Oct 2;6(7):610–22.
21. Bhasin V, Hiltner E, Singh A, Elsaid O, Awasthi A, Kassotis J, et al. Disparities in Drug-Eluting Stent Utilization in Patients With Acute ST-Elevation Myocardial Infarction: An Analysis of the National Inpatient Sample. Angiology. 2023 Sep 17;74(8):774–82.
22. Ghadri JR, Sarcon A, Jaguszewski M, Diekmann J, Bataiosu RD, Hellermann J, et al. Gender disparities in acute coronary syndrome. Journal of Cardiovascular Medicine. 2015 May;16(5):355–62.
23. Matetic A, Shamkhani W, Rashid M, Volgman AS, Van Spall HGC, Coutinho T, et al. Trends of Sex Differences in Clinical Outcomes After Myocardial Infarction in the United States. CJC Open. 2021 Dec;3(12):S19–27.
24. Bugiardini R, Ricci B, Cenko E, Vasiljevic Z, Kedev S, Davidovic G, et al. Delayed Care and Mortality Among Women and Men With Myocardial Infarction. J Am Heart Assoc. 2017 Aug 2;6(8).

25. Wang S, Zhang Y, Cheng Q, Qi D, Wang X, Zhu Z, et al. Sex Disparity in Characteristics, Management, and In-Hospital Outcomes of Patients with ST-Segment Elevated Myocardial Infarction: Insights from Henan STEMI Registry. Cardiol Res Pract. 2022 Sep 5;2022:1–10.
26. García-García C, Molina L, Subirana I, Sala J, Bruguera J, Arós F, et al. Sex-based Differences in Clinical Features, Management, and 28-day and 7-year Prognosis of First Acute Myocardial Infarction. RESCATE II Study. Revista Española de Cardiología (English Edition). 2014 Jan;67(1):28–35.
27. Vallabhajosyula S, Dunlay SM, Barsness GW, Miller PE, Cheungpasitporn W, Stulak JM, et al. Sex Disparities in the Use and Outcomes of Temporary Mechanical Circulatory Support for Acute Myocardial Infarction-Cardiogenic Shock. CJC Open. 2020 Nov;2(6):462–72.
28. Lin DSH, Lin YS, Lee JK, Kao HL. Sex differences following percutaneous coronary intervention or coronary artery bypass surgery for acute myocardial infarction. Biol Sex Differ. 2022 Dec 27;13(1):18.
29. Kwok CS, Kontopantelis E, Kunadian V, Anderson S, Ratib K, Sperrin M, et al. Effect of access site, gender, and indication on clinical outcomes after percutaneous coronary intervention: Insights from the British Cardiovascular Intervention Society (BCIS). Am Heart J. 2015 Jul;170(1):164-172.e5.
30. Redfors B, Angerås O, Råmunddal T, Petursson P, Haraldsson I, Dworeck C, et al. Trends in Gender Differences in Cardiac Care and Outcome After Acute Myocardial Infarction in Western Sweden: A Report From the Swedish Web System for Enhancement of Evidence-Based Care in Heart Disease Evaluated According to Recommended Therapies (SWEDEHEART). J Am Heart Assoc. 2015 Jul 17;4(7).
31. Alexander T, Victor SM, Jayakumaran B, Rajan S, Mullasari S A. Sex-Related Differences in Outcomes for Patients With ST Elevation Myocardial Infarction (STEMI): A Tamil Nadu-STEMI Program Subgroup Analysis. Heart Lung Circ. 2021 Dec;30(12):1870–5.
32. Ariss RW, Elzanaty AM, Minhas AMK, Nazir S, Gul S, Patel N, et al. Sex-based differences in clinical outcomes and resource utilization of type 2 myocardial infarction. Int J Cardiol. 2021 Sep;338:24–9.
33. de Miguel-Yanes JM, Jiménez-García R, Hernandez-Barrera V, de Miguel-Díez J, Muñoz-Rivas N, Méndez-Bailón M, et al. Sex Differences in the Incidence and Outcomes of Acute Myocardial Infarction in Spain, 2016–2018: A Matched-Pair Analysis. J Clin Med. 2021 Apr 20;10(8):1795.
34. Bandyopadhyay D, Chakraborty S, Amgai B, Patel N, Hajra A, Heise L, et al. Acute myocardial infarction in the young - National Trend Analysis with gender-based difference in outcomes. Int J Cardiol. 2020 Feb;301:21–8.
35. Dafaalla M, Rashid M, Van Spall HGC, Mehta L, Parwani P, Sharma G, et al. Women Are Less Likely to Survive AMI Presenting With Out-of-Hospital Cardiac Arrest. Mayo Clin Proc. 2022 Sep;97(9):1608–18.
36. Lin CF, Shen LJ, Hsiao FY, Gau CS, Wu FLL. Sex Differences in the Treatment and Outcome of Patients with Acute Coronary Syndrome After Percutaneous Coronary Intervention: A Population-Based Study. J Womens Health. 2014 Mar;23(3):238–45.
37. Birkemeyer R, Schneider H, Rillig A, Ebeling J, Akin I, Kische S, et al. Do gender differences in primary PCI mortality represent a different adherence to guideline recommended therapy? a multicenter observation. BMC Cardiovasc Disord. 2014 Dec 2;14(1):71.
38. Gao S, Ma W, Huang S, Lin X, Yu M. Sex-Specific Clinical Characteristics and Long-Term Outcomes in Patients With Myocardial Infarction With Non-obstructive Coronary Arteries. Front Cardiovasc Med. 2021 Jun 9;8.
39. Langabeer JR, Henry TD, Fowler R, Champagne-Langabeer T, Kim J, Jacobs AK. Sex-Based Differences in Discharge Disposition and Outcomes for ST-Segment Elevation Myocardial Infarction Patients Within a Regional Network. J Womens Health. 2018 Aug;27(8):1001–6.
40. Bakhai A, Ferrières J, James S, Iñiguez A, Mohácsi A, Pavlides G, et al. Treatment, Outcomes, Costs, and Quality of Life of Women and Men With Acute Coronary Syndromes Who Have Undergone Percutaneous Coronary Intervention: Results From the Antiplatelet Therapy Observational Registry. Postgrad Med. 2013 Mar 13;125(2):100–7.
41. Khan E, Brieger D, Amerena J, Atherton JJ, Chew DP, Farshid A, et al. Differences in management and outcomes for men and women with ST-elevation myocardial infarction. Medical Journal of Australia. 2018 Aug 23;209(3):118–23.

**Supplementary Material 4. Bibliography.**


1.
Abdel-Qadir HM, Ivanov J, Austin PC, Tu JV, Džavík V. Sex differences in the management and outcomes of Ontario patients with cardiogenic shock complicating acute myocardial infarction. Can J Cardiol. 2013 Jun;29(6):691–6.
2.
Aguiar Rosa SA, Timóteo AT, Nogueira MA, Belo A, Ferreira RC. Acute coronary syndrome in elderly – What is the place for invasive strategy? European Geriatric Medicine. 2017;8(1):90–5.
3.
Aguilar SA, Patel M, Castillo E, Patel E, Fisher R, Ochs G, et al. Gender differences in scene time, transport time, and total scene to hospital arrival time determined by the use of a prehospital electrocardiogram in patients with complaint of chest pain. J Emerg Med. 2012 Aug;43(2):291–7.
4.
Ahmadi A, Khaledifar A, Sajjadi H, Soori H. Relationship between risk factors and in-hospital mortality due to myocardial infarction by educational level: a national prospective study in Iran. Int J Equity Health. 2014 Nov;13:116.
5.
Ahmadi A, Soori H, Mehrabi Y, Etemad K, Khaledifar A. Epidemiological pattern of myocardial infarction and modelling risk factors relevant to in-hospital mortality: the first results from the Iranian Myocardial Infarction Registry. Kardiol Pol. 2015;73(6):451–7.
6.
Ahmadi A, Soori H, Mehrabi Y, Etemad K, Sajjadi H, Sadeghi M. Predictive Factors of Hospital Mortality Due to Myocardial Infarction: A Multilevel Analysis of Iran's National Data. Int J Prev Med. 2015;6:112.
7.
Ahmadi A, Soori H, Sajjadi H, Nasri H, Mehrabi Y, Etemad K. Current status of the clinical epidemiology of myocardial infarction in men and women: a national cross-sectional study in iran. Int J Prev Med. 2015;6:14.
8.
Akimbaeva Z, Ismailov Z, Akanov AA, Radišauskas R, Padaiga Ž. Assessment of coronary care management and hospital mortality from ST-segment elevation myocardial infarction in the Kazakhstan population: Data from 2012 to 2015. Medicina (Kaunas). 2017;53(1):58–65.
9.
Al-Aqeedi RF, Al Suwaidi J, Singh R, Al Binali HA. Does prior coronary artery bypass surgery alter the gender gap in patients presenting with acute coronary syndrome? A 20-year retrospective cohort study. BMJ Open. 2012;2(6).
10.
Al-Fiadh AH, Andrianopoulos N, Farouque O, Yan BP, Duffy SJ, Charter K, et al. Contemporary outcomes in women undergoing percutaneous coronary intervention for acute coronary syndromes. Int J Cardiol. 2011 Sep;151(2):195–9.
11.
Alajmi H, Zubaid M, Rashed W, Al-Zakwani I. Compliance with guideline-recommended management in patients with non-st-elevation acute coronary syndromes: Findings from the gulf COAST registry. Annals of Clinical Cardiology. 2021;3(1):8–13.
12.
Alexander T, Victor SM, Jayakumaran B, Rajan S, Mullasari SA. Sex-Related Differences in Outcomes for Patients With ST Elevation Myocardial Infarction (STEMI): A Tamil Nadu-STEMI Program Subgroup Analysis. Heart Lung Circ. 2021 Dec;30(12):1870–5.
13.
Alkhouli M, Alqahtani F, Elsisy MF, Kawsara A, Alasnag M. Incidence and Outcomes of Acute Ischemic Stroke Following Percutaneous Coronary Interventions in Men Versus Women. Am J Cardiol. 2020 Feb;125(3):336–40.
14.
Alkhouli M, Alqahtani F, Jneid H, Al Hajji M, Boubas W, Lerman A. Age-Stratified Sex-Related Differences in the Incidence, Management, and Outcomes of Acute Myocardial Infarction. Mayo Clin Proc. 2021 Feb;96(2):332–41.

15.
Alnsasra H, Zahger D, Geva D, Matetzky S, Beigel R, Iakobishvili Z, et al. Contemporary Determinants of Delayed Benchmark Timelines in Acute Myocardial Infarction in Men and Women. Am J Cardiol. 2017 Nov;120(10):1715–9.
16.
Alrawashdeh A, Nehme Z, Williams B, Smith K, Stephenson M, Bernard S, et al. Factors associated with emergency medical service delays in suspected ST-elevation myocardial infarction in Victoria, Australia: A retrospective study. Emerg Med Australas. 2020 Oct;32(5):777–85.
17.
Amann U, Kirchberger I, Heier M, Thilo C, Kuch B, Peters A, et al. Predictors of non-invasive therapy and 28-day-case fatality in elderly compared to younger patients with acute myocardial infarction: an observational study from the MONICA/KORA Myocardial Infarction Registry. BMC Cardiovasc Disord. 2016 Jul;16:151.
18.
Ambrosino M, Emerson S, Catalano L, Hossain E, Julien HM, Jacoby DS. Post-Acute Coronary Syndrome Disparities in Guideline-Directed Lipid Therapy and Insufficient Achievement of Optimal Low-Density Lipoprotein. Am J Cardiol. 2023 Mar;190:8–16.
19.
Amon J, Wong GC, Lee T, Singer J, Cairns J, Shavadia JS, et al. Incidence and Predictors of Adverse Events Among Initially Stable ST-Elevation Myocardial Infarction Patients Following Primary Percutaneous Coronary Intervention. J Am Heart Assoc. 2022 Sep;11(17):e025572.
20.
Anandan H, Maharajan R, Begam S, Victor SM. Sex-Based Difference in Clinical Presentation and Outcomes-A Single-Center Experience. Indian Journal of Cardiovascular Disease in Women - WINCARS. 2021;6(4):241–7.
21.
Araújo C, Pereira M, Laszczyńska O, Dias P, Azevedo A. Sex-related inequalities in management of patients with acute coronary syndrome-results from the EURHOBOP study. Int J Clin Pract. 2018 Jan;72(1).
22.
Arias-Mendoza A, González-Pacheco H, Álvarez-Sangabriel A, Gopar-Nieto R, Rodríguez-Chávez LL, Araiza-Garaygordobil D, et al. Women with Acute Myocardial Infarction: Clinical Characteristics, Treatment, and In-Hospital Outcomes from a Latin American Country. Glob Heart. 2023;18(1):19.
23.
Ariss RW, Elzanaty AM, Minhas AMK, Nazir S, Gul S, Patel N, et al. Sex-based differences in clinical outcomes and resource utilization of type 2 myocardial infarction. Int J Cardiol. 2021 Sep;338:24–9.
24.
Arora S, Stouffer GA, Kucharska-Newton AM, Qamar A, Vaduganathan M, Pandey A, et al. Twenty Year Trends and Sex Differences in Young Adults Hospitalized With Acute Myocardial Infarction. Circulation. 2019 Feb;139(8):1047–56.
25.
Arslani K, Tontsch J, Todorov A, Gysi B, Kaufmann M, Kaufmann F, et al. Temporal trends in mortality and provision of intensive care in younger women and men with acute myocardial infarction or stroke. Crit Care. 2023 Jan;27(1):14.
26.
Arzuan M, Iram YA, Matetzky S, Herscovici R, Goldkorn R, Goitein O, et al. Sex differences of patients with acute chest pain evaluated through a chest pain unit. J Cardiovasc Med (Hagerstown). 2023 May;24(5):283–8.
27.
Ashraf M, Jan MF, Bajwa TK, Carnahan R, Zlochiver V, Allaqaband SQ. Sex Disparities in Diagnostic Evaluation and Revascularization in Patients With Acute Myocardial Infarction-A 15-Year Nationwide Study. J Am Heart Assoc. 2023 Mar;12(6):e027716.
28.
Atzema CL, Austin PC, Tu JV, Schull MJ. ED triage of patients with acute myocardial infarction: predictors of low acuity triage. Am J Emerg Med. 2010 Jul;28(6):694–702.
29.

Austin D, Yan AT, Spratt JC, Kunadian V, Edwards RJ, Egred M, et al. Patient characteristics associated with self-presentation, treatment delay and survival following primary percutaneous coronary intervention. Eur Heart J Acute Cardiovasc Care. 2014 Sep;3(3):214–22.
30.
Ayhan E, Isik T, Uyarel H, Ergelen R, Cicek G, Ghannadian B, et al. Femoral pseudoaneurysm in patients undergoing primary percutaneous coronary intervention for ST-elevation myocardial infarction: incidence, clinical course and risk factors. Int Angiol. 2012 Dec;31(6):579–85.
31.
Bahall M, Seemungal T, Khan K, Legall G. Medical care of acute myocardial infarction patients in a resource limiting country, Trinidad: a cross-sectional retrospective study. BMC Health Serv Res. 2019 Jul;19(1):501.
32.
Bainey KR, Alemayehu W, Gupta AK, Bowker SL, Welsh RC, Kaul P. Ethnic and sex differences in ambulance activation among hospitalized patients with acute coronary syndromes: Insights from the Alberta contemporary acute coronary syndrome patients invasive treatment strategies (COAPT) study. Int J Cardiol. 2018 Dec;272:33–9.
33.
Bakhai A, Ferrières J, James S, Iñiguez A, Mohácsi A, Pavlides G, et al. Treatment, outcomes, costs, and quality of life of women and men with acute coronary syndromes who have undergone percutaneous coronary intervention: results from the antiplatelet therapy observational registry. Postgrad Med. 2013 Mar;125(2):100–7.
34.
Balen F, Lhermusier T, Grolleau S, Pélissier F, Dehours E, Charpentier S, et al. Identifying key factors leading to the optimal care pathway for patients with ST-segment elevation myocardial infarction: Results from the RESCAMIP registry. Arch Cardiovasc Dis. 2019 Jun;112(6):374–80.
35.
Banco D, Chang J, Talmor N, Wadhera P, Mukhopadhyay A, Lu X, et al. Sex and Race Differences in the Evaluation and Treatment of Young Adults Presenting to the Emergency Department With Chest Pain. J Am Heart Assoc. 2022 May;11(10):e024199.
36.
Bandyopadhyay D, Chakraborty S, Amgai B, Patel N, Hajra A, Heise L, et al. Acute myocardial infarction in the young - National Trend Analysis with gender-based difference in outcomes. Int J Cardiol. 2020 Feb;301:21–8.
37.
Barr P, Smyth D, Harding SA, El-Jack S, Williams MJ, Devlin G, et al. Variation in Arterial Access for Invasive Coronary Procedures in New Zealand: A National Analysis (ANZACS-QI 5). Heart Lung Circ. 2016 May;25(5):451–8.
38.
Basra SS, Wang TY, Simon DN, Chiswell K, Virani SS, Alam M, et al. Ticagrelor Use in Acute Myocardial Infarction: Insights From the National Cardiovascular Data Registry. J Am Heart Assoc. 2018 Jun;7(12).
39.
Bataille Y, Déry JP, Larose É, Abdelaal E, Machaalany J, Rodés-Cabau J, et al. Incidence and clinical impact of concurrent chronic total occlusion according to gender in ST-elevation myocardial infarction. Catheter Cardiovasc Interv. 2013 Jul;82(1):19–26.
40.
Baviera M, Santalucia P, Cortesi L, Marzona I, Tettamanti M, Avanzini F, et al. Sex differences in cardiovascular outcomes, pharmacological treatments and indicators of care in patients with newly diagnosed diabetes: Analyses on administrative database. Eur J Intern Med. 2014 Mar;25(3):270–5.
41.
Benamer H, Bataille S, Tafflet M, Jabre P, Dupas F, Laborne FX, et al. Longer pre-hospital delays and higher mortality in women with STEMI: the e-MUST Registry. EuroIntervention. 2016 Aug;12(5):e542-9.
42.
Bender U, Norris CM, Dreyer RP, Krumholz HM, Raparelli V, Pilote L. Impact of Sex- and Gender-Related Factors on Length of Stay Following Non-ST-Segment-Elevation Myocardial Infarction: A Multicountry Analysis. J Am Heart Assoc. 2023 Aug;12(15):e028553.
43.

Berlin C, Jüni P, Endrich O, Zwahlen M. Revascularization Treatment of Emergency Patients with Acute ST-Segment Elevation Myocardial Infarction in Switzerland: Results from a Nationwide, Cross-Sectional Study in Switzerland for 2010-2011. PLoS One. 2016;11(4):e0153326.
44.
Bessonov IS, Kuznetsov VA, Gorbatenko EA, Dyakova AO, Sapozhnikov SS. Influence of Total Ischemic Time on Clinical Outcomes in Patients with ST-Segment Elevation Myocardial Infarction. Kardiologiia. 2021 Mar;61(2):40–6.
45.
Bhardwaj B, Sidhu G, Balla S, Kumar V, Kumar A, Aggarwal K, et al. Outcomes and Hospital Utilization in Patients With Papillary Muscle Rupture Associated With Acute Myocardial Infarction. Am J Cardiol. 2020 Apr;125(7):1020–5.
46.
Bhasin V, Hiltner E, Singh A, Elsaid O, Awasthi A, Kassotis J, et al. Disparities in Drug-Eluting Stent Utilization in Patients With Acute ST-Elevation Myocardial Infarction: An Analysis of the National Inpatient Sample. Angiology. 2023 Sep;74(8):774–82.
47.
Bierman AS, Brown AD, Levinton CM. Using decision trees for measuring gender equity in the timing of angiography in patients with acute coronary syndrome: a novel approach to equity analysis. Int J Equity Health. 2015 Dec;14:155.
48.
Birkemeyer R, Schneider H, Rillig A, Ebeling J, Akin I, Kische S, et al. Do gender differences in primary PCI mortality represent a different adherence to guideline recommended therapy? a multicenter observation. BMC Cardiovasc Disord. 2014 Jun;14:71.
49.
Blusztein D, Dinh D, Stub D, Dawson L, Brennan A, Reid C, et al. Predictors of hospital prenotification for STEMI and association of prenotification with outcomes. Emerg Med J. 2022 Sep;39(9):666–71.
50.
Bouisset F, Deney A, Ferrières J, Panagides V, Becker M, Riviere N, et al. Mechanical complications in ST-elevation myocardial infarction: The impact of pre-hospital delay. Int J Cardiol. 2021 Dec;345:14–9.
51.
Breuckmann F, Settelmeier S, Rassaf T, Hochadel M, Nowak B, Voigtländer T, et al. Unexpected high level of severe events even in low-risk profile chest pain unit patients. Herz. 2022 Aug;47(4):374–9.
52.
Brown RA, Shantsila E, Varma C, Lip GY. Symptom-to-door times in patients presenting with ST elevation myocardial infarction–do ethnic or gender differences exist? Qjm. 2016 Mar;109(3):175–80.
53.
Brush Jr JE, Chaudhry SI, Dreyer RP, D'Onofrio G, Greene EJ, Hajduk AM, et al. Sex Differences in Symptom Complexity and Door-to-Balloon Time in Patients With ST-Elevation Myocardial Infarction. Am J Cardiol. 2023 Jun;197:101–7.
54.
Bucholz EM, Strait KM, Dreyer RP, Lindau ST, D'Onofrio G, Geda M, et al. Editor's Choice-Sex differences in young patients with acute myocardial infarction: A VIRGO study analysis. Eur Heart J Acute Cardiovasc Care. 2017 Oct;6(7):610–22.
55.
Bugiardini R, Ricci B, Cenko E, Vasiljevic Z, Kedev S, Davidovic G, et al. Delayed Care and Mortality Among Women and Men With Myocardial Infarction. J Am Heart Assoc. 2017 Aug;6(8).
56.
Buja A, Canavese D, Furlan P, Lago L, Saia M, Baldo V. Are hospital process quality indicators influenced by socio-demographic health determinants. Eur J Public Health. 2015 Oct;25(5):759–65.
57.
Buleshov MA, Buleshov DM, Yermakhanova ZA, Dauitov TB, Alipbekova SN, Tuktibayeva SA, et al. The choice of treatment for myocardial infarction based on individual cardiovascular risk and symptoms of coronary heart disease. Electronic Journal of General Medicine [Internet]. 2019;16(6). Available from:

https://www.embase.com/search/results?subaction=viewrecord&id=L2003571792&from=export
http://dx.doi.org/10.29333/ejgm/115860
58.
Burgess S, Juergens CP, Yang W, Shugman IM, Idris H, Nguyen T, et al. Sex Differences in Outcome and Prescribing Practice in ST-elevation MI Patients with Multivessel Disease and Incomplete Revascularisation. European Cardiology Review [Internet]. 2023;18. Available from:
https://www.embase.com/search/results?subaction=viewrecord&id=L2025011113&from=export
http://dx.doi.org/10.15420/ecr.2022.39
59.
Burlacu A, Tinica G, Nedelciuc I, Simion P, Artene B, Covic A. Strategies to Lower In-Hospital Mortality in STEMI Patients with Primary PCI: Analysing Two Years Data from a High-Volume Interventional Centre. J Interv Cardiol. 2019;2019:3402081.
60.
Bush M, Glickman LT, Fern, ez AR, Garvey JL, Glickman SW. Variation in the use of 12-lead electrocardiography for patients with chest pain by emergency medical services in North Carolina. J Am Heart Assoc. 2013 Aug;2(4):e000289.
61.
Cacciani L, Agabiti N, Bargagli AM, Davoli M. Access to percutaneous transluminal coronary angioplasty and 30-day mortality in patients with incident STEMI: Differentials by educational level and gender over 11 years. PLoS One. 2017;12(4):e0175038.
62.
Cader FA, Kabir CS, Nasrin S, Amin MN, Haq MM. Predictors of In-hospital Mortality in Patients Presenting with Acute Coronary Syndromes in a Tertiary Cardiac Center in Dhaka, Bangladesh. Mymensingh Med J. 2022 Oct;31(4):1057–67.
63.
Cai X, Zhou J, Li W, Cheng L, Yuan Z, Xiao Y. Potential Influential Factors of In-Hospital Myocardial Reinfarction in ST-Segment Elevation Myocardial Infarction (STEMI) Patients: Finding from the Improving Care for Cardiovascular Disease in China- (CCC-) Acute Coronary Syndrome (ACS) Project. Oxid Med Cell Longev. 2021;2021:9977312.
64.
Calé R, Pereira H, Pereira E, Vitorino S, de Mello S. Time to reperfusion in high-risk patients with myocardial infarction undergoing primary percutaneous coronary intervention. Rev Port Cardiol (Engl Ed). 2019 Sep;38(9):637–46.
65.
Câlmâc L, Bătăilă V, Ricci B, Vasiljevic Z, Kedev S, Gustiene O, et al. Factors associated with use of percutaneous coronary intervention among elderly patients presenting with ST segment elevation acute myocardial infarction (STEMI): Results from the ISACS-TC registry. Int J Cardiol. 2016 Aug;217:S21-6.
66.
Campi Jr TR, George S, Villacís D, Ward-Peterson M, Barengo NC, Zevallos JC. Effect of charted mental illness on reperfusion therapy in hospitalized patients with an acute myocardial infarction in Florida. Medicine (Baltimore). 2017 Aug;96(34):e7788.
67.
Cantor WJ, Ko DT, Natarajan MK, Džavík V, Wijeysundera HC, Wang JT, et al. Reperfusion Times for Radial Versus Femoral Access in Patients With ST-Elevation Myocardial Infarction Undergoing Primary Percutaneous Coronary Intervention: Observations From the Cardiac Care Network Provincial Primary PCI Registry. Circ Cardiovasc Interv. 2015 May;8(5).
68.
Carol Ruiz A, Masip Utset J, Ariza-Solé A, Gómez-Hospital JA, Carrillo X, Tizón H, et al. Predictors of primary percutaneous coronary intervention delay in cases of myocardial infarction diagnosed in hospitals without hemodynamic support systems. Emergencias. 2021 Jun;33(3):187–94.
69.

Casella G, Di Pasquale G, Oltrona Visconti L, Pallotti MG, Lucci D, Caldarola P, et al. Management of patients with acute coronary syndromes in real-world practice in Italy: an outcome research study focused on the use of ANTithRombotic Agents: the MANTRA registry. Eur Heart J Acute Cardiovasc Care. 2013 Mar;2(1):27–34.
70.
Castro PPN, Castro MAN, Nascimento GA, Moura I, Pena JLB. Predictors of Hospital Mortality Based on Primary Angioplasty Treatment: A Multicenter Case-Control Study. Arq Bras Cardiol. 2022 Sep;119(3):448–57.
71.
Cenko E, Ricci B, Kedev S, Vasiljevic Z, Dorobantu M, Gustiene O, et al. Reperfusion therapy for ST-elevation acute myocardial infarction in Eastern Europe: The ISACS-TC registry. European Heart Journal - Quality of Care and Clinical Outcomes. 2016;2(1):45–51.
72.
Ch, rasekhar J, Baber U, Sartori S, Faggioni M, Aquino M, et al. Sex-related differences in outcomes among men and women under 55 years of age with acute coronary syndrome undergoing percutaneous coronary intervention: Results from the PROMETHEUS study. Catheter Cardiovasc Interv. 2017 Mar;89(4):629–37.
73.
Cha JJ, Bae S, Park DW, Park JH, Hong SJ, Park SM, et al. Clinical Outcomes in Patients With Delayed Hospitalization for Non-ST-Segment Elevation Myocardial Infarction. J Am Coll Cardiol. 2022 Feb;79(4):311–23.
74.
Chakraborty S, Amgai B, B, yopadhyay D, Patel N, Hajra A, et al. Acute myocardial infarction in the young with diabetes mellitus- national inpatient sample study with sex-based difference in outcomes. Int J Cardiol. 2021 Mar;326:35–41.
75.
Chamtouri I, Souissi R, Amdouni N, Jomaa W, Abdallah W, Hamda KB, et al. ST-segment Elevation Myocardial Infarction in North African Women: Results From a Twenty-year Experience. Journal of the Saudi Heart Association. 2022;34(3):166–74.
76.
Chang SS, Lin SY, Lai JN, Chen KW, Lu CR, Chang KC, et al. Sex differences in long-term cardiovascular outcomes among patients with acute myocardial infarction: A population-based retrospective cohort study. Int J Clin Pract. 2021 May;75(5):e14066.
77.
Chen SI, Wang Y, Dreyer R, Strait KM, Spatz ES, Xu X, et al. Insurance and Prehospital Delay in Patients ≤55 Years With Acute Myocardial Infarction. Am J Cardiol. 2015 Dec;116(12):1827–32.
78.
Chen S, Li J, Qiu M, Ma S, Jiang Z, Na K, et al. Predictors and long-term outcomes of in-hospital switching from clopidogrel to ticagrelor among patients with acute coronary syndrome undergoing percutaneous coronary intervention. Catheter Cardiovasc Interv. 2022 May;99:1424–31.
79.
Chernomordik F, Sabbag A, Tzur B, Kopel E, Goldkorn R, Matetzky S, et al. Cardiac rehabilitation following an acute coronary syndrome: Trends in referral, predictors and mortality outcome in a multicenter national registry between years 2006-2013: Report from the Working Group on Cardiac Rehabilitation, the Israeli Heart Society. Eur J Prev Cardiol. 2017 Jan;24(2):123–32.
80.
Chou LP, Zhao P, Kao C, Chen YH, Jong GP. Women were noninferior to men in cardiovascular outcomes among patients with ST-segment elevation myocardial infarction treated with primary percutaneous coronary intervention from Taiwan acute coronary syndrome full-spectrum registry. Medicine (Baltimore). 2018 Oct;97(43):e12998.
81.
Cicek G, Yıldırım E. CHA2DS2-VASc score predicts contrast-induced nephropathy in patients with ST-segment elevation myocardial infarction, who have undergone primary percutaneous coronary intervention. Kardiol Pol. 2018;76(1):91–8.
82.
Colón Arias FA, Gutiérrez Martínez A, Pereyra Bencosme K, Mercado Domínguez A, Gutiérrez Lora F, Almonte Casado M, et al. Diferencia de presentación del síndrome coronario agudo por género en pacientes llevados al

laboratorio de cateterismo en población dominicana: un estudio retrospectivo. Cienc Salud (St Domingo). 2021 Feb;5(2):[69-76].
83.
Corrada E, Ferrante G, Mazzali C, Barbieri P, Merlino L, Merlini P, et al. Eleven-year trends in gender differences of treatments and mortality in ST-elevation acute myocardial infarction in northern Italy, 2000 to 2010. Am J Cardiol. 2014 Aug;114(3):336–41.
84.
Coventry LL, Bremner AP, Jacobs IG, Finn J. Myocardial infarction: sex differences in symptoms reported to emergency dispatch. Prehosp Emerg Care. 2013 Apr;17(2):193–202.
85.
Cui ER, Beja-Glasser A, Fern, ez AR, Grover JM, Mann NC, et al. Emergency Medical Services Time Intervals for Acute Chest Pain in the United States, 2015-2016. Prehosp Emerg Care. 2020 Jul;24(4):557–65.
86.
D'Ascenzo F, Grosso A, Abu-Assi E, Kinnaird T, Ariza-Solé A, Manzano-Fernández S, et al. Incidence and predictors of bleeding in ACS patients treated with PCI and prasugrel or ticagrelor: An analysis from the RENAMI registry. Int J Cardiol. 2018 Dec;273:29–33.
87.
D'Onofrio G, Safdar B, Lichtman JH, Strait KM, Dreyer RP, Geda M, et al. Sex differences in reperfusion in young patients with ST-segment-elevation myocardial infarction: results from the VIRGO study. Circulation. 2015 Apr;131(15):1324–32.
88.
Dafaalla M, Rashid M, Van Spall HGC, Mehta L, Parwani P, Sharma G, et al. Women Are Less Likely to Survive AMI Presenting With Out-of-Hospital Cardiac Arrest: A Nationwide Study. Mayo Clin Proc. 2022 Sep;97(9):1608–18.
89.
Dang D, Kuhn L, Fooladi E, Ky V, Cheung K, Rashid H, et al. Predictors of Radial to Femoral Artery Access Crossover During Primary Percutaneous Coronary Intervention for ST-Elevation Myocardial Infarction. Heart Lung Circ. 2022 Jul;31(7):985–92.
90.
Dawson LP, Nehme E, Nehme Z, Davis E, Bloom J, Cox S, et al. Sex Differences in Epidemiology, Care, and Outcomes in Patients With Acute Chest Pain. J Am Coll Cardiol. 2023 Mar;81(10):933–45.
91.
De Luca G, Gibson MC, Hof AW, Cutlip D, Zeymer U, Noc M, et al. Impact of time-to-treatment on myocardial perfusion after primary percutaneous coronary intervention with Gp IIb-IIIa inhibitors. J Cardiovasc Med (Hagerstown). 2013 Nov;14(11):815–20.
92.
De Luca L, Marini M, Gonzini L, Boccanelli A, Casella G, Chiarella F, et al. Contemporary Trends and Age-Specific Sex Differences in Management and Outcome for Patients With ST-Segment Elevation Myocardial Infarction. J Am Heart Assoc. 2016 Nov;5(12).
93.
de Miguel-Yanes JM, Jiménez-García R, Hern, ez-Barrera V, de Miguel-Díez J, Muñoz-Rivas N, et al. Sex differences in the incidence and outcomes of acute myocardial infarction in spain, 2016–2018: A matched-pair analysis. Journal of Clinical Medicine [Internet]. 2021;10(8). Available from:
https://www.embase.com/search/results?subaction=viewrecord&id=L2006985760&from=export
http://dx.doi.org/10.3390/jcm10081795
94.
DeFilippis EM, Collins BL, Singh A, Biery DW, Fatima A, Qamar A, et al. Women who experience a myocardial infarction at a young age have worse outcomes compared with men: the Mass General Brigham YOUNG-MI registry. Eur Heart J. 2020 Nov;41(42):4127–37.
95.
Denlinger LN, Keeley EC. Medication Administration Delays in Non-ST Elevation Myocardial Infarction: Analysis of 1002 Patients Admitted to an Academic Medical Center. Crit Pathw Cardiol. 2018 Jun;17(2):73–6.
96.

Dharma S, Dakota I, Andriantoro H, Firdaus I, Rahma S, Budi Siswanto B. Association of gender with clinical outcomes of patients with acute ST-segment elevation myocardial infarction presenting with acute heart failure. Coron Artery Dis. 2021 Jan;32(1):17–24.
97.
Dharma S, Dakota I, Andriantoro H, Firdaus I, An, ira CP, et al. Interhospital Transfer versus Direct Admission in Patients with Acute ST-Segment Elevation Myocardial Infarction. International Journal of Angiology. 2023;32(2):121–7.
98.
Díez-Villanueva P, García-Acuña JM, Raposeiras-Roubin S, Barrabés JA, Cordero A, Martínez-Sellés M, et al. Prognosis impact of diabetes in elderly women and men with non-st elevation acute coronary syndrome. Journal of Clinical Medicine [Internet]. 2021;10(19). Available from: https://www.embase.com/search/results?subaction=viewrecord&id=L2013895206&from=export http://dx.doi.org/10.3390/jcm10194403
99.
Donataccio MP, Puymirat E, Parapid B, Steg PG, Eltchaninoff H, Weber S, et al. In-hospital outcomes and long-term mortality according to sex and management strategy in acute myocardial infarction. Insights from the French ST-elevation and non-ST-elevation Myocardial Infarction (FAST-MI) 2005 Registry. International Journal of Cardiology. 2015;201:265–70.
100.
Du X, Spatz ES, Dreyer RP, Hu S, Wu C, Li X, et al. Sex Differences in Clinical Profiles and Quality of Care Among Patients With ST-Segment Elevation Myocardial Infarction From 2001 to 2011: Insights From the China Patient-Centered Evaluative Assessment of Cardiac Events (PEACE)-Retrospective Study. J Am Heart Assoc. 2016 Feb;5(2).
101.
El-Ahmadi A, Abassi MS, Andersson HB, Engstrøm T, Clemmensen P, Helqvist S, et al. Acute kidney injury - A frequent and serious complication after primary percutaneous coronary intervention in patients with ST-segment elevation myocardial infarction. PLoS One. 2019;14(12):e0226625.
102.
El-Menyar A, Ahmed E, Albinali H, Al-Thani H, Gehani A, Singh R, et al. Mortality trends in women and men presenting with acute coronary syndrome: insights from a 20-year registry. PLoS One. 2013;8(7):e70066.
103.
Elbaz-Greener G, Rozen G, Kusniec F, Marai I, Ghanim D, Carasso S, et al. Trends in Utilization and Safety of In-Hospital Coronary Artery Bypass Grafting During a Non-ST-Segment Elevation Myocardial Infarction. Am J Cardiol. 2020 Nov;134:32–40.
104.
Elgendy IY, Wegermann ZK, Li S, Mahtta D, Grau-Sepulveda M, Smilowitz NR, et al. Sex Differences in Management and Outcomes of Acute Myocardial Infarction Patients Presenting With Cardiogenic Shock. JACC Cardiovasc Interv. 2022 Mar;15(6):642–52.
105.
Erne P, Radovanovic D, Schoenenberger AW, Bertel O, Kaeslin T, Essig M, et al. Impact of hypertension on the outcome of patients admitted with acute coronary syndrome. J Hypertens. 2015 Apr;33(4):860–7.
106.
Erne P, Radovanovic D, Seifert B, Bertel O, Urban P. Outcome of patients admitted with acute coronary syndrome on palliative treatment: insights from the nationwide AMIS Plus Registry 1997-2014. BMJ Open. 2015 Mar;5(3):e006218.
107.
Ezekowitz JA, Savu A, Welsh RC, McAlister FA, Goodman SG, Kaul P. Is There a Sex Gap in Surviving an Acute Coronary Syndrome or Subsequent Development of Heart Failure? Circulation. 2020 Dec;142(23):2231–9.
108.
Fan PC, Chen TH, Lee CC, Tsai TY, Chen YC, Chang CH. ADVANCIS Score Predicts Acute Kidney Injury After Percutaneous Coronary Intervention for Acute Coronary Syndrome. Int J Med Sci. 2018;15(5):528–35.
109.

Fernández-Rodríguez D, Regueiro A, Cevallos J, Bosch X, Freixa X, Trilla M, et al. Gender gap in medical care in ST segment elevation myocardial infarction networks: Findings from the Catalan network Codi Infart. Med Intensiva. 2017 Mar;41(2):70–7.
110.
Flores-Umanzor E, Cepas-Guillén P, Freixa X, Regueiro A, Tizón-Marcos H, Brugaletta S, et al. Clinical profile and prognosis of young patients with ST-elevation myocardial infarction managed by the emergency-intervention Codi IAM network. Revista Espanola de Cardiologia [Internet]. 2023; Available from: https://www.embase.com/search/results?subaction=viewrecord&id=L2025070061&from=export http://dx.doi.org/10.1016/j.recesp.2023.03.009
111.
Foster-Witassek F, Rickli H, Roffi M, Pedrazzini G, Eberli F, Fassa A, et al. Reducing gap in pre-hospital delay between women and men presenting with ST-elevation myocardial infarction. Eur J Prev Cardiol. 2023 Aug;30(11):1056–62.
112.
Freisinger E, Sehner S, Malyar NM, Suling A, Reinecke H, Wegscheider K. Nationwide Routine-Data Analysis of Sex Differences in Outcome of Acute Myocardial Infarction. Clin Cardiol. 2018 Aug;41(8):1013–21.
113.
Frydman S, Freund O, Banai A, Zornitzki L, Banai S, Shacham Y. Relation of Gender to the Occurrence of AKI in STEMI Patients. Journal of Clinical Medicine [Internet]. 2022;11(21). Available from: https://www.embase.com/search/results?subaction=viewrecord&id=L2020032469&from=export http://dx.doi.org/10.3390/jcm11216565
114.
Gao S, Ma W, Huang S, Lin X, Yu M. Sex-Specific Clinical Characteristics and Long-Term Outcomes in Patients With Myocardial Infarction With Non-obstructive Coronary Arteries. Front Cardiovasc Med. 2021;8:670401.
115.
García-García C, Molina L, Subirana I, Sala J, Bruguera J, Arós F, et al. Sex-based differences in clinical features, management, and 28-day and 7-year prognosis of first acute myocardial infarction. RESCATE II study. Rev Esp Cardiol (Engl Ed). 2014 Jan;67(1):28–35.
116.
Gardarsdottir HR, Sigurdsson MI, Andersen K, Gudmundsdottir IJ. Long-term survival of Icelandic women following acute myocardial infarction. Scand Cardiovasc J. 2022 Dec;56(1):114–20.
117.
Gausia K, Katzenellenbogen JM, Sanfilippo FM, Knuiman MW, Thompson PL, Hobbs MS, et al. Evidence-based prescribing of drugs for secondary prevention of acute coronary syndrome in Aboriginal and non-Aboriginal patients admitted to Western Australian hospitals. Intern Med J. 2014 Apr;44(4):353–61.
118.
Gauthier V, Montaye M, Ferrières J, Kai SHY, Biasch K, Moitry M, et al. Sex differences in time trends in acute coronary syndrome management and in 12-month lethality: Data from the French MONICA registries. Int J Cardiol. 2022 Aug;361:103–8.
119.
Gevaert SA, De Bacquer D, Evrard P, Renard M, Beauloye C, Coussement P, et al. Renal dysfunction in STEMI-patients undergoing primary angioplasty: higher prevalence but equal prognostic impact in female patients; an observational cohort study from the Belgian STEMI registry. BMC Nephrol. 2013 Mar;14:62.
120.
Gevaert SA, De Bacquer D, Evrard P, Convens C, Dubois P, Bol, et al. Gender, TIMI risk score and in-hospital mortality in STEMI patients undergoing primary PCI: results from the Belgian STEMI registry. EuroIntervention. 2014 Jan;9(9):1095–101.
121.
Ghadri JR, Sarcon A, Jaguszewski M, Diekmann J, Bataiosu RD, Hellermann J, et al. Gender disparities in acute coronary syndrome: a closing gap in the short-term outcome. J Cardiovasc Med (Hagerstown). 2015 May;16(5):355–62.
122.

Ghaffari S, Pourafkari L, Tajlil A, Bahmani-Oskoui R, Nader ND. Is female gender associated with worse outcome after ST elevation myocardial infarction? Indian Heart J. 2017 Apr;69:S28-s33.
123.
Gnavi R, Rusciani R, Dalmasso M, Giammaria M, Anselmino M, Roggeri DP, et al. Gender, socioeconomic position, revascularization procedures and mortality in patients presenting with STEMI and NSTEMI in the era of primary PCI. Differences or inequities? International Journal of Cardiology [Internet]. 2014; Available from: https://www.embase.com/search/results?subaction=viewrecord&id=L53313403&from=export http://dx.doi.org/10.1016/j.ijcard.2014.07.107
124.
Gong IY, Goodman SG, Brieger D, Gale CP, Chew DP, Welsh RC, et al. GRACE risk score: Sex-based validity of in-hospital mortality prediction in Canadian patients with acute coronary syndrome. Int J Cardiol. 2017 Oct;244:24–9.
125.
Greenwood BN, Carnahan S, Huang L. Patient-physician gender concordance and increased mortality among female heart attack patients. Proc Natl Acad Sci U S A. 2018 Aug;115(34):8569–74.
126.
Gudnadottir GS, Andersen K, Thrainsdottir IS, James SK, Lagerqvist B, Gudnason T. Gender differences in coronary angiography, subsequent interventions, and outcomes among patients with acute coronary syndromes. Am Heart J. 2017 Sep;191:65–74.
127.
Guo W, Du X, Gao Y, Hu S, Lu Y, Dreyer RP, et al. Sex Differences in Characteristics, Treatments, and Outcomes Among Patients Hospitalized for Non-ST-Segment-Elevation Myocardial Infarction in China: 2006 to 2015. Circ Cardiovasc Qual Outcomes. 2022 Jun;15(6):e008535.
128.
Gupta A, Barrabes JA, Strait K, Bueno H, Porta-Sánchez A, Acosta-Vélez JG, et al. Sex Differences in Timeliness of Reperfusion in Young Patients With ST-Segment-Elevation Myocardial Infarction by Initial Electrocardiographic Characteristics. J Am Heart Assoc. 2018 Mar;7(6).
129.
Gupta A, Chui P, Zhou S, Spertus JA, Geda M, Lorenze N, et al. Frequency and Effects of Excess Dosing of Anticoagulants in Patients ≤55 Years With Acute Myocardial Infarction Who Underwent Percutaneous Coronary Intervention (from the VIRGO Study). Am J Cardiol. 2015 Jul;116(1):1–7.
130.
Gupta T, Kolte D, Khera S, Agarwal N, Villablanca PA, Goel K, et al. Contemporary Sex-Based Differences by Age in Presenting Characteristics, Use of an Early Invasive Strategy, and Inhospital Mortality in Patients With Non-ST-Segment-Elevation Myocardial Infarction in the United States. Circ Cardiovasc Interv. 2018 Jan;11(1):e005735.
131.
Haghighat L, Reinhardt SW, Saly DL, Lu D, Matsouaka RA, Wang TY, et al. Comfort Measures Only in Myocardial Infarction: Prevalence of This Status, Change Over Time, and Predictors From a Nationwide Study. Circ Cardiovasc Qual Outcomes. 2022 Jan;15(1):e007610.
132.
Hannan EL, Wu Y, Tamis-Holl, J., Jacobs AK, Berger PB, et al. Sex differences in the treatment and outcomes of patients hospitalized with ST-elevation myocardial infarction. Catheter Cardiovasc Interv. 2020 Feb;95(2):196–204.
133.
Hansen KW, Soerensen R, Madsen M, Madsen JK, Jensen JS, von Kappelgaard LM, et al. Developments in the invasive diagnostic-therapeutic cascade of women and men with acute coronary syndromes from 2005 to 2011: a nationwide cohort study. BMJ Open. 2015 Jun;5(6):e007785.
134.
Hao K, Takahashi J, Ito K, Miyata S, Nihei T, Nishimiya K, et al. Clinical Characteristics of Patients With Acute Myocardial Infarction Who Did Not Undergo Primary Percutaneous Coronary Intervention- Report From the MIYAGI-AMI Registry Study. Circ J. 2015;79(9):2009–16.
135.

Hao Y, Liu J, Liu J, Yang N, Smith Jr SC, Huo Y, et al. Sex Differences in In-Hospital Management and Outcomes of Patients With Acute Coronary Syndrome. Circulation. 2019 Apr;139(15):1776–85.
136.
Hao Z, Ma J, Dai J, Shao Q, Shen L, He B, et al. A real-world analysis of cardiac rupture on incidence, risk factors and in-hospital outcomes in 4190 ST-elevation myocardial infarction patients from 2004 to 2015. Coron Artery Dis. 2020 Aug;31(5):424–9.
137.
Hassani NS, Mozafarybazargany M, Pirdehghan R, Sepahv, i R, Khodaprast Z, et al. The outcome of ST-elevation myocardial infarction by sex: a retrospective cohort study. Future Cardiol. 2023 Jan;19(1):19–27.
138.
Heer T, Hochadel M, Schmidt K, Mehilli J, Zahn R, Kuck KH, et al. Gender differences in therapeutic recommendation after diagnostic coronary angiography: insights from the Coronary Angiography and PCI Registry of the German Society of Cardiology. Clin Res Cardiol. 2015 Jun;104(6):507–17.
139.
Heer T, Hochadel M, Schmidt K, Mehilli J, Zahn R, Kuck KH, et al. Sex Differences in Percutaneous Coronary Intervention-Insights From the Coronary Angiography and PCI Registry of the German Society of Cardiology. J Am Heart Assoc. 2017 Mar;6(3).
140.
Hellgren T, Blöndal M, Jortveit J, Ferenci T, Faxén J, Lewinter C, et al. Sex-related differences in the management and outcomes of patients hospitalized with ST-elevation myocardial infarction: a comparison within four European myocardial infarction registries. Eur Heart J Open. 2022 Jul;2(4):oeac042.
141.
Her AY, Shin ES, Kim YH, Garg S, Jeong MH. The contribution of gender and age on early and late mortality following ST-segment elevation myocardial infarction: results from the Korean Acute Myocardial Infarction National Registry with Registries. J Geriatr Cardiol. 2018 Mar;15(3):205–14.
142.
Hernandez-Suarez DF, Osterman-Pla AD, Carrasquillo O, Aranda J, Baez S, Lopez M, et al. Epidemiological Profile of Hispanics Admitted With Acute Myocardial Infarction in Puerto Rico: The Experience of 2007, 2009 and 2011. J Clin Med Res. 2017 Jun;9(6):528–33.
143.
Herscovici R, Mirocha J, Salomon J, Merz NB, Cercek B, Goldfarb M. Sex differences in crude mortality rates and predictive value of intensive care unit-based scores when applied to the cardiac intensive care unit. Eur Heart J Acute Cardiovasc Care. 2020 Dec;9(8):966–74.
144.
Hersi A, Al-Habib K, Al-Faleh H, Al-Nemer K, Alsaif S, Taraben A, et al. Gender inequality in the clinical outcomes of equally treated acute coronary syndrome patients in Saudi Arabia. Ann Saudi Med. 2013 Jul;33(4):339–46.
145.
Hess CN, McCoy LA, Duggirala HJ, Tavris DR, O'Callaghan K, Douglas PS, et al. Sex-based differences in outcomes after percutaneous coronary intervention for acute myocardial infarction: a report from TRANSLATE-ACS. J Am Heart Assoc. 2014 Feb;3(1):e000523.
146.
Hickson RP, Kucharska-Newton AM, Rodgers JE, Sleath BL, Fang G. Disparities by sex in P2Y(12) inhibitor therapy duration, or differences in the balance of ischaemic-benefit and bleeding-risk clinical outcomes in older women versus comparable men following acute myocardial infarction? A P2Y(12) inhibitor new user retrospective cohort analysis of US Medicare claims data. BMJ Open. 2021 Dec;11(12):e050236.
147.
Hillinger P, Twerenbold R, Wildi K, Rubini Gimenez M, Jaeger C, Boeddinghaus J, et al. Gender-specific uncertainties in the diagnosis of acute coronary syndrome. Clin Res Cardiol. 2017 Jan;106(1):28–37.
148.
Hinohara TT, Al-Khalidi HR, Fordyce CB, Gu X, Sherwood MW, Roettig ML, et al. Impact of Regional Systems of Care on Disparities in Care Among Female and Black Patients Presenting With ST-Segment-Elevation Myocardial Infarction. J Am Heart Assoc. 2017 Oct;6(10).

149.
Hoedemaker NPG, de Winter RJ, Hof AV, Kolkman E, Damman P. Optimal Medical Therapy Prescription in Patients with Acute Coronary Syndrome in the Netherlands: A Multicenter Pilot Registry. Am J Cardiovasc Drugs. 2021 Mar;21(2):219–29.
150.
Holm A, Sederholm Lawesson S, Swahn E, Alfredsson J. Editor's Choice- Gender difference in prognostic impact of in-hospital bleeding after myocardial infarction - data from the SWEDEHEART registry. Eur Heart J Acute Cardiovasc Care. 2016 Oct;5(6):463–72.
151.
Honda S, Asaumi Y, Yamane T, Nagai T, Miyagi T, Noguchi T, et al. Trends in the clinical and pathological characteristics of cardiac rupture in patients with acute myocardial infarction over 35 years. Journal of the American Heart Association [Internet]. 2014;3(5). Available from: https://www.embase.com/search/results?subaction=viewrecord&id=L603523274&from=export http://dx.doi.org/10.1161/JAHA.114.000984
152.
Hong JS, Kang HC. Sex Differences in the Treatment and Outcome of Korean Patients With Acute Myocardial Infarction Using the Korean National Health Insurance Claims Database. Medicine (Baltimore). 2015 Sep;94(35):e1401.
153.
Hsu B, Carcel C, Wang X, Peters SAE, Randall DA, Havard A, et al. Sex differences in emergency medical services management of patients with myocardial infarction: analysis of routinely collected data for over 110,000 patients. Am Heart J. 2021 Nov;241:87–91.
154.
Hu DQ, Hao YC, Liu J, Yang N, Yang YQ, Sun ZQ, et al. Pre-hospital delay in patients with acute myocardial infarction in China: findings from the Improving Care for Cardiovascular Disease in China-Acute Coronary Syndrome (CCC-ACS) project. Journal of Geriatric Cardiology. 2022;19(4):276–83.
155.
Huang N, Chou YJ, Hu HY, Lee CH. Gender disparities in AMI management and outcomes among health professionals, their relatives, and non-health professionals in Taiwan from 1997 to 2007. Soc Sci Med. 2013 Jan;77:70–4.
156.
Huber E, Le Pogam MA, Clair C. Sex related inequalities in the management and prognosis of acute coronary syndrome in Switzerland: cross sectional study. BMJ Med. 2022;1(1):e000300.
157.
Huded CP, Johnson M, Kravitz K, Menon V, Abdallah M, Gullett TC, et al. 4-Step Protocol for Disparities in STEMI Care and Outcomes in Women. J Am Coll Cardiol. 2018 May;71(19):2122–32.
158.
Huded CP, Kumar A, Kassis N, Johnson MJ, Kravitz K, Brown A, et al. Five years of a comprehensive ST-elevation myocardial infarction protocol and its association with sex disparities. Eur Heart J Open. 2021 Nov;1(3):oeab011.
159.
Humphries KH, Gao M, Lee MK, Izadnegahdar M, Holmes DT, Scheuermeyer FX, et al. Sex Differences in Cardiac Troponin Testing in Patients Presenting to the Emergency Department with Chest Pain. J Womens Health (Larchmt). 2018 Nov;27(11):1327–34.
160.
Ijaz SH, Minhas AMK, Jain V, Rifai MA, Sharma G, Mehta A, et al. Characteristics and outcomes in acute myocardial infarction hospitalizations among the older population (age ≥80 years) in the United States, 2004-2018. Arch Gerontol Geriatr. 2023 Aug;111:104930.
161.
Inan D, Yumurtas AC, Simsek B, Palice A, Efendioglu EM, Yuksel G, et al. Performance of the Academic Research Consortium High Bleeding Risk Criteria in Patients With ST-Segment Elevation Myocardial Infarction: A Single Center Study. Angiology. 2022 Oct;33197221135739.
162.

Isorni MA, Blanchard D, Teixeira N, le Breton H, Renault N, Gilard M, et al. Impact of gender on use of revascularization in acute coronary syndromes: the national observational study of diagnostic and interventional cardiac catheterization (ONACI). Catheter Cardiovasc Interv. 2015 Aug;86(2):E58-65.
163.
Iyanoye A, Moreyra AE, Swerdel JN, Gandhi SK, Cabrera J, Cosgrove NM, et al. Gender disparity in the use of drug-eluting stents during percutaneous coronary intervention for acute myocardial infarction. Catheter Cardiovasc Interv. 2015 Aug;86(2):221–8.
164.
Izadnegahdar M, Singer J, Lee MK, Gao M, Thompson CR, Kopec J, et al. Do younger women fare worse? Sex differences in acute myocardial infarction hospitalization and early mortality rates over ten years. J Womens Health (Larchmt). 2014 Jan;23(1):10–7.
165.
Jang SJ, Kim LK, Sobti NK, Yeo I, Cheung JW, Feldman DN, et al. Mortality of patients with ST-segment-elevation myocardial infarction without standard modifiable risk factors among patients without known coronary artery disease: Age-stratified and sex-related analysis from nationwide readmissions database 2010-2014. Am J Prev Cardiol. 2023 Jun;14:100474.
166.
Janjani P, Motevasel S, Salimi Y, Siabani S, Asadmobini A, Salehi N. Gender and Smoking-Related Survival Differences in Patients with ST-Elevation Myocardial Infarction. International Cardiovascular Research Journal. 2022;16(3):118–22.
167.
Jankowski P, Kozieł P, Setny M, Paniczko M, Haberka M, Banach M, et al. Dyslipidemia management in patients with coronary artery disease. Data from the POLASPIRE survey. Journal of Clinical Medicine [Internet]. 2021;10(16). Available from: https://www.embase.com/search/results?subaction=viewrecord&id=L2013494430&from=export http://dx.doi.org/10.3390/jcm10163711
168.
Jánosi A, Ferenci T, Ofner P, Lupkovics G, Becker D, Faluközy J, et al. Does Gender Have Prognostic Value Among Patients with Myocardial Infarction? Analysis of the Data from the Hungarian Myocardial Infarction Registry. J Womens Health (Larchmt). 2018 Dec;27(12):1491–8.
169.
Jarrah MI, Hammoudeh AJ, Al-Natour DB, Khader YS, Tabbalat RA, Alhaddad IA, et al. Gender differences in risk profile and outcome of Middle Eastern patients undergoing percutaneous coronary intervention. Saudi Med J. 2017 Feb;38(2):149–55.
170.
Johnston N, Bornefalk-Hermansson A, Schenck-Gustafsson K, Held C, Goodman SG, Yan AT, et al. Do clinical factors explain persistent sex disparities in the use of acute reperfusion therapy in STEMI in Sweden and Canada? Eur Heart J Acute Cardiovasc Care. 2013 Dec;2(4):350–8.
171.
Johnston N, Jönelid B, Christersson C, Kero T, Renlund H, Schenck-Gustafsson K, et al. Effect of Gender on Patients With ST-Elevation and Non-ST-Elevation Myocardial Infarction Without Obstructive Coronary Artery Disease. Am J Cardiol. 2015 Jun;115(12):1661–6.
172.
Jortveit J, Govatsmark RE, Langørgen J, Hole T, Mannsverk J, Olsen S, et al. Gender differences in the assessment and treatment of myocardial infarction. Tidsskr Nor Laegeforen. 2016 Aug;136(14):1215–22.
173.
Josiah A, Farshid A. Gender is Not a Predictor of Mortality or Major Adverse Cardiovascular Events in Patients Undergoing Percutaneous Coronary Intervention for Acute Coronary Syndromes. Heart Lung Circ. 2019 May;28(5):727–34.
174.

Juliard JM, Golmard JL, Himbert D, Feldman LJ, Delorme L, Ducrocq G, et al. Comparison of hospital mortality during ST-segment elevation myocardial infarction in the era of reperfusion therapy in women versus men and in older versus younger patients. Am J Cardiol. 2013 Jun;111(12):1708–13.
175.
Kanic V, Vollrath M, Kompara G, Suran D, Hojs R. Women and acute kidney injury in myocardial infarction. J Nephrol. 2018 Oct;31(5):713–9.
176.
Kanic V, Vollrath M, Naji FH, Sinkovic A. Gender Related Survival Differences in ST-Elevation Myocardial Infarction Patients Treated with Primary PCI. Int J Med Sci. 2016;13(6):440–4.
177.
Kaul P, Tanguay JF, Newby LK, Hochman JS, Westerhout CM, Califf RM, et al. Association between bleeding and mortality among women and men with high-risk acute coronary syndromes: insights from the Early versus Delayed, Provisional Eptifibatide in Acute Coronary Syndromes (EARLY ACS) trial. Am Heart J. 2013 Oct;166(4):723–8.
178.
Kerola AM, Palomäki A, Rautava P, Nuotio M, Kytö V. Sex Differences in Cardiovascular Outcomes of Older Adults After Myocardial Infarction. J Am Heart Assoc. 2021 Dec;10(23):e022883.
179.
Kerola AM, Palomäki A, Rautava P, Kytö V. Less revascularization in young women but impaired long-term outcomes in young men after myocardial infarction. Eur J Prev Cardiol. 2022 Aug;29(10):1437–45.
180.
Khaled S, Almalki M, Shalaby G, Niazi AK, Ahmed S, Alsilami A, et al. Epidemiological variation of acute myocardial infarction relevant to in-hospital outcomes-tertiary center experience-saudi arabia. Journal of the Saudi Heart Association. 2020;32(3):340–9.
181.
Khaled S, Jaha N, Shalaby G. Clinical Characteristics and Short-Term Outcomes of Patients Presenting with Acute Myocardial Infarction having Multi-vessel disease - A Single Middle- eastern Tertiary-Care Center Experience. Indian Heart J. 2022 Jan;74(1):28–33.
182.
Khan E, Brieger D, Amerena J, Atherton JJ, Chew DP, Farshid A, et al. Differences in management and outcomes for men and women with ST-elevation myocardial infarction. Med J Aust. 2018 Aug;209(3):118–23.
183.
Khan MZ, Munir MB, Khan MU, Osman M, Agrawal P, Syed M, et al. Trends, Outcomes, and Predictors of Revascularization in Cardiogenic Shock. Am J Cardiol. 2020 Feb;125(3):328–35.
184.
Khan NA, Daskalopoulou SS, Karp I, Eisenberg MJ, Pelletier R, Tsadok MA, et al. Sex differences in prodromal symptoms in acute coronary syndrome in patients aged 55 years or younger. Heart. 2017 Jun;103(11):863–9.
185.
Khera S, Kolte D, Gupta T, Subramanian KS, Khanna N, Aronow WS, et al. Temporal Trends and Sex Differences in Revascularization and Outcomes of ST-Segment Elevation Myocardial Infarction in Younger Adults in the United States. J Am Coll Cardiol. 2015 Nov;66(18):1961–72.
186.
Khera S, Kolte D, Palaniswamy C, Mujib M, Aronow WS, Singh T, et al. ST-elevation myocardial infarction in the elderly–temporal trends in incidence, utilization of percutaneous coronary intervention and outcomes in the United States. Int J Cardiol. 2013 Oct;168(4):3683–90.
187.
Khesroh AA, Al-Roumi F, Al-Zakwani I, Attur S, Rashed W, Zubaid M. Gender Differences among Patients with Acute Coronary Syndrome in the Middle East. Heart Views. 2017 Jul;18(3):77–82.
188.
Khraishah H, Alahmad B, Alfaddagh A, Jeong SY, Mathenge N, Kassab MB, et al. Sex disparities in the presentation, management and outcomes of patients with acute coronary syndrome: insights from the ACS QUIK trial. Open Heart [Internet]. 2021;8(1). Available from:

https://www.embase.com/search/results?subaction=viewrecord&id=L634093653&from=export
http://dx.doi.org/10.1136/openhrt-2020-001470
189.
Khraishah H, Karout L, Jeong SY, Alahmad B, AlAshqar A, Belanger MJ, et al. Clinical characteristics and cardiovascular outcomes among young patients with acute myocardial infarction in Kerala, India: A secondary analysis of ACS QUIK trial. Atherosclerosis Plus. 2022;50:25–31.
190.
Kilickap M, Erol MK, Kayikcioglu M, Kocayigit I, Gitmez M, Can V, et al. Short and Midterm Outcomes in Patients With Acute Myocardial Infarction: Results of the Nationwide TURKMI Registry. Angiology. 2021 Apr;72(4):339–47.
191.
Kim K, Lee TA, Touchette DR, DiDomenico RJ, Ardati AK, Walton SM. Contemporary Trends in Oral Antiplatelet Agent Use in Patients Treated with Percutaneous Coronary Intervention for Acute Coronary Syndrome. J Manag Care Spec Pharm. 2017 Jan;23(1):57–63.
192.
Kim SR, Bae S, Lee JY, Kim MS, Kim MN, Chung WJ, et al. Gender disparities in prevalence by diagnostic criteria, treatment and mortality of newly diagnosed acute myocardial infarction in Korean adults. Sci Rep. 2023 Mar;13(1):4120.
193.
Kim YH, Her AY, Rha SW, Choi CU, Choi BG, Kim JB, et al. Sex Differences in Delayed Hospitalization in Patients with Non-ST-Segment Elevation Myocardial Infarction Undergoing New-Generation Drug-Eluting Stent Implantation. Journal of Clinical Medicine [Internet]. 2023;12(5). Available from:
https://www.embase.com/search/results?subaction=viewrecord&id=L2022039342&from=export
http://dx.doi.org/10.3390/jcm12051982
194.
Kimenai DM, Lindahl B, Chapman AR, Baron T, Gard A, Wereski R, et al. Sex differences in investigations and outcomes among patients with type 2 myocardial infarction. Heart. 2021 Sep;107(18):1480–6.
195.
Kinnaird T, Kwok CS, Davies R, Calvert PA, Anderson R, Gallagher S, et al. Coronary perforation complicating percutaneous coronary intervention in patients presenting with an acute coronary syndrome: An analysis of 1013 perforation cases from the British Cardiovascular Intervention Society database. Int J Cardiol. 2020 Jan;299:37–42.
196.
Kitzmiller JP, Foraker RE, Rose KM. Lipid-lowering pharmacotherapy and socioeconomic status: Atherosclerosis Risk In Communities (ARIC) surveillance study. BMC Public Health. 2013 May;13:488.
197.
Klutstein MW, Westerhout CM, Armstrong PW, Giugliano RP, Lewis BS, Gibson CM, et al. Radial versus femoral access, bleeding and ischemic events in patients with non-ST-segment elevation acute coronary syndrome managed with an invasive strategy. Am Heart J. 2013 Apr;165(4):583-590.e1.
198.
Kodaira M, Sawano M, Tanaka M, Kuno T, Numasawa Y, Ueda I, et al. Female sex as an independent predictor of high bleeding risk among East Asian percutaneous coronary intervention patients: A sex difference analysis. J Cardiol. 2021 Nov;78(5):431–8.
199.
Kołodziej M, Kurzawski J, Janion-Sadowska A, Gierlotka M, Polonski L, Gasior M, et al. Mortality of women with ST-segment elevation myocardial infarction and cardiogenic shock - Results from the PL-ACS registry. Medical Studies/Studia Medyczne. 2016;32(3):157–63.
200.
Kontsevaya AV, Bates K, Goryachkin EA, Bobrova N, Syromiatnikova LI, Popova YV, et al. Hospital stage of myocardial infarction treatment in 13 regions of Russian Federation by results of the international research. Rational Pharmacotherapy in Cardiology. 2018;14(4):474–87.
201.

Koopman C, Vaartjes I, Heintjes EM, Spiering W, van Dis I, Herings RM, et al. Persisting gender differences and attenuating age differences in cardiovascular drug use for prevention and treatment of coronary heart disease, 1998-2010. Eur Heart J. 2013 Nov;34(41):3198–205.
202.
Kosmidou I, Redfors B, Selker HP, Thiele H, Patel MR, Udelson JE, et al. Infarct size, left ventricular function, and prognosis in women compared to men after primary percutaneous coronary intervention in ST-segment elevation myocardial infarction: results from an individual patient-level pooled analysis of 10 randomized trials. Eur Heart J. 2017 Jun;38(21):1656–63.
203.
Krishnamurthy A, Keeble C, Burton-Wood N, Somers K, Anderson M, Harl, et al. Clinical outcomes following primary percutaneous coronary intervention for ST-elevation myocardial infarction according to sex and race. Eur Heart J Acute Cardiovasc Care. 2019 Apr;8(3):264–72.
204.
Kuehnemund L, Koeppe J, Feld J, Wiederhold A, Illner J, Makowski L, et al. Gender differences in acute myocardial infarction-A nationwide German real-life analysis from 2014 to 2017. Clin Cardiol. 2021 Jul;44(7):890–8.
205.
Kuehnemund L, Lange SA, Feld J, Padberg JS, Fischer AJ, Makowski L, et al. Sex disparities in guideline-recommended therapies and outcomes after ST-elevation myocardial infarction in a contemporary nationwide cohort of patients over an eight-year period. Atherosclerosis. 2023 Jun;375:30–7.
206.
Kuhn L, Page K, Rahman MA, Worrall-Carter L. Gender difference in treatment and mortality of patients with ST-segment elevation myocardial infarction admitted to Victorian public hospitals: a retrospective database study. Aust Crit Care. 2015 Nov;28(4):196–202.
207.
Kuhn L, Worrall-Carter L, Ward J, Page K. Factors associated with delayed treatment onset for acute myocardial infarction in Victorian emergency departments: a regression tree analysis. Australas Emerg Nurs J. 2013 Nov;16(4):160–9.
208.
Kunadian V, Qiu W, Bawamia B, Veerasamy M, Jamieson S, Zaman A. Gender comparisons in cardiogenic shock during ST elevation myocardial infarction treated by primary percutaneous coronary intervention. Am J Cardiol. 2013 Sep;112(5):636–41.
209.
Kurlansky PA, Traad EA, Dorman MJ, Galbut DL, Zucker M, Ebra G. Bilateral internal mammary artery grafting reverses the negative influence of gender on outcomes of coronary artery bypass grafting surgery. European Journal of Cardio-thoracic Surgery. 2013;44(1):54–63.
210.
Kwok CS, Kontopantelis E, Kunadian V, Anderson S, Ratib K, Sperrin M, et al. Effect of access site, gender, and indication on clinical outcomes after percutaneous coronary intervention: Insights from the British Cardiovascular Intervention Society (BCIS). Am Heart J. 2015 Jul;170(1):164–72, 172.e1-5.
211.
Kytö V, Prami T, Khanfir H, Hasvold P, Reissell E, Airaksinen J. Usage of PCI and long-term cardiovascular risk in post-myocardial infarction patients: a nationwide registry cohort study from Finland. BMC Cardiovasc Disord. 2019 May;19(1):123.
212.
Kytö V, Sipilä J, Rautava P. Gender and in-hospital mortality of ST-segment elevation myocardial infarction (from a multihospital nationwide registry study of 31,689 patients). Am J Cardiol. 2015 Feb;115(3):303–6.
213.
Kytö V, Sipilä J, Rautava P, Gunn J. Sex Differences in Outcomes Following Acute Coronary Syndrome Treated With Coronary Artery Bypass Surgery. Heart Lung Circ. 2021 Jan;30(1):100–7.
214.

Lam CS, McEntegart M, Claggett B, Liu J, Skali H, Lewis E, et al. Sex differences in clinical characteristics and outcomes after myocardial infarction: insights from the Valsartan in Acute Myocardial Infarction Trial (VALIANT). Eur J Heart Fail. 2015 Mar;17(3):301–12.
215.
Lambert LJ, Brown KA, Boothroyd LJ, Segal E, Maire S, Kouz S, et al. Transfer of patients with ST-elevation myocardial infarction for primary percutaneous coronary intervention: a province-wide evaluation of "door-in to door-out" delays at the first hospital. Circulation. 2014 Jun;129(25):2653–60.
216.
Lana MLL, Beaton AZ, Brant LCC, Bozzi I, de Magalhães O, Castro LRA, et al. Factors associated with compliance to AHA/ACC performance measures in a myocardial infarction system of care in Brazil. Int J Qual Health Care. 2017 Aug;29(4):499–506.
217.
Langabeer 2nd JR, Champagne-Langabeer T, Fowler R, Henry T. Gender-based outcome differences for emergency department presentation ofnon-STEMI acute coronary syndrome. Am J Emerg Med. 2019 Feb;37(2):179–82.
218.
Langabeer 2nd JR, Henry TD, Fowler R, Champagne-Langabeer T, Kim J, Jacobs AK. Sex-Based Differences in Discharge Disposition and Outcomes for ST-Segment Elevation Myocardial Infarction Patients Within a Regional Network. J Womens Health (Larchmt). 2018 Aug;27(8):1001–6.
219.
Lapostolle F, Bataille S, Loyeau A, Simon B, Laborne FX, Dupas F, et al. Decision to deploy coronary reperfusion is not affected by the volume of ST-segment elevation myocardial infarction patients managed by prehospital emergency medical teams. Eur J Emerg Med. 2019 Dec;26(6):423–7.
220.
Lapostolle F, Loyeau A, Beggaz Y, Boche T, Pires V, Le Bail G, et al. Effect of age, gender, and time of day on pain-to-call times in patients with acute ST-segment elevation myocardial infarction: the CLOC'AGE study. Emergencias. 2021 Jun;33(3):181–6.
221.
Laufer-Perl M, Shacham Y, Letourneau-Shesaf S, Priesler O, Keren G, Roth A, et al. Gender-related mortality and in-hospital complications following ST-segment elevation myocardial infarction: data from a primary percutaneous coronary intervention cohort. Clin Cardiol. 2015 Mar;38(3):145–9.
222.
Laverty AA, Bottle A, Kim SH, Visani B, Majeed A, Millett C, et al. Gender differences in hospital admissions for major cardiovascular events and procedures in people with and without diabetes in England: a nationwide study 2004-2014. Cardiovasc Diabetol. 2017 Aug;16(1):100.
223.
Lawesson SS, Alfredsson J, Fredrikson M, Swahn E. A gender perspective on short- and long term mortality in ST-elevation myocardial infarction–a report from the SWEDEHEART register. Int J Cardiol. 2013 Sep;168(2):1041–7.
224.
Lawless M, Appelman Y, Beltrame JF, Navarese EP, Ratcovich H, Wilkinson C, et al. Sex differences in treatment and outcomes amongst myocardial infarction patients presenting with and without obstructive coronary arteries: a prospective multicentre study. Eur Heart J Open. 2023 Mar;3(2):oead033.
225.
Lee B, Lee SJ, Kim BK, Lee YJ, Hong SJ, Ahn CM, et al. Sex Differences in Outcomes of Ticagrelor Therapy With or Without Aspirin After Percutaneous Coronary Intervention in Patients With Acute Coronary Syndrome: A Post Hoc Secondary Analysis of the TICO Randomized Clinical Trial. Arterioscler Thromb Vasc Biol. 2023 Jun;43(6):e218–26.
226.
Lee CY, Hairi NN, Wan Ahmad WA, Ismail O, Liew HB, Zambahari R, et al. Are there gender differences in coronary artery disease? The Malaysian National Cardiovascular Disease Database - Percutaneous Coronary Intervention (NCVD-PCI) Registry. PLoS One. 2013;8(8):e72382.
227.
Lee CY, Liu KT, Lu HT, Mohd Ali R, Fong AYY, Wan Ahmad WA. Sex and gender differences in presentation, treatment and outcomes in acute coronary syndrome, a 10 year study from a multi-ethnic Asian population: The

Malaysian National Cardiovascular Disease Database-Acute Coronary Syndrome (NCVD-ACS) registry. PLoS One. 2021;16(2):e0246474.
228.
Lee JH, Bae MH, Yang DH, Park HS, Cho Y, Jeong MH, et al. Contemporary Trends of Optimal Evidence-Based Medical Therapy at Discharge for Patients Surviving Acute Myocardial Infarction From the Korea Acute Myocardial Infarction Registry. Clin Cardiol. 2015 Jun;38(6):350–6.
229.
Lee KK, Ferry AV, Anand A, Strachan FE, Chapman AR, Kimenai DM, et al. Sex-Specific Thresholds of High-Sensitivity Troponin in Patients With Suspected Acute Coronary Syndrome. J Am Coll Cardiol. 2019 Oct;74(16):2032–43.
230.
Lee M, Kim DW, Park MW, Lee K, Chang K, Chung WS, et al. Gender differences in clinical outcomes of acute myocardial infarction undergoing percutaneous coronary intervention: Insights from the KAMIR-NIH Registry. Journal of Geriatric Cardiology. 2020;17(11):680–93.
231.
Lee SH, Kim HK, Jeong MH, Lee JM, Gwon HC, Chae SC, et al. Pre-hospital delay and emergency medical services in acute myocardial infarction. Korean J Intern Med. 2020 Jan;35(1):119–32.
232.
Lemor A, Dabbagh MF, Cohen D, Villablanca P, Tehrani B, Alaswad K, et al. Rates and impact of vascular complications in mechanical circulatory support. Catheter Cardiovasc Interv. 2022 Apr;99(5):1702–11.
233.
Lempereur M, Magne J, Cornelis K, Hanet C, Taeymans Y, Vrolix M, et al. Impact of gender difference in hospital outcomes following percutaneous coronary intervention. Results of the Belgian Working Group on Interventional Cardiology (BWGIC) registry. EuroIntervention. 2016 Jun;12(2):e216-23.
234.
Leng W, Yang J, Fan X, Sun Y, Xu H, Gao X, et al. Contemporary invasive management and in-hospital outcomes of patients with non-ST-segment elevation myocardial infarction in China: Findings from China Acute Myocardial Infarction (CAMI) Registry. Am Heart J. 2019 Sep;215:1–11.
235.
Leurent G, Garlantézec R, Auffret V, Hacot JP, Coudert I, Filippi E, et al. Gender differences in presentation, management and inhospital outcome in patients with ST-segment elevation myocardial infarction: data from 5000 patients included in the ORBI prospective French regional registry. Arch Cardiovasc Dis. 2014 May;107(5):291–8.
236.
Levy M, Chen Y, Clarke R, Guo Y, Lv J, Yu C, et al. Gender differences in use of invasive diagnostic and therapeutic procedures for acute ischaemic heart disease in Chinese adults. Heart. 2022 Feb;108(4):292–9.
237.
Li L, Zhang X, Wang Y, Yu X, Jia H, Hou J, et al. A Novel Risk Score to Predict In-Hospital Mortality in Patients With Acute Myocardial Infarction: Results From a Prospective Observational Cohort. Frontiers in Cardiovascular Medicine [Internet]. 2022;9. Available from:
https://www.embase.com/search/results?subaction=viewrecord&id=L2019171208&from=export
http://dx.doi.org/10.3389/fcvm.2022.840485
238.
Li Y. Diagnostic Model of In-Hospital Mortality in Patients with Acute ST-Segment Elevation Myocardial Infarction Used Artificial Intelligence Methods. Cardiology Research and Practice [Internet]. 2022;2022. Available from:
https://www.embase.com/search/results?subaction=viewrecord&id=L2018699232&from=export
http://dx.doi.org/10.1155/2022/8758617
239.
Li Y, Babazono A, Jamal A, Liu N, Yamao R. Population-based multilevel models to estimate the management strategies for acute myocardial infarction in older adults with dementia. Clinical Epidemiology. 2021;13:883–92.
240.

Liakopoulos OJ, Slottosch I, Wendt D, Welp H, Schiller W, Martens S, et al. Surgical revascularization for acute coronary syndromes: a report from the North Rhine-Westphalia surgical myocardial infarction registry. Eur J Cardiothorac Surg. 2020 Dec;58(6):1137–44.
241.
Lichtman JH, Leifheit EC, Safdar B, Bao H, Krumholz HM, Lorenze NP, et al. Sex Differences in the Presentation and Perception of Symptoms Among Young Patients With Myocardial Infarction: Evidence from the VIRGO Study (Variation in Recovery: Role of Gender on Outcomes of Young AMI Patients). Circulation. 2018 Feb;137(8):781–90.
242.
Lin CF, Shen LJ, Hsiao FY, Gau CS, Wu FL. Sex differences in the treatment and outcome of patients with acute coronary syndrome after percutaneous coronary intervention: a population-based study. J Womens Health (Larchmt). 2014 Mar;23(3):238–45.
243.
Lin DS, Lin YS, Lee JK, Kao HL. Sex differences following percutaneous coronary intervention or coronary artery bypass surgery for acute myocardial infarction. Biol Sex Differ. 2022 Apr;13(1):18.
244.
Lin WC, Ho CH, Tung LC, Ho CC, Chou W, Wang CH. Differences Between Women and Men in Phase I Cardiac Rehabilitation After Acute Myocardial Infarction: A Nationwide Population-Based Analysis. Medicine (Baltimore). 2016 Jan;95(3):e2494.
245.
Lin Y, Pan W, Ning S, Song X, Jin Z, Lv S. Prevalence and management of hypertension in patients with acute coronary syndrome vary with gender: Observations from the Chinese registry of acute coronary events (CRACE). Mol Med Rep. 2013 Jul;8(1):173–7.
246.
Liu C, Yan HB, Zhao HJ, Song L, Zheng B, Chi YP, et al. Associated factors with repeat coronary angioplasty during the drug eluting stent era: a high volume center investigation. Chin Med J (Engl). 2013 Feb;126(3):446–9.
247.
Liu J, Elbadawi A, Elgendy IY, Megaly M, Ogunbayo GO, Krittanawong C, et al. Age-Stratified Sex Disparities in Care and Outcomes in Patients With ST-Elevation Myocardial Infarction. Am J Med. 2020 Nov;133(11):1293-1301.e1.
248.
Lopez-de-Andres A, Jimenez-Garcia R, Hernández-Barrera V, de Miguel-Yanes JM, Albaladejo-Vicente R, Villanueva-Orbaiz R, et al. Are there sex differences in the effect of type 2 diabetes in the incidence and outcomes of myocardial infarction? A matched-pair analysis using hospital discharge data. Cardiovasc Diabetol. 2021 Apr;20(1):81.
249.
Lu HT, Nordin R, Wan Ahmad WA, Lee CY, Zambahari R, Ismail O, et al. Sex differences in acute coronary syndrome in a multiethnic asian population: results of the malaysian national cardiovascular disease database-acute coronary syndrome (NCVD-ACS) registry. Glob Heart. 2014 Dec;9(4):381–90.
250.
Lu Y, Zhou S, Dreyer RP, Caulfield M, Spatz ES, Geda M, et al. Sex differences in lipid profiles and treatment utilization among young adults with acute myocardial infarction: Results from the VIRGO study. Am Heart J. 2017 Jan;183:74–84.
251.
Lv J, Ni L, Liu K, Gao X, Yang J, Zhang X, et al. Clinical Characteristics, Prognosis, and Gender Disparities in Young Patients With Acute Myocardial Infarction. Front Cardiovasc Med. 2021;8:720378.
252.
Macín SM, Del Sueldo M, Perna ER, Tajer CD, Cerezo GH, Struminger M, et al. Clinical characteristics and in-hospital evolution of women with acute myocardial infarction in the SAC-FAC National Registry of Infarction. Revista de la Federacion Argentina de Cardiologia. 2018;47(3):125–9.
253.
Mahajan K, Negi PC, Merwaha R, Mahajan N, Chauhan V, Asotra S. Gender differences in the management of acute coronary syndrome patients: One year results from HPIAR (HP-India ACS Registry). Int J Cardiol. 2017 Dec;248:1–6.

254.
Mahmoud AN, Elgendy IY. Gender Impact on 30-Day Readmissions After Hospitalization With Acute Myocardial Infarction Complicated by Cardiogenic Shock (from the 2013 to 2014 National Readmissions Database). Am J Cardiol. 2018 Mar;121(5):523–8.
255.
Mahowald MK, Alqahtani F, Alkhouli M. Comparison of Outcomes of Coronary Revascularization for Acute Myocardial Infarction in Men Versus Women. Am J Cardiol. 2020 Oct;132:1–7.
256.
Malanchini G, Stefanini GG, Malanchini M, Lombardi F. Higher in-hospital mortality during weekend admission for acute coronary syndrome: a large-scale cross-sectional Italian study. J Cardiovasc Med (Hagerstown). 2019 Feb;20(2):74–80.
257.
Malik AH, Siddiqui N, Aronow WS. Unstable angina: Trends and characteristics associated with length of hospitalization in the face of diminishing frequency—an evidence of a paradigm shift. Annals of Translational Medicine [Internet]. 2018;6(23). Available from:
https://www.embase.com/search/results?subaction=viewrecord&id=L625419721&from=export
http://dx.doi.org/10.21037/atm.2018.11.10
258.
Manzo-Silberman S, Couturaud F, Charpentier S, Auffret V, El Khoury C, Le Breton H, et al. Influence of gender on delays and early mortality in ST-segment elevation myocardial infarction: Insight from the first French Metaregistry, 2005-2012 patient-level pooled analysis. Int J Cardiol. 2018 Jul;262:1–8.
259.
Maor E, Abend Y, Ganem D, Kusniec F, Grosman-Rimon L, Elbaz-Greener G, et al. Sex Disparities in First Medical Contact of Patients with Suspected Acute Coronary Syndrome Using Telemedicine Technology. Telemed J E Health. 2020 Apr;26(4):411–8.
260.
Margolis G, Letourneau-Shesaf S, Khoury S, Pereg D, Kofman N, Keren G, et al. Trends and predictors of prehospital delay in patients undergoing primary coronary intervention. Coron Artery Dis. 2018 Aug;29(5):373–7.
261.
Mariani JA, Antonietti L, Tajermtsac CD, de Abreu M, Charask A, Silberstein M, et al. Gender differences in the treatment of acute coronary syndromes: Results of the Epi-Cardio registry. Revista Argentina de Cardiologia. 2013;81(4):307–15.
262.
Matetic A, Shamkhani W, Rashid M, Volgman AS, Van Spall HGC, Coutinho T, et al. Trends of Sex Differences in Clinical Outcomes After Myocardial Infarction in the United States. CJC Open. 2021;3(12):S19–27.
263.
Mathew A, Hong Y, Yogasundaram H, Nagendran J, Punnoose E, Ashraf SM, et al. Sex and Medium-term Outcomes of ST-Segment Elevation Myocardial Infarction in Kerala, India: A Propensity Score–Matched Analysis. CJC Open. 2021;3(12):S71–80.
264.
McDonald D, Wright A, Schmidt C. Complications in STEMI (ST segment elevation myocardial infarction) due to BMI (Body Mass Index). Am J Emerg Med. 2020 Nov;38(11):2474–5.
265.
McDonald N, Little N, Grierson R, Weldon E. Sex and Gender Equity in Prehospital Electrocardiogram Acquisition. Prehosp Disaster Med. 2022 Mar;37(2):1–7.
266.
Meyer MR, Bernheim AM, Kurz DJ, O'Sullivan CJ, Tüller D, Zbinden R, et al. Gender differences in patient and system delay for primary percutaneous coronary intervention: current trends in a Swiss ST-segment elevation myocardial infarction population. Eur Heart J Acute Cardiovasc Care. 2019 Apr;8(3):283–90.
267.
Mirić D, Novak K, Kovacević LM, Zanchi J. In-hospital mortality of patients with acute myocardial infarction before and after introduction of PCI in Split University Hospital Center, Croatia. Coll Antropol. 2013 Mar;37(1):207–12.

268.
Mnatzaganian G, Braitberg G, Hiller JE, Kuhn L, Chapman R. Sex differences in in-hospital mortality following a first acute myocardial infarction: symptomatology, delayed presentation, and hospital setting. BMC Cardiovasc Disord. 2016 May;16(1):109.
269.
Mnatzaganian G, Hiller JE, Fletcher J, Putl, M., Knott C, et al. Socioeconomic gradients in admission to coronary or intensive care units among Australians presenting with non-traumatic chest pain in emergency departments. BMC Emerg Med. 2018 Sep;18(1):32.
270.
Mohamed MO, Rashid M, Farooq S, Siddiqui N, Parwani P, Shiers D, et al. Acute Myocardial Infarction in Severe Mental Illness: Prevalence, Clinical Outcomes, and Process of Care in U.S. Hospitalizations. Can J Cardiol. 2019 Jul;35(7):821–30.
271.
Montoy JCC, Shen YC, Hsia RY. Trends in Inequities in the Treatment of and Outcomes for Women and Minorities with Myocardial Infarction. Ann Emerg Med. 2022 Aug;80(2):108–17.
272.
Morton JI, Ilomäki J, Wood SJ, Bell JS, Huynh Q, Magliano DJ, et al. Treatment gaps, 1-year readmission and mortality following myocardial infarction by diabetes status, sex and socioeconomic disadvantage. J Epidemiol Community Health. 2022 Jul;76(7):637–45.
273.
Movahed MR, Khan MF, Hashemzadeh M, Hashemzadeh M. Gradual decline in the age-adjusted in-hospital mortality rate from STEMI-related cardiogenic shock irrespective of cause, race or gender with persistent higher mortality rates in women despite multivariate adjustment. J Invasive Cardiol. 2014 Jan;26(1):7–12.
274.
Murphy AC, Yudi MB, Farouque O, Dinh D, Duffy SJ, Brennan A, et al. Impact of Gender and Door-to-Balloon Times on Long-Term Mortality in Patients Presenting With ST-Elevation Myocardial Infarction. Am J Cardiol. 2019 Sep;124(6):833–41.
275.
Nagumo S, Mori H, Maeda A, Akashi YJ, Ako J, Ikari Y, et al. Sex-Related Differences in In-Hospital Mortality in Japanese ST-Elevation Acute Myocardial Infarction Patients Presenting to Hospital in the 24 Hours After Symptom Onset - Results From K-ACTIVE. Circ Rep. 2019 Jul;1(8):313–9.
276.
Nan Tie E, Fern, o H, Nehme Z, Dinh D, Andrew E, et al. Sex differences in prehospital analgesia in patients presenting with acute coronary syndromes and their association with clinical outcomes. Catheter Cardiovasc Interv. 2022 Mar;99(4):989–95.
277.
Nanna MG, Hajduk AM, Krumholz HM, Murphy TE, Dreyer RP, Alex, et al. Sex-Based Differences in Presentation, Treatment, and Complications Among Older Adults Hospitalized for Acute Myocardial Infarction: The SILVER-AMI Study. Circ Cardiovasc Qual Outcomes. 2019 Oct;12(10):e005691.
278.
Nazzal C, Alonso FT. Las mujeres jóvenes en Chile tienen elevado riesgo de muerte intrahospitalaria por infarto de miocardio. Revista Espanola de Cardiologia. 2013;66(2):104–9.
279.
Neumann JT, Goßling A, Sörensen NA, Blankenberg S, Magnussen C, Westermann D. Sex-specific outcomes in patients with acute coronary syndrome. Journal of Clinical Medicine. 2020;9(7):1–13.
280.
Ngiam JN, Thong EH, Loh PH, Chan KH, Chan MY, Lee CH, et al. An Asian Perspective on Gender Differences in In-Hospital and Long-Term Outcome of Cardiac Mortality and Ischemic Stroke after Primary Percutaneous Coronary Intervention for ST-Segment Elevation Myocardial Infarction. J Stroke Cerebrovasc Dis. 2022 Jan;31(1):106215.
281.

Nielsen CG, Laut KG, Jensen LO, Ravkilde J, Terkelsen CJ, Kristensen SD. Patient delay in patients with ST-elevation myocardial infarction: Time patterns and predictors for a prolonged delay. Eur Heart J Acute Cardiovasc Care. 2017 Oct;6(7):583–91.
282.
Novak K, Vrdoljak D, Jelaska I, Borovac JA. Sex-specific differences in risk factors for in-hospital mortality and complications in patients with acute coronary syndromes : An observational cohort study. Wien Klin Wochenschr. 2017 Apr;129(7):233–42.
283.
Numasawa Y, Inohara T, Ishii H, Kuno T, Kodaira M, Kohsaka S, et al. Comparison of Outcomes of Women Versus Men With Non-ST-elevation Acute Coronary Syndromes Undergoing Percutaneous Coronary Intervention (from the Japanese Nationwide Registry). Am J Cardiol. 2017 Mar;119(6):826–31.
284.
Ogbu I, Ayutyanont N, Wilson S, Akhondi H. The Impact of Gender and Race When Using the GRACE ACS Score to Predict Mortality. HCA Healthc J Med. 2023;4(3):235–42.
285.
Ogunbayo GO, Bidwell K, Misumida N, Ha LD, Abdel-Latif A, Elayi CS, et al. Sex differences in the contemporary management of HIV patients admitted for acute myocardial infarction. Clin Cardiol. 2018 Apr;41(4):488–93.
286.
Olier I, Carr M, Curzen N, Ludman P, Baumbach A, Kinnaird T, et al. Changes in Periprocedural Bleeding Complications Following Percutaneous Coronary Intervention in The United Kingdom Between 2006 and 2013 (from the British Cardiovascular Interventional Society). Am J Cardiol. 2018 Sep;122(6):952–60.
287.
Osman M, Ghaffar YA, Osman K, Kheiri B, Mohamed MMG, Kawsara A, et al. Gender-based outcomes of coronary bifurcation stenting: A report from the National Readmission Database. Catheter Cardiovasc Interv. 2022 Feb;99(2):433–9.
288.
Osman M, Syed M, Kheiri B, Bianco C, Kalra A, Cigarroa JE, et al. Age stratified sex-related differences in incidence, management, and outcomes of cardiogenic shock. Catheter Cardiovasc Interv. 2022 Jun;99(7):1984–95.
289.
Otten AM, Maas AH, Ottervanger JP, Kloosterman A, van 't Hof AW, Dambrink JH, et al. Is the difference in outcome between men and women treated by primary percutaneous coronary intervention age dependent? Gender difference in STEMI stratified on age. Eur Heart J Acute Cardiovasc Care. 2013 Dec;2(4):334–41.
290.
Ouellet GM, Geda M, Murphy TE, Tsang S, Tinetti ME, Chaudhry SI. Prehospital Delay in Older Adults with Acute Myocardial Infarction: The ComprehenSIVe Evaluation of Risk Factors in Older Patients with Acute Myocardial Infarction Study. J Am Geriatr Soc. 2017 Nov;65(11):2391–6.
291.
P, ey A, McGuire DK, de Lemos JA, Das SR, Berry JD, et al. Revascularization Trends in Patients With Diabetes Mellitus and Multivessel Coronary Artery Disease Presenting With Non-ST Elevation Myocardial Infarction: Insights From the National Cardiovascular Data Registry Acute Coronary Treatment and Intervention Outcomes Network Registry-Get with the Guidelines (NCDR ACTION Registry-GWTG). Circ Cardiovasc Qual Outcomes. 2016 May;9(3):197–205.
292.
Pagidipati NJ, Huffman MD, Jeemon P, Gupta R, Negi P, Jaison TM, et al. Association between gender, process of care measures, and outcomes in ACS in India: results from the detection and management of coronary heart disease (DEMAT) registry. PLoS One. 2013;8(4):e62061.
293.
Pandie S, Mehta SR, Cantor WJ, Cheema AN, Gao P, Madan M, et al. Radial Versus Femoral Access for Coronary Angiography/Intervention in Women With Acute Coronary Syndromes: Insights From the RIVAL Trial (Radial Vs femorAL access for coronary intervention). JACC Cardiovasc Interv. 2015 Apr;8(4):505–12.
294.

Park HW, Han S, Park GM, Ann SH, Suh J, Kim YG, et al. Sex-related impacts on clinical outcomes after percutaneous coronary intervention. Sci Rep. 2020 Sep;10(1):15262.
295.
Park YJ, Lee JH, Kim HJ, Park BE, Kim HN, Jang SY, et al. Variation in treatment strategy for non-ST segment elevation myocardial infarction: A multilevel methodological approach. Int J Cardiol. 2021 Apr;328:35–9.
296.
Patel A, Vishwanathan S, Nair T, Bahuleyan CG, Jayaprakash VL, Baldridge A, et al. Sex Differences in the Presentation, Diagnosis, and Management of Acute Coronary Syndromes: Findings From the Kerala-India ACS Registry. Glob Heart. 2015 Dec;10(4):273–80.
297.
Patel KN, Majmundar M, Vasudeva R, Doshi R, Kaur A, Mehta H, et al. Impact of Gender, Race, and Insurance Status on Inhospital Management and Outcomes in Patients With COVID-19 and ST-Elevation Myocardial Infarction (a Nationwide Analysis). Am J Cardiol. 2023 Jul;198:14–25.
298.
Patel SJ, Ajebo G, Kota V, Guddati AK. Outcomes of hospitalized patients with myocardial infarction and immune thrombocytopenic purpura: A cross sectional study over 15 years. American Journal of Blood Research. 2020;10(5):210–6.
299.
Peerwani G, Khan SM, Khan MD, Bashir F, Sheikh S, Ramsey DJ, et al. Gender Differences in Clinical Outcomes After Percutaneous Coronary Intervention-Analysis of 15,106 Patients from the Cardiac Registry of Pakistan Database. Am J Cardiol. 2023 Feb;188:61–7.
300.
Pelletier R, Humphries KH, Shimony A, Bacon SL, Lavoie KL, Rabi D, et al. Sex-related differences in access to care among patients with premature acute coronary syndrome. Cmaj. 2014 Apr;186(7):497–504.
301.
Pereira M, Araújo C, Dias P, Lunet N, Subirana I, Marrugat J, et al. Age and sex inequalities in the prescription of evidence-based pharmacological therapy following an acute coronary syndrome in Portugal: the EURHOBOP study. Eur J Prev Cardiol. 2014 Nov;21(11):1401–8.
302.
Perl L, Bental T, Assali A, Vaknin-Assa H, Lev E, Kornowski R, et al. Impact of female sex on long-term acute coronary syndrome outcomes. Coronary Artery Disease [Internet]. 2014; Available from: https://www.embase.com/search/results?subaction=viewrecord&id=L53301127&from=export http://dx.doi.org/10.1097/MCA.0000000000000164
303.
Perl L, Peiffer V, Fuhrer AE, D'Ascenzo F, Pietzsch JB. Sex differences in discharge destination following acute myocardial infarction. Coron Artery Dis. 2018 Sep;29(6):502–10.
304.
Pia, cedil, tek Ł, Wilczek K, Kurzawski J, Gierlotka M, et al. Gender-related disparities in the treatment and outcomes in patients with non-st-segment elevation myocardial infarction: Results from the Polish Registry of Acute Coronary Syndromes (PL-ACS) in the years 2012-2014. Archives of Medical Science. 2020;16(4):781–8.
305.
Piackova E, Jäger B, Farhan S, Christ G, Schreiber W, Weidinger F, et al. Gender differences in short- and long-term mortality in the Vienna STEMI registry. Int J Cardiol. 2017 Oct;244:303–8.
306.
Piątek Ł, Wilczek K, Janion-Sadowska A, Gierlotka M, Gąsior M, Sadowski M. Outcomes of a routine invasive strategy in elderly patients with non-ST-segment elevation myocardial infarction from 2005 to 2014: results from the PL-ACS registry. Coron Artery Dis. 2019 Aug;30(5):326–31.
307.
Pilgrim T, Heg D, Tal K, Erne P, Radovanovic D, Windecker S, et al. Age- and Gender-related Disparities in Primary Percutaneous Coronary Interventions for Acute ST-segment elevation Myocardial Infarction. PLoS One. 2015;10(9):e0137047.
308.

Plaza-Martín M, Sanmartin-Fernandez M, Álvarez-Álvarez B, Andrea R, Seoane-García T, González-D'Gregorio J, et al. Contemporary differences between men and women with acute coronary syndromes: CIAM multicenter registry. J Cardiovasc Med (Hagerstown). 2019 Aug;20(8):525–30.
309.
Poorhosseini H, Saadat M, Salarifar M, Mortazavi SH, Geraiely B. Pre-Hospital Delay and Its Contributing Factors in Patients with ST-Elevation Myocardial Infarction; a Cross sectional Study. Arch Acad Emerg Med. 2019;7(1):e29.
310.
Potts J, Sirker A, Martinez SC, Gulati M, Alasnag M, Rashid M, et al. Persistent sex disparities in clinical outcomes with percutaneous coronary intervention: Insights from 6.6 million PCI procedures in the United States. PLoS One. 2018;13(9):e0203325.
311.
Preciado SM, Sharp AL, Sun BC, Baecker A, Wu YL, Lee MS, et al. Evaluating Sex Disparities in the Emergency Department Management of Patients With Suspected Acute Coronary Syndrome. Ann Emerg Med. 2021 Apr;77(4):416–24.
312.
Qamar A, Bhatia K, Arora S, Hendrickson M, Gupta P, Fatima A, et al. Clinical Profiles, Outcomes, and Sex Differences of Patients With STEMI: Findings From the NORIN-STEMI Registry. JACC: Asia. 2023;3(3):431–42.
313.
Quinn T, Johnsen S, Gale CP, Snooks H, McLean S, Woollard M, et al. Effects of prehospital 12-lead ECG on processes of care and mortality in acute coronary syndrome: a linked cohort study from the Myocardial Ischaemia National Audit Project. Heart. 2014 Jun;100(12):944–50.
314.
Radomska E, Sadowski M, Kurzawski J, Gierlotka M, Polonski L. ST-segment elevation myocardial infarction in women with type 2 diabetes. Diabetes Care. 2013 Nov;36(11):3469–75.
315.
Radovanovic D, Seifert B, Roffi M, Urban P, Rickli H, Pedrazzini G, et al. Gender differences in the decrease of in-hospital mortality in patients with acute myocardial infarction during the last 20 years in Switzerland. Open Heart. 2017;4(2):e000689.
316.
Rajakariar K, Andrianopoulos N, Gayed D, Liang D, Backhouse B, Ajani AE, et al. Outcomes of thrombus aspiration during primary percutaneous coronary intervention for ST-elevation myocardial infarction. Intern Med J. 2022 Jun;
317.
Ram E, Sternik L, Moshkovitz Y, Iakobishvili Z, Zuroff E, Peled Y, et al. Coronary Artery Bypass Grafting Following Acute Coronary Syndrome: Impact of Gender. Semin Thorac Cardiovasc Surg. 2022;34(3):920–9.
318.
Rashid M, Curzen N, Kinnaird T, Lawson CA, Myint PK, Kontopantelis E, et al. Baseline risk, timing of invasive strategy and guideline compliance in NSTEMI: Nationwide analysis from MINAP. Int J Cardiol. 2020 Feb;301:7–13.
319.
Rashid M, Fischman DL, Martinez SC, Capers Q th, Savage M, Zaman A, et al. Temporal trends and predictors of time to coronary angiography following non-ST-elevation acute coronary syndrome in the USA. Coron Artery Dis. 2019 May;30(3):159–70.
320.
Rawshani A, Larsson A, Gelang C, Lindqvist J, Gellerstedt M, Bång A, et al. Characteristics and outcome among patients who dial for the EMS due to chest pain. International Journal of Cardiology [Internet]. 2014; Available from:
https://www.embase.com/search/results?subaction=viewrecord&id=L53308808&from=export http://dx.doi.org/10.1016/j.ijcard.2014.08.004
321.
Rayner-Hartley E, Wong GC, Fayowski C, Cairns JA, Singer J, Lee T, et al. Impact of regionalizing ST-elevation myocardial infarction care on sex differences in reperfusion times and clinical outcomes. Clin Cardiol. 2021 Aug;44(8):1113–9.
322.

Reda A, Ashraf M, Soliman M, Ragy H, El Kersh A, Abdou W, et al. The pattern of risk-factor profile in Egyptian patients with acute coronary syndrome: phase II of the Egyptian cross-sectional CardioRisk project. Cardiovasc J Afr. 2019 Mar;30(2):87–94.
323.
Reda A, Bendary A, Elbahry A, Farag E, Mostafa T, Khamis H, et al. Prevalence of atherosclerosis risk factors in Egyptian patients with acute coronary syndrome: Final data of the nationwide cross-sectional 'CardioRisk' project. Journal of Public Health in Africa. 2020;11(2):114–21.
324.
Redfors B, Angerås O, Råmunddal T, Petursson P, Haraldsson I, Dworeck C, et al. Trends in Gender Differences in Cardiac Care and Outcome After Acute Myocardial Infarction in Western Sweden: A Report From the Swedish Web System for Enhancement of Evidence-Based Care in Heart Disease Evaluated According to Recommended Therapies (SWEDEHEART). J Am Heart Assoc. 2015 Jul;4(7).
325.
Ricci B, Manfrini O, Cenko E, Vasiljevic Z, Dorobantu M, Kedev S, et al. Primary percutaneous coronary intervention in octogenarians. Int J Cardiol. 2016 Nov;222:1129–35.
326.
Riehle L, Gothe RM, Ebbinghaus J, Maier B, Bruch L, Röhnisch JU, et al. Implementation of the ESC STEMI guidelines in female and elderly patients over a 20-year period in a large German registry. Clin Res Cardiol. 2023 Sep;112(9):1240–51.
327.
Riehle L, Maier B, Behrens S, Bruch L, Schoeller R, Schühlen H, et al. Changes in treatment for NSTEMI in women and the elderly over the past 16 years in a large real-world population. Int J Cardiol. 2020 Oct;316:7–12.
328.
Roe YL, Zeitz CJ, Mittinty MN, McDermott RA, Chew DP. Impact of age, gender and indigenous status on access to diagnostic coronary angiography for patients presenting with non-ST segment elevation acute coronary syndromes in Australia. Intern Med J. 2013 Mar;43(3):317–22.
329.
Roffi M, Radovanovic D, Erne P, Urban P, Windecker S, Eberli FR. Gender-related mortality trends among diabetic patients with ST-segment elevation myocardial infarction: insights from a nationwide registry 1997-2010. Eur Heart J Acute Cardiovasc Care. 2013 Dec;2(4):342–9.
330.
Roque D, Ferreira J, Monteiro S, Costa M, Gil V. Understanding a woman's heart: Lessons from 14 177 women with acute coronary syndrome. Rev Port Cardiol (Engl Ed). 2020 Feb;39(2):57–72.
331.
Rosell-Ortiz F, Mellado-Vergel FJ, Fernández-Valle P, González-Lobato I, Martínez-Lara M, Ruiz-Montero MM, et al. Initial complications and factors related to prehospital mortality in acute myocardial infarction with ST segment elevation. Emerg Med J. 2015 Jul;32(7):559–63.
332.
Rossello X, Mas-Lladó C, Pocock S, Vicent L, van de Werf F, Chin CT, et al. Sex differences in mortality after an acute coronary syndrome increase with lower country wealth and higher income inequality. Rev Esp Cardiol (Engl Ed). 2022 May;75(5):392–400.
333.
Roswell RO, Kunkes J, Chen AY, Chiswell K, Iqbal S, Roe MT, et al. Impact of Sex and Contact-to-Device Time on Clinical Outcomes in Acute ST-Segment Elevation Myocardial Infarction-Findings From the National Cardiovascular Data Registry. J Am Heart Assoc. 2017 Jan;6(1).
334.
Ruane L, J HG, Parsonage W, Hawkins T, Hammett C, Lam CS, et al. Differences in Presentation, Management and Outcomes in Women and Men Presenting to an Emergency Department With Possible Cardiac Chest Pain. Heart Lung Circ. 2017 Dec;26(12):1282–90.
335.

Ruiz-Pizarro V, Ferrera C, Gómez-Polo JC, Palacios-Rubio J, Rico-García Amado C, Fernández-Ortiz A, et al. Sex differences in treatment and prognosis of acute coronary syndrome with interventional management. Cardiovasc Revasc Med. 2019 Mar;20(3):183–6.
336.
Saadatagah S, Ghodsi S, Omidi N, Poorhosseini H, Salarifar M, Sadeghian S, et al. Twelve-Year History of STEMI Management in Tehran Heart Center: Concomitant Reduction of In-Hospital Mortality and Hospitalization Length. Arch Iran Med. 2020 Aug;23(8):514–21.
337.
Sabbag A, Matetzky S, Porter A, Iakobishvili Z, Moriel M, Zwas D, et al. Sex Differences in the Management and 5-Year Outcome of Young Patients (<55 Years) with Acute Coronary Syndromes. Am J Med. 2017 Nov;130(11):1324.e15-1324.e22.
338.
Saito S, Isshiki T, Kimura T, Ogawa H, Yokoi H, Nishikawa M, et al. Impact of Arterial Access Route on Bleeding Complications in Japanese Patients Undergoing Percutaneous Coronary Intervention- Insight From the PRASFIT Trial. Circ J. 2015;79(9):1928–37.
339.
Sambola A, Elola FJ, Buera I, Fernández C, Bernal JL, Ariza A, et al. Sex bias in admission to tertiary-care centres for acute myocardial infarction and cardiogenic shock. Eur J Clin Invest. 2021 Jul;51(7):e13526.
340.
Sambola A, Elola FJ, Ferreiro JL, Murga N, Rodríguez-Padial L, Fernández C, et al. Impact of sex differences and network systems on the in-hospital mortality of patients with ST-segment elevation acute myocardial infarction. Rev Esp Cardiol (Engl Ed). 2021 Nov;74(11):927–34.
341.
Sanfilippo FM, Hillis GS, Rankin JM, Latchem D, Schultz CJ, Yong J, et al. Invasive Coronary Angiography after Chest Pain Presentations to Emergency Departments. Int J Environ Res Public Health. 2020 Dec;17(24).
342.
Santos H, Santos M, Almeida I, Paula SB, Chin J, Almeida S, et al. High-grade atrioventricular block in acute coronary syndrome: Portuguese experience. J Electrocardiol. 2021 Sep;68:130–4.
343.
Savage ML, Hay K, Murdoch DJ, Walters DL, Denman R, Ranasinghe I, et al. Sex differences in time to primary percutaneous coronary intervention and outcomes in patients presenting with ST-segment elevation myocardial infarction. Catheter Cardiovasc Interv. 2022 Oct;100(4):520–9.
344.
Schoos MM, Mehran R, Dangas GD, Yu J, Baber U, Clemmensen P, et al. Gender Differences in Associations Between Intraprocedural Thrombotic Events During Percutaneous Coronary Intervention and Adverse Outcomes. Am J Cardiol. 2016 Dec;118(11):1661–8.
345.
Settelmeier S, Rassaf T, Hochadel M, Voigtländer T, Münzel T, Senges J, et al. Gender Differences in Patients Admitted to a Certified German Chest Pain Unit: Results from the German Chest Pain Unit Registry. Cardiology. 2020;145(9):562–9.
346.
Shah P, Patel K, Vasudev R, Patel H, Thakkar S, Adalja D, et al. Gender differences in the revascularization rates and in-hospital outcomes in hospitalizations with ST segment elevation myocardial infarction. Ir J Med Sci. 2020 Aug;189(3):873–84.
347.
Shalaby G, Niazi AK, Khaled S. Cardiogenic Shock Among Patients with Acute ST-Segment Elevation Myocardial Infarction in a Middle Eastern Country: A Single-Center Experience. Journal of the Saudi Heart Association. 2022;34(4):233–40.
348.
Sharma YP, Santosh Vemuri K, Bootla D, Kanabar K, Pruthvi CR, Kaur N, et al. Epidemiological profile, management and outcomes of patients with acute coronary syndrome: Single centre experience from a tertiary care hospital in North India. Indian Heart J. 2021 Mar;73(2):174–9.

349.
Shavadia JS, Youngson E, Bainey KR, Bakal J, Welsh RC. Outcomes and Prognostic Impact of Prophylactic Oral Anticoagulation in Anterior ST-Segment Elevation Myocardial Infarction Patients With Left Ventricular Dysfunction. J Am Heart Assoc. 2017 Jul;6(7).
350.
Shavelle DM, Chen AY, Matthews RV, Roe MT, de Lemos JA, Jollis J, et al. Predictors of reperfusion delay in patients with ST elevation myocardial infarction self-transported to the hospital (from the American Heart Association's Mission: Lifeline Program). Am J Cardiol. 2014 Mar;113(5):798–802.
351.
Shehab A, Al-Dabbagh B, AlHabib KF, Alsheikh-Ali AA, Almahmeed W, Sulaiman K, et al. Gender disparities in the presentation, management and outcomes of acute coronary syndrome patients: data from the 2nd Gulf Registry of Acute Coronary Events (Gulf RACE-2). PLoS One. 2013;8(2):e55508.
352.
Shehab A, AlHabib KF, Bhagavathula AS, Hersi A, Alfaleh H, Alshamiri MQ, et al. Clinical Presentation, Quality of Care, Risk Factors and Outcomes in Women with Acute ST-Elevation Myocardial Infarction (STEMI): An Observational Report from Six Middle Eastern Countries. Curr Vasc Pharmacol. 2019;17(4):388–95.
353.
Shehab A, Bhagavathula AS, Alhabib KF, Ullah A, Suwaidi JA, Almahmeed W, et al. Age-Related Sex Differences in Clinical Presentation, Management, and Outcomes in ST-Segment-Elevation Myocardial Infarction: Pooled Analysis of 15 532 Patients From 7 Arabian Gulf Registries. J Am Heart Assoc. 2020 Feb;9(4):e013880.
354.
Shehab A, Yasin J, Hashim MJ, Al-Dabbagh B, Mahmeed WA, Bustani N, et al. Gender differences in acute coronary syndrome in Arab Emirati women–implications for clinical management. Angiology. 2013 Jan;64(1):9–14.
355.
Shetty LH, Patil RS, Kharge J, Vijay Kumar JR, Ghosh S, Manjunath CN. Gender Differences in South Indians with Premature Coronary Artery Disease (< 40 Years)-Insights from the PCAD Registry. Indian Journal of Cardiovascular Disease in Women - WINCARS. 2021;6(3):176–83.
356.
Shoji K, Yanishi K, Kawamata H, Hori Y, Fujioka A, Kohno Y, et al. New risk factors for early- and late-onset cardiac rupture in ST-elevation myocardial infarction patients after primary percutaneous coronary intervention. J Cardiol. 2022 Mar;79(3):400–7.
357.
Siddiqi HK, Defilippis EM, Biery DW, Singh A, Wu WY, Divakaran S, et al. Mortality and Heart Failure Hospitalization Among Young Adults With and Without Cardiogenic Shock After Acute Myocardial Infarction. J Card Fail. 2023 Jan;29(1):18–29.
358.
Sielski J, Kazirόd-Wolski K, Jurys K, Wałek P, Siudak Z. The Effect of Periprocedural Clinical Factors Related to the Course of STEMI in Men and Women Based on the National Registry of Invasive Cardiology Procedures (ORPKI) between 2014 and 2019. J Clin Med. 2021 Dec;10(23).
359.
Simon T, Puymirat E, Lucke V, Bouabdallaoui N, Lognoné T, Aissaoui N, et al. [Acute myocardial infarction in women. Initial characteristics, management and early outcome. The FAST-MI registry]. Ann Cardiol Angeiol (Paris). 2013 Aug;62(4):221–6.
360.
Singh V, Mendirichaga R, Savani GT, Rodriguez AP, Dabas N, Munagala A, et al. Coronary revascularization for acute myocardial infarction in the HIV population. J Interv Cardiol. 2017 Oct;30(5):405–14.
361.
Skelding KA, Boga G, Sartorius J, Wood GC, Berger PB, Mascarenhas VH, et al. Frequency of coronary angiography and revascularization among men and women with myocardial infarction and their relationship to mortality at one year: an analysis of the Geisinger myocardial infarction cohort. J Interv Cardiol. 2013 Feb;26(1):14–21.
362.

Smilowitz NR, Mahajan AM, Roe MT, Hellkamp AS, Chiswell K, Gulati M, et al. Mortality of Myocardial Infarction by Sex, Age, and Obstructive Coronary Artery Disease Status in the ACTION Registry-GWTG (Acute Coronary Treatment and Intervention Outcomes Network Registry-Get With the Guidelines). Circ Cardiovasc Qual Outcomes. 2017 Dec;10(12):e003443.
363.
Sobers N, Rose AMC, Samuels TA, Critchley J, Abed M, Hambleton I, et al. Are there gender differences in acute management and secondary prevention of acute coronary syndromes in Barbados? A cohort study. BMJ Open. 2019 Jan;9(1):e025977.
364.
Soeiro A, Matos re de, Silva PGM de B e, Roque EA de C, Bossa AS, Biselli B, et al. Prognostic Differences between Men and Women with Acute Coronary Syndrome. Data from a Brazilian Registry. Arq bras cardiol. 2018 Nov;111(5):648–53.
365.
Solinas E, Vignali L, Ortolani P, Guastaroba P, Marzocchi A, Manari A, et al. Association of bleeding, mortality and sex in acute coronary syndromes: the missing triangle. J Cardiovasc Med (Hagerstown). 2015 May;16(5):347–54.
366.
Sörensen NA, Neumann JT, Ojeda F, Schäfer S, Magnussen C, Keller T, et al. Relations of Sex to Diagnosis and Outcomes in Acute Coronary Syndrome. J Am Heart Assoc. 2018 Mar;7(6).
367.
Spatz ES, Wang Y, Beckman AL, Wu X, Lu Y, Du X, et al. Traditional Chinese Medicine for Acute Myocardial Infarction in Western Medicine Hospitals in China. Circ Cardiovasc Qual Outcomes. 2018 Mar;11(3):e004190.
368.
Stehli J, Dinh D, Dagan M, Duffy SJ, Brennan A, Smith K, et al. Sex Differences in Prehospital Delays in Patients With ST-Segment-Elevation Myocardial Infarction Undergoing Percutaneous Coronary Intervention. J Am Heart Assoc. 2021 Jul;10(13):e019938.
369.
Stehli J, Dinh D, Dagan M, Dick R, Oxley S, Brennan A, et al. Sex differences in treatment and outcomes of patients with in-hospital ST-elevation myocardial infarction. Clin Cardiol. 2022 Apr;45(4):427–34.
370.
Stehli J, Duffy SJ, Koh Y, Martin C, Brennan A, Dinh DT, et al. Sex Differences in Radial Access for Percutaneous Coronary Intervention in Acute Coronary Syndrome Are Independent of Body Size. Heart Lung Circ. 2021 Jan;30(1):108–14.
371.
Stehli J, Martin C, Brennan A, Dinh DT, Lefkovits J, Zaman S. Sex Differences Persist in Time to Presentation, Revascularization, and Mortality in Myocardial Infarction Treated With Percutaneous Coronary Intervention. J Am Heart Assoc. 2019 May;8(10):e012161.
372.
Steitieh DA, Lu DY, Kalil RK, Kim LK, Sharma G, Yeo I, et al. Sex-Based Differences in Revascularization and 30-Day Readmission After ST-Segment-Elevation Myocardial Infarction in the United States. Cardiovasc Revasc Med. 2021 Oct;31:41–7.
373.
Sulaiman S, Kawsara A, Mohamed MO, Van Spall HGC, Sutton N, Holmes DR, et al. Treatment Effect of Percutaneous Coronary Intervention in Men Versus Women With ST-Segment-Elevation Myocardial Infarction. J Am Heart Assoc. 2021 Sep;10(18):e021638.
374.
Sullivan AL, Beshansky JR, Ruthazer R, Murman DH, Mader TJ, Selker HP. Factors associated with longer time to treatment for patients with suspected acute coronary syndromes: a cohort study. Circ Cardiovasc Qual Outcomes. 2014 Jan;7(1):86–94.
375.
Sun Y, Feng L, Li X, Gao R, Wu Y. The sex difference in 6-month MACEs and its explaining variables in acute myocardial infarction survivors: Data from CPACS-3 study. Int J Cardiol. 2020 Jul;311:1–6.
376.

Tan J, Zhang YH, Si J, Xiao KL, Hua Q, Li J. Incidence, predictors and prognosis of acute kidney injury in acute ST-segment elevation myocardial infarction patients undergoing emergent coronary angiography/primary percutaneous coronary intervention. Journal of Geriatric Cardiology. 2023;20(2):139–49.
377.
Tang X, Liu L, Yang J, Gao Z, Zhao X, Qiao S, et al. Evidence-based oral antiplatelet therapy among hospitalized Chinese patients with acute myocardial infarction: results from the Chinese acute myocardial infarction registry. BMC Cardiovasc Disord. 2021 Jun;21(1):299.
378.
Tareen HN, Sher A, Goher F, Hussain I, Saif M, Khan MR. The Insight into the Sex Differences in the Patients Diagnosed with Acute Coronary Syndromes Undergoing Percutaneous Coronary Intervention. Pakistan Journal of Medical and Health Sciences. 2022;16(10):379–81.
379.
Tataris KL, Mercer MP, Govindarajan P. Prehospital aspirin administration for acute coronary syndrome (ACS) in the USA: an EMS quality assessment using the NEMSIS 2011 database. Emerg Med J. 2015 Nov;32(11):876–81.
380.
Tay JC, Lun LW, Liang Z, Chua TS, Lim SH, Wong AS, et al. Impact of Direct Cardiovascular Laboratory Activation by Emergency Physicians on False-Positive Activation Rates. Ann Acad Med Singap. 2016 Aug;45(8):351–6.
381.
Ten Haaf ME, Bax M, Ten Berg JM, Brouwer J, Van't Hof AW, van der Schaaf RJ, et al. Sex differences in characteristics and outcome in acute coronary syndrome patients in the Netherlands. Neth Heart J. 2019 May;27(5):263–71.
382.
Ten Haaf ME, van Geuns RJ, van der Linden M, Smits PC, de Vries AG, Doevendans PA, et al. Sex-related bleeding risk in acute coronary syndrome patients receiving dual antiplatelet therapy with aspirin and a P2Y12 inhibitor. Med Princ Pract. 2023 Mar;
383.
Ten Have P, Hilt AD, Paalvast H, Eindhoven DC, Schalij MJ, Beeres S. Non-ST-elevation myocardial infarction in the Netherlands: room for improvement! Neth Heart J. 2020 Oct;28(10):537–45.
384.
Thang ND, Karlson BW, Karlsson T, Herlitz J. Characteristics of and outcomes for elderly patients with acute myocardial infarction: differences between females and males. Clin Interv Aging. 2016;11:1309–16.
385.
Thangam M, Luke AA, Johnson DY, Amin AP, Lasala J, Huang K, et al. Sociodemographic differences in utilization and outcomes for temporary cardiovascular mechanical support in the setting of cardiogenic shock. Am Heart J. 2021 Jun;236:87–96.
386.
Toshima T, Hirayama A, Watanabe T, Goto J, Kobayashi Y, Otaki Y, et al. Unmet needs for emergency care and prevention of prehospital death in acute myocardial infarction. J Cardiol. 2021 Jun;77(6):605–12.
387.
Toyota T, Furukawa Y, Ehara N, Funakoshi S, Morimoto T, Kaji S, et al. Sex-based differences in clinical practice and outcomes for Japanese patients with acute myocardial infarction undergoing primary percutaneous coronary intervention. Circ J. 2013;77(6):1508–17.
388.
Tran VH, Mehawej J, Abboud DM, Tisminetzky M, Hariri E, Filippaios A, et al. Age and Sex Differences and Temporal Trends in the Use of Invasive and Noninvasive Procedures in Patients Hospitalized With Acute Myocardial Infarction. J Am Heart Assoc. 2022 Sep;11(17):e025605.
389.
Trent SA, George N, Havranek EP, Ginde AA, Haukoos JS. Established evidence-based treatment guidelines help mitigate disparities in quality of emergency care. Acad Emerg Med. 2021 Sep;28(9):1051–60.
390.
Tripathi B, Tan BE, Sharma P, Gaddam M, Singh A, Solanki D, et al. Characteristics and Outcomes of Patients Admitted With Type 2 Myocardial Infarction. Am J Cardiol. 2021 Oct;157:33–41.

391.
Tröbs M, Achenbach S, Plank PM, Marwan M, Röther J, Klinghammer L, et al. Predictors of Technical Failure in Transradial Coronary Angiography and Intervention. Am J Cardiol. 2017 Nov;120(9):1508–13.
392.
Truong QA, Hayden D, Woodard PK, Kirby R, Chou ET, Nagurney JT, et al. Sex differences in the effectiveness of early coronary computed tomographic angiography compared with standard emergency department evaluation for acute chest pain: the rule-out myocardial infarction with Computer-Assisted Tomography (ROMICAT)-II Trial. Circulation. 2013 Jun;127(25):2494–502.
393.
Udell JA, Fonarow GC, Maddox TM, Cannon CP, Frank Peacock W, Laskey WK, et al. Sustained sex-based treatment differences in acute coronary syndrome care: Insights from the American Heart Association Get With The Guidelines Coronary Artery Disease Registry. Clin Cardiol. 2018 Jun;41(6):758–68.
394.
Udell JA, Koh M, Qiu F, Austin PC, Wijeysundera HC, Bagai A, et al. Outcomes of Women and Men With Acute Coronary Syndrome Treated With and Without Percutaneous Coronary Revascularization. J Am Heart Assoc. 2017 Jan;6(1).
395.
Uemura S, Okamoto H, Nakai M, Nishimura K, Miyamoto Y, Yasuda S, et al. Primary Percutaneous Coronary Intervention in Elderly Patients With Acute Myocardial Infarction - An Analysis From a Japanese Nationwide Claim-Based Database. Circ J. 2019 May;83(6):1229–38.
396.
Vallabhajosyula S, Dunlay SM, Barsness GW, Elliott Miller P, Cheungpasitporn W, Stulak JM, et al. Sex Disparities in the Use and Outcomes of Temporary Mechanical Circulatory Support for Acute Myocardial Infarction-Cardiogenic Shock. CJC Open. 2020;2(6):462–72.
397.
Vallabhajosyula S, El Hajj SC, Bell MR, Prasad A, Lerman A, Rihal CS, et al. Intravascular ultrasound, optical coherence tomography, and fractional flow reserve use in acute myocardial infarction. Catheter Cardiovasc Interv. 2020 Jul;96(1):E59-e66.
398.
Vallabhajosyula S, Prasad A, Bell MR, S, hu GS, Eleid MF, et al. Extracorporeal Membrane Oxygenation Use in Acute Myocardial Infarction in the United States, 2000 to 2014. Circ Heart Fail. 2019 Dec;12(12):e005929.
399.
Vallabhajosyula S, Prasad A, Dunlay SM, Murphree Jr DH, Ingram C, Mueller PS, et al. Utilization of Palliative Care for Cardiogenic Shock Complicating Acute Myocardial Infarction: A 15-Year National Perspective on Trends, Disparities, Predictors, and Outcomes. J Am Heart Assoc. 2019 Aug;8(15):e011954.
400.
Vallabhajosyula S, Prasad A, S, hu GS, Bell MR, Gulati R, et al. Ten-year trends, predictors and outcomes of mechanical circulatory support in percutaneous coronary intervention for acute myocardial infarction with cardiogenic shock. EuroIntervention. 2021 Feb;16(15):e1254–61.
401.
Vallabhajosyula S, Shankar A, Patlolla SH, Prasad A, Bell MR, Jentzer JC, et al. Pulmonary artery catheter use in acute myocardial infarction-cardiogenic shock. ESC Heart Fail. 2020 Jun;7(3):1234–45.
402.
Vallabhajosyula S, Vallabhajosyula S, Dunlay SM, Hayes SN, Best PJM, Brenes-Salazar JA, et al. Sex and Gender Disparities in the Management and Outcomes of Acute Myocardial Infarction-Cardiogenic Shock in Older Adults. Mayo Clin Proc. 2020 Sep;95(9):1916–27.
403.
Vallabhajosyula S, Ya'Qoub L, Dunlay SM, Vallabhajosyula S, Vallabhajosyula S, Sundaragiri PR, et al. Sex disparities in acute kidney injury complicating acute myocardial infarction with cardiogenic shock. ESC Heart Fail. 2019 Aug;6(4):874–7.
404.

Vallabhajosyula S, Ya'Qoub L, Singh M, Bell MR, Gulati R, Cheungpasitporn W, et al. Sex Disparities in the Management and Outcomes of Cardiogenic Shock Complicating Acute Myocardial Infarction in the Young. Circ Heart Fail. 2020 Oct;13(10):e007154.
405.
Valle JA, Kaltenbach LA, Bradley SM, Yeh RW, Rao SV, Gurm HS, et al. Variation in the Adoption of Transradial Access for ST-Segment Elevation Myocardial Infarction: Insights From the NCDR CathPCI Registry. JACC Cardiovasc Interv. 2017 Nov;10(22):2242–54.
406.
van der Ende MY, Juarez-Orozco LE, Waardenburg I, Lipsic E, Schurer RAJ, van der Werf HW, et al. Sex-Based Differences in Unrecognized Myocardial Infarction. J Am Heart Assoc. 2020 Jul;9(13):e015519.
407.
van Engen-Verheul M, de Vries H, Kemps H, Kraaijenhagen R, de Keizer N, Peek N. Cardiac rehabilitation uptake and its determinants in the Netherlands. Eur J Prev Cardiol. 2013 Apr;20(2):349–56.
408.
Varma Y, Jena NK, Arsene C, Patel K, Sule AA, Krishnamoorthy G. Disparities in the management of non-ST-segment elevation myocardial infarction in the United States. Int J Cardiol. 2023 Jul;383:132–9.
409.
Vasiljevic Z, Krljanac G, Davidovic G, Panic G, Radovanovic S, Mickovski N, et al. Gender differences in case fatality rates of acute myocardial infarction in Serbia. European Heart Journal, Supplement. 2014;16:A48–55.
410.
Vasiljevic-Pokrajcic Z, Mickovski N, Davidovic G, Asanin M, Stefanovic B, Krljanac G, et al. Sex and age differences and outcomes in acute coronary syndromes. Int J Cardiol. 2016 Aug;217:S27-31.
411.
Velásquez-Rodríguez J, Vicent L, Díez-Delhoyo F, Valero Masa MJ, Bruña V, Sousa-Casasnovas I, et al. Prognostic Implications of High-Degree Atrio-Ventricular Block in Patients with Acute Myocardial Infarction in the Contemporary Era. Journal of Clinical Medicine [Internet]. 2023;12(14). Available from: https://www.embase.com/search/results?subaction=viewrecord&id=L2024746808&from=export http://dx.doi.org/10.3390/jcm12144834
412.
Velders MA, Boden H, van Boven AJ, van der Hoeven BL, Heestermans AA, Cannegieter SC, et al. Influence of gender on ischemic times and outcomes after ST-elevation myocardial infarction. Am J Cardiol. 2013 Feb;111(3):312–8.
413.
Ventura M, Belleudi V, Sciattella P, Di Domenicantonio R, Di Martino M, Agabiti N, et al. High quality process of care increases one-year survival after acute myocardial infarction (AMI): A cohort study in Italy. PLoS One. 2019;14(2):e0212398.
414.
Verghese D, Patlolla SH, Cheungpasitporn W, Doshi R, Miller VM, Jentzer JC, et al. Sex disparities in management and outcomes of cardiac arrest complicating acute myocardial infarction in the United States. Resuscitation. 2022 Mar;172:92–100.
415.
Wang JL, He XQ, Guo CY, Chen H, Li HW, Zhao SM. Sex-related differences in clinical outcomes and predictive factors in the very elderly patients with ACS undergoing PCI. Frontiers in Cardiovascular Medicine [Internet]. 2022;9. Available from: https://www.embase.com/search/results?subaction=viewrecord&id=L2019516866&from=export http://dx.doi.org/10.3389/fcvm.2022.950165
416.
Wang J, Yu W, Zhao D, Liu N, Yu Y. In-Hospital and Long-Term Mortality in 35,173 Chinese Patients Undergoing Coronary Artery Bypass Grafting in Beijing: Impact of Sex, Age, Myocardial Infarction, and Cardiopulmonary Bypass. J Cardiothorac Vasc Anesth. 2017 Feb;31(1):26–31.
417.

Wang S, Zhang Y, Cheng Q, Qi D, Wang X, Zhu Z, et al. Sex Disparity in Characteristics, Management, and In-Hospital Outcomes of Patients with ST-Segment Elevated Myocardial Infarction: Insights from Henan STEMI Registry. Cardiology Research and Practice [Internet]. 2022;2022. Available from: https://www.embase.com/search/results?subaction=viewrecord&id=L2020352859&from=export http://dx.doi.org/10.1155/2022/2835485
418.
Wang TKM, Grey C, Jiang Y, Jackson R, Kerr A. Trends in length of stay following acute coronary syndrome hospitalisation in New Zealand 2006-2016: ANZACS-QI 32 study. N Z Med J. 2020 Jan;133(1508):29–42.
419.
Wanha W, Kawecki D, Roleder T, Pluta A, Marcinkiewicz K, Morawiec B, et al. Gender differences and bleeding complications after PCI on first and second generation DES. Scand Cardiovasc J. 2017 Feb;51(1):53–60.
420.
Wei J, Mehta PK, Grey E, Garberich RF, Hauser R, Bairey Merz CN, et al. Sex-based differences in quality of care and outcomes in a health system using a standardized STEMI protocol. Am Heart J. 2017 Sep;191:30–6.
421.
Weizman O, Marijon E, Narayanan K, Boveda S, Defaye P, Martins R, et al. Incidence, Characteristics, and Outcomes of Ventricular Fibrillation Complicating Acute Myocardial Infarction in Women Admitted Alive in the Hospital. J Am Heart Assoc. 2022 Sep;11(17):e025959.
422.
Weizman O, Tea V, Marijon E, Eltchaninoff H, Manzo-Silberman S, Leclercq F, et al. Very long-term outcomes after acute myocardial infarction in young men and women: Insights from the FAST-MI program. Archives of Cardiovascular Diseases. 2023;116(6):324–34.
423.
Wen-Xia FU, Tie-Nan Z, Xiao-Zeng W, Lei Z, Quan-Min J, Ya-Ling HAN, et al. Sex-Related Differences in Short- and Long-Term Outcome among Young and Middle-Aged Patients for ST-Segment Elevation Myocardial Infarction Underwent Percutaneous Coronary Intervention. Chinese Medical Journal. 2018;(24):1420–9.
424.
Wenner JB, Wong GC, Cairns JA, Perry-Arnesen M, Tocher W, Mackay M, et al. Impact of Patient- and System-Level Delays on Reperfusion Among Patients With ST-Elevation Myocardial Infarction. CJC Open. 2020;2(3):94–103.
425.
Wibring K, Lingman M, Herlitz J, Blom L, Gripestam OS, Bång A. Guideline adherence among prehospital emergency nurses when caring for patients with chest pain: a prospective cohort study. Scand J Trauma Resusc Emerg Med. 2021 Oct;29(1):157.
426.
Wilkinson C, Bebb O, Dondo TB, Munyombwe T, Casadei B, Clarke S, et al. Sex differences in quality indicator attainment for myocardial infarction: a nationwide cohort study. Heart. 2019 Apr;105(7):516–23.
427.
Worrall-Carter L, MacIsaac A, Scruth E, Rahman MA. Gender difference in the use of coronary interventions for patients with acute coronary syndrome: Experience from a major metropolitan hospital in Melbourne, Australia. Aust Crit Care. 2017 Jan;30(1):3–10.
428.
Worrall-Carter L, McEvedy S, Wilson A, Rahman MA. Gender Differences in Presentation, Coronary Intervention, and Outcomes of 28,985 Acute Coronary Syndrome Patients in Victoria, Australia. Womens Health Issues. 2016 Jan;26(1):14–20.
429.
Worrall-Carter L, McEvedy S, Wilson A, Rahman MA. Impact of comorbidities and gender on the use of coronary interventions in patients with high-risk non-ST-segment elevation acute coronary syndrome. Catheter Cardiovasc Interv. 2016 Mar;87(4):E128-36.
430.
Wouters L, Zwart DLM, Erkelens DCA, De Groot E, van Smeden M, Hoes AW, et al. Gender-stratified analyses of symptoms associated with acute coronary syndrome in telephone triage: a cross-sectional study. BMJ Open. 2021 Jun;11(6):e042406.

431.
Xanthopoulou I, Davlouros P, Deftereos S, Hamilos M, Sitafidis G, Kanakakis I, et al. Gender-related differences in antiplatelet treatment patterns and outcome: Insights from the GReekAntiPlatElet Registry. Cardiovasc Ther. 2017 Aug;35(4).
432.
Xu M, Li HW, Chen H, Guo CY. Sex and Age Differences in Patients With Unstable Angina Pectoris: A Single-Center Retrospective Study. Am J Med Sci. 2020 Sep;360(3):268–78.
433.
Yang D, James S, de Faire U, Alfredsson L, Jernberg T, Moradi T. Likelihood of treatment in a coronary care unit for a first-time myocardial infarction in relation to sex, country of birth and socioeconomic position in Sweden. PLoS One. 2013;8(4):e62316.
434.
Ye G, Pattisapu VK, Wang P, Cheng L, Yao S, Hao H. Sex Differences and Temporal Trends in Revascularization and Outcomes of ST-Elevation Myocardial Infarction in Older Adults in the United States. Arch Med Res. 2022 Jun;53(4):441–50.
435.
Yoon CW, Oh H, Lee J, Rha JH, Woo SI, Lee WK, et al. Comparisons of Prehospital Delay and Related Factors Between Acute Ischemic Stroke and Acute Myocardial Infarction. J Am Heart Assoc. 2022 May;11(9):e023214.
436.
Yu J, Mehran R, Grinfeld L, Xu K, Nikolsky E, Brodie BR, et al. Sex-based differences in bleeding and long term adverse events after percutaneous coronary intervention for acute myocardial infarction: Three year results from the HORIZONS-AMI trial. Catheterization and Cardiovascular Interventions [Internet]. 2014; Available from: https://www.embase.com/search/results?subaction=viewrecord&id=L53320615&from=export
http://dx.doi.org/10.1002/ccd.25630
437.
Z, ecki L, Sadowski M, Janion M, Gierlotka M, Gasior M, et al. Trends in sex differences in clinical characteristics, treatment strategies, and mortality in patients with ST-elevation myocardial infarction in Poland from 2005 to 2011. Coron Artery Dis. 2017 Aug;28(5):417–25.
438.
Zachura M, Sadowski M, Kurzawski J, Piątek K, Gąsior M. Heterogeneity of the No-Reflow Group After Primary Percutaneous Coronary Intervention Due to ST-Segment Elevation Myocardial Infarction - Are There Sex Differences? Cardiovasc Revasc Med. 2022 Apr;37:97–101.
439.
Zachura M, Wilczek K, Kurzawski J, Gierlotka M, Gąsior M, Sadowski M. Gender-related differences in men and women with ST-segment elevation myocardial infarction and incomplete infarct-related artery flow restoration: A multicenter national registry. Postepy w Kardiologii Interwencyjnej. 2018;14(4):356–62.
440.
Zagnoni S, Casella G, Pallotti MG, Gonzini L, Abrignani MG, Caldarola P, et al. Sex differences in the management of acute coronary syndromes in Italy: data from the MANTRA registry. J Cardiovasc Med (Hagerstown). 2017 Mar;18(3):178–84.
441.
Zevallos JC, Yarzebski J, González JA, Banchs HL, García-Palmieri M, Mattei H, et al. Incidence, in-hospital case-fatality rates, and management practices in Puerto Ricans hospitalized with acute myocardial infarction. P R Health Sci J. 2013 Sep;32(3):138–45.
442.
Zhang B, Jiang DM, Zhou XC, Liu J, Zhang W, Sun YJ, et al. Prospective multi-center study of female patients with ST-elevation myocardial infarction in Liaoning province, China. Chin Med J (Engl). 2012 Jun;125(11):1915–9.
443.
Zhang B, Zhang W, Huang R, Zhu H, Liu J, Jiang D, et al. Gender and Age Differences Associated With Prehospital Delay in Chinese Patients Presenting With ST-Elevation Myocardial Infarction. J Cardiovasc Nurs. 2016 Mar;31(2):142–50.
444.

Zhang B, Zhang W, Huang RC, Zhang Y, Liu J, Zheng ZG, et al. Gender disparity in early death after ST-elevation myocardial infarction. Chin Med J (Engl). 2013;126(18):3481–5.
445.
Zheng H, Foo LL, Tan HC, Richards AM, Chan SP, Lee CH, et al. Sex Differences in 1-Year Rehospitalization for Heart Failure and Myocardial Infarction After Primary Percutaneous Coronary Intervention. Am J Cardiol. 2019 Jun;123(12):1935–40.
446.
Zheng X, Dreyer RP, Hu S, Spatz ES, Masoudi FA, Spertus JA, et al. Age-specific gender differences in early mortality following ST-segment elevation myocardial infarction in China. Heart. 2015 Mar;101(5):349–55.

**Supplementary Material 5. Characteristics of the included studies.**

| Title | First author | Publication year | GBD region |
|---|---|---|---|
| Sex differences in the management and outcomes of Ontario patients with cardiogenic shock complicating MI | Abdel-Qadir et al. | 2013 | high income |
| ACS in elderly – What is the place for invasive strategy? | Aguiar Rosa et al. | 2017 | high income |
| Gender differences in scene time, transport time, and total scene to hospital arrival Time determined by the use of a prehospital electrocardiogram in patients with complaint of Suspected ACS / Chest pain | Aguilar et al. | 2012 | high income |
| Relationship between risk factors and in-hospital Mortality due to myocardial infarction by educational level: a national prospective study in Iran | Ahmadi et al. | 2014 | north africa / middle east |
| Epidemiological pattern of myocardial infarction and modelling risk factors relevant to in-hospital mortality: the first results from the Iranian Myocardial Infarction Registry | Ahmadi et al. | 2015 | north africa / middle east |
| Predictive Factors of Hospital Mortality Due to Myocardial Infarction: A Multilevel Analysis of Iran's National Data | Ahmadi et al. | 2015 | north africa / middle east |
| Current status of the clinical epidemiology of myocardial infarction in men and women: a national cross-sectional study in Iran | Ahmadi et al. | 2015 | north africa / middle east |
| Assessment of coronary care management and hospital Mortality from ST-segment elevation myocardial infarction in the Kazakhstan population: Data from 2012 to 2015 | Akimbaeva et al. | 2017 | central europe / eastern europe / asia |
| Does prior coronary artery bypass surgery alter the gender gap in patients presenting with ACS? A 20-year retrospective cohort study | Al-Aqeedi et al. | 2012 | north africa / middle east |
| Contemporary outcomes in women undergoing percutaneous coronary intervention for ACS | Al-Fiadh et al. | 2011 | high income |
| Compliance with guideline-recommended management in patients with non-st-elevation ACS: Findings from the gulf COAST registry | Alajmi et al. | 2021 | north africa / middle east |
| Sex-Related Differences in Outcomes for Patients With ST Elevation Myocardial Infarction (STEMI): A TMII Nadu-STEMI Program Subgroup Analysis | Alexander et al. | 2021 | south asia |
| Incidence and Outcomes of Acute Ischemic Stroke Following Percutaneous Coronary Interventions in Men Versus Women | Alkhouli et al. | 2020 | high income |
| Age-Stratified Sex-Related Differences in the Incidence, Management, and Outcomes of MI | Alkhouli et al. | 2021 | high income |
| Contemporary Determinants of Delayed Benchmark Timelines in MI in Men and Women | Alnsasra et al. | 2017 | high income |
| Factors associated with emergency medical service delays in suspected ST-elevation myocardial infarction in Victoria, Australia: A retrospective study | Alrawashdeh et al. | 2020 | high income |
| Predictors of non-invasive therapy and 28-day-case fatality in elderly compared to younger patients with MI: an observational study from the MONICA/KORA Myocardial Infarction Registry | Amann et al. | 2016 | high income |
| Post-ACS Disparities in Guideline-Directed Lipid Therapy and Insufficient Achievement of Optimal Low-Density Lipoprotein | Ambrosino et al. | 2023 | high income |
| Incidence and Predictors of Adverse Events Among Initially Stable ST-Elevation Myocardial Infarction Patients Following Primary Percutaneous Coronary Intervention | Amon et al. | 2022 | high income |
| Sex-Based Difference in Clinical Presentation and Outcomes-A Single-Center Experience | Anandan et al. | 2021 | south asia |
| Sex-related inequalities in management of patients with ACS-results from the EURHOBOP study | Araújo et al. | 2018 | high income |
| Women with MI: Clinical Characteristics, Treatment, and In-Hospital Outcomes from a Latin American Country | Arias-Mendoza et al. | 2023 | latin america / caribbean |

| Title | First author | Publication year | GBD region |
|---|---|---|---|
| Sex-based differences in clinical outcomes and resource utilization of type 2 myocardial infarction | Ariss et al. | 2021 | high income |
| Twenty Year Trends and Sex Differences in Young Adults Hospitalized With MI | Arora et al. | 2019 | high income |
| Temporal trends in Mortality and provision of intensive care in younger women and men with MI or stroke | Arslani et al. | 2023 | high income |
| Sex differences of patients with acute Suspected ACS / Chest pain evaluated through a Suspected ACS / Chest pain unit | Arzuan et al. | 2023 | high income |
| Sex Disparities in Diagnosis Evaluation and Revascularization in Patients With MI-A 15-Year Nationwide Study | Ashraf et al. | 2023 | high income |
| ED triage of patients with MI: predictors of low acuity triage | Atzema et al. | 2010 | high income |
| Patient characteristics associated with Contact health services, Treatment delay and survival following primary percutaneous coronary intervention | Austin et al. | 2014 | high income |
| Femoral pseudoaneurysm in patients undergoing primary percutaneous coronary intervention for ST-elevation myocardial infarction: incidence, clinical course and risk factors | Ayhan et al. | 2012 | high income |
| Medical care of MI patients in a resource limiting country, Trinidad: a cross-sectional retrospective study | Bahall et al. | 2019 | latin america / caribbean |
| Ethnic and sex differences in ambulance activation among hospitalized patients with ACS: Insights from the Alberta contemporary ACS patients invasive Treatment strategies (COAPT) study | Bainey et al. | 2018 | high income |
| Treatment, outcomes, costs, and quality of life of women and men with ACS who have undergone percutaneous coronary intervention: results from the antiplatelet therapy observational registry | Bakhai et al. | 2013 | multiple |
| Identifying key factors leading to the optimal care pathway for patients with ST-segment elevation myocardial infarction: Results from the RESCMIP registry | Balen et al. | 2019 | high income |
| Sex and Race Differences in the Evaluation and Treatment of Young Adults Presenting to the Emergency Department With Suspected ACS / Chest pain | Banco et al. | 2022 | high income |
| MI in the young - National Trend Analysis with gender-based difference in outcomes | Bandyopadhyay et al. | 2020 | high income |
| Variation in Arterial Access for Invasive Coronary Procedures in New Zealand: A National Analysis (ANZACS-QI 5) | Barr et al. | 2016 | high income |
| Incidence and clinical impact of concurrent chronic total occlusion according to gender in ST-elevation myocardial infarction | Bataille et al. | 2013 | high income |
| Sex differences in cardiovascular outcomes, pharmacological treatments and indicators of care in patients with newly diagnosed diabetes: Analyses on administrative database | Baviera et al. | 2014 | high income |
| Longer Pre-hospital delays and higher Mortality in women with STEMI: the e-MUST Registry | Benamer et al. | 2016 | high income |
| Impact of Sex- and Gender-Related Factors on Length of Stay Following Non-ST-Segment-Elevation Myocardial Infarction: A Multicountry Analysis | Bender et al. | 2023 | high income |
| Revascularization Treatment of Emergency Patients with Acute ST-Segment Elevation Myocardial Infarction in Switzerland: Results from a Nationwide, Cross-Sectional Study in Switzerland for 2010-2011 | Berlin et al. | 2016 | high income |
| Influence of Total Ischemic Time on Clinical Outcomes in Patients with ST-Segment Elevation Myocardial Infarction | Bessonov et al. | 2021 | central europe / eastern europe / asia |
| Outcomes and Hospital Utilization in Patients With Papillary Muscle Rupture Associated With MI | Bhardwaj et al. | 2020 | high income |
| Disparities in Drug-Eluting Stent Utilization in Patients With Acute ST-Elevation Myocardial Infarction: An Analysis of the National Inpatient Sample | Bhasin et al. | 2023 | high income |

| Title | First author | Publication year | GBD region |
|---|---|---|---|
| Using decision trees for measuring gender equity in the timing of Angiography in patients with ACS: a novel approach to equity analysis | Bierman et al. | 2015 | high income |
| Do gender differences in primary PCI Mortality represent a different adherence to guideline recommended therapy? a multicenter observation | Birkemeyer et al. | 2014 | high income |
| Predictors of hospital prenotification for STEMI and association of prenotification with outcomes | Blusztein et al. | 2022 | high income |
| Prospective multi-center study of female patients with ST-elevation myocardial infarction in Liaoning province, China | Bo Zhang 1 et al. | 2012 | southeast asia / east asia / oceania |
| Mechanical Complications in ST-elevation myocardial infarction: The impact of Pre-hospital delay | Bouisset et al. | 2021 | multiple |
| Unexpected high level of severe Events even in low-risk profile Suspected ACS / Chest pain unit patients | Breuckmann et al. | 2022 | high income |
| Symptom-to-door times in patients presenting with ST elevation myocardial infarction--do ethnic or gender differences exist? | Brown et al. | 2016 | high income |
| Sex Differences in Symptom Complexity and Door-to-Balloon Time in Patients With ST-Elevation Myocardial Infarction | Brush et al. | 2023 | high income |
| Editor's Choice-Sex differences in young patients with MI: A VIRGO study analysis | Bucholz et al. | 2017 | high income |
| Delayed Care and Mortality Among Women and Men With Myocardial Infarction | Bugiardini et al. | 2017 | central europe / eastern europe / asia |
| Are hospital process quality indicators influenced by socio-demographic health determinants | Buja et al. | 2015 | high income |
| The choice of Treatment for myocardial infarction based on individual cardiovascular risk and symptoms of coronary heart disease | Buleshov et al. | 2019 | central europe / eastern europe / asia |
| Sex Differences in Outcome and Prescribing Practice in ST-elevation MI Patients with Multivessel Disease and Incomplete Revascularisation | Burgess et al. | 2023 | high income |
| Strategies to Lower In-Hospital Mortality in STEMI Patients with Primary PCI: Analysing Two Years Data from a High-Volume Interventional Centre | Burlacu et al. | 2019 | central europe / eastern europe / asia |
| Variation in the use of 12-lead ECG for patients with Suspected ACS / Chest pain by emergency medical services in North Carolina | Bush et al. | 2013 | high income |
| Access to percutaneous transluminal coronary angioplasty and 30-day Mortality in patients with incident STEMI: Differentials by educational level and gender over 11 years | Cacciani et al. | 2017 | high income |
| Predictors of In-hospital Mortality in Patients Presenting with ACS in a Tertiary Cardiac Center in Dhaka, Bangladesh | Cader et al. | 2022 | south asia |
| Potential Influential Factors of In-Hospital Myocardial Reinfarction in ST-Segment Elevation Myocardial Infarction (STEMI) Patients: Finding from the Improving Care for Cardiovascular Disease in China- (CCC-) ACS (ACS) Project | Cai et al. | 2021 | southeast asia / east asia / oceania |
| Time to reperfusion in high-risk patients with myocardial infarction undergoing primary percutaneous coronary intervention | Calé et al. | 2019 | high income |
| Effect of charted mental illness on reperfusion therapy in hospitalized patients with an MI in Florida | Campi et al. | 2017 | high income |
| Reperfusion Times for Radial Versus Femoral Access in Patients With ST-Elevation Myocardial Infarction Undergoing Primary Percutaneous Coronary Intervention: Observations From the Cardiac Care Network Provincial Primary PCI Registry | Cantor et al. | 2015 | high income |
| Predictors of primary percutaneous coronary intervention delay in cases of myocardial infarction diagnosed in hospitals without hemodynMIc support systems | Carol Ruiz et al. | 2021 | high income |

| Title | First author | Publication year | GBD region |
|---|---|---|---|
| Management of patients with ACS in real-world practice in Italy: an outcome research study focused on the use of ANTithRombotic Agents: the MANTRA registry | Casella et al. | 2013 | high income |
| Predictors of Hospital Mortality Based on Primary Angioplasty Treatment: A Multicenter Case-Control Study | Castro et al. | 2022 | latin america / caribbean |
| Reperfusion therapy for ST-elevation MI in Eastern Europe: The ISACS-TC registry | Cenko et al. | 2016 | central europe / eastern europe / asia |
| Sex-related differences in outcomes among men and women under 55 years of age with ACS undergoing percutaneous coronary intervention: Results from the PROMETHEUS study | Ch and rasekhar et al. | 2017 | high income |
| Clinical Outcomes in Patients With Delayed Hospitalization for Non-ST-Segment Elevation Myocardial Infarction | Cha et al. | 2022 | multiple |
| MI in the young with diabetes mellitus- national inpatient sample study with sex-based difference in outcomes | Chakraborty et al. | 2021 | high income |
| ST-segment Elevation Myocardial Infarction in North African Women: Results From a Twenty-year Experience | Chamtouri et al. | 2022 | north africa / middle east |
| Sex differences in long-term cardiovascular outcomes among patients with MI: A population-based retrospective cohort study | Chang et al. | 2021 | southeast asia / east asia / oceania |
| Predictors and long-term outcomes of in-hospital switching from clopidogrel to ticagrelor among patients with ACS undergoing percutaneous coronary intervention | Chen et al. | 2022 | high income |
| Insurance and Prehospital Delay in Patients ≤55 Years With MI | Chen et al. | 2015 | high income |
| Cardiac rehabilitation following an ACS: Trends in referral, predictors and Mortality outcome in a multicenter national registry between years 2006-2013: Report from the Working Group on Cardiac Rehabilitation, the Israeli Heart Society | Chernomordik et al. | 2017 | high income |
| Women were noninferior to men in cardiovascular outcomes among patients with ST-segment elevation myocardial infarction treated with primary percutaneous coronary intervention from Taiwan ACS full-spectrum registry | Chou et al. | 2018 | southeast asia / east asia / oceania |
| CHA2DS2-VASc score predicts contrast-induced nephropathy in patients with ST-segment elevation myocardial infarction, who have undergone primary percutaneous coronary intervention | Cicek et al. | 2018 | north africa / middle east |
| Diferencia de presentación del síndrome coronario agudo por género en pacientes llevados al laboratorio de cateterismo en población dominicana: un estudio retrospectivo | Colón Arias et al. | 2021 | latin america / caribbean |
| Eleven-year trends in gender differences of treatments and Mortality in ST-elevation MI in northern Italy, 2000 to 2010 | Corrada et al. | 2014 | latin america / caribbean |
| Myocardial infarction: sex differences in symptoms reported to emergency dispatch | Coventry et al. | 2013 | high income |
| Emergency Medical Services Time Intervals for Acute Suspected ACS / Chest pain in the United States, 2015-2016 | Cui et al. | 2020 | high income |
| Factors associated with use of percutaneous coronary intervention among elderly patients presenting with ST segment elevation MI (STEMI): Results from the ISACS-TC registry | Câlmâc et al. | 2016 | central europe / eastern europe / asia |
| Incidence and predictors of bleeding in ACS patients treated with PCI and prasugrel or ticagrelor: An analysis from the RENMI registry | D'Ascenzo et al. | 2018 | high income |
| Sex differences in reperfusion in young patients with ST-segment-elevation myocardial infarction: results from the VIRGO study | D'Onofrio et al. | 2015 | high income |
| Women Are Less Likely to Survive MI Presenting With Out-of-Hospital Cardiac Arrest: A Nationwide Study | Dafaalla et al. | 2022 | high income |
| Predictors of Radial to Femoral Artery Access Crossover During Primary Percutaneous Coronary Intervention for ST-Elevation Myocardial Infarction | Dang et al. | 2022 | high income |

| Title | First author | Publication year | GBD region |
|---|---|---|---|
| Sex Differences in Epidemiology, Care, and Outcomes in Patients With Acute Suspected ACS / Chest pain | Dawson et al. | 2023 | high income |
| Impact of time-to-Treatment on myocardial perfusion after primary percutaneous coronary intervention with Gp IIb-IIIa inhibitors | De Luca et al. | 2013 | multiple |
| Contemporary Trends and Age-Specific Sex Differences in Management and Outcome for Patients With ST-Segment Elevation Myocardial Infarction | De Luca et al. | 2016 | high income |
| Women who experience a myocardial infarction at a young age have worse outcomes compared with men: the Mass General Brigham YOUNG-MI registry | DeFilippis et al. | 2020 | high income |
| Medication Administration Delays in Non-ST Elevation Myocardial Infarction: Analysis of 1002 Patients Admitted to an Academic Medical Center | Denlinger et al. | 2018 | high income |
| Interhospital Transfer versus Direct Admission in Patients with Acute ST-Segment Elevation Myocardial Infarction | Dharma et al. | 2023 | southeast asia / east asia / oceania |
| Association of gender with clinical outcomes of patients with acute ST-segment elevation myocardial infarction presenting with acute heart failure | Dharma et al. | 2021 | southeast asia / east asia / oceania |
| In-hospital outcomes and long-term Mortality according to sex and management strategy in MI. Insights from the French ST-elevation and non-ST-elevation Myocardial Infarction (FAST-MI) 2005 Registry | Donataccio et al. | 2015 | high income |
| Sex Differences in Clinical Profiles and Quality of Care Among Patients With ST-Segment Elevation Myocardial Infarction From 2001 to 2011: Insights From the China Patient-Centered Evaluative Assessment of Cardiac Events(PEACE)-Retrospective Study | Du et al. | 2016 | southeast asia / east asia / oceania |
| Prognosis impact of diabetes in elderly women and men with non-st elevation ACS | Díez-Villanueva et al. | 2021 | high income |
| Acute kidney injury - A frequent and serious Complications after primary percutaneous coronary intervention in patients with ST-segment elevation myocardial infarction | El-Ahmadi et al. | 2019 | high income |
| Mortality trends in women and men presenting with ACS: insights from a 20-year registry | El-Menyar et al. | 2013 | north africa / middle east |
| Trends in Utilization and Safety of In-Hospital Coronary Artery Bypass Grafting During a Non-ST-Segment Elevation Myocardial Infarction | Elbaz-Greener et al. | 2020 | high income |
| Sex Differences in Management and Outcomes of MI Patients Presenting With Cardiogenic Shock | Elgendy et al. | 2022 | high income |
| Impact of hypertension on the outcome of patients admitted with ACS | Erne et al. | 2015 | high income |
| Outcome of patients admitted with ACS on palliative treatment: insights from the nationwide MIS Plus Registry 1997-2014 | Erne et al. | 2015 | high income |
| Is There a Sex Gap in Surviving an ACS or Subsequent Development of Heart Failure? | Ezekowitz et al. | 2020 | high income |
| ADVANCIS Score Predicts Acute Kidney Injury After Percutaneous Coronary Intervention for ACS | Fan et al. | 2018 | southeast asia / east asia / oceania |
| Gender gap in medical care in ST segment elevation myocardial infarction networks: Findings from the Catalan network Codi Infart | Fernández-Rodríguez et al. | 2017 | high income |
| Clinical profile and prognosis of young patients with ST-elevation myocardial infarction managed by the emergency-intervention Codi IAM network | Flores-Umanzor et al. | 2023 | high income |
| Reducing gap in Pre-hospital delay between women and men presenting with ST-elevation myocardial infarction | Foster-Witassek et al. | 2023 | high income |
| Nationwide Routine-Data Analysis of Sex Differences in Outcome of MI | Freisinger et al. | 2018 | high income |
| Relation of Gender to the Occurrence of AKI in STEMI Patients | Frydman et al. | 2022 | high income |

| Title | First author | Publication year | GBD region |
|---|---|---|---|
| Sex-Specific Clinical Characteristics and Long-Term Outcomes in Patients With Myocardial Infarction With Non-obstructive Coronary Arteries | Gao et al. | 2021 | southeast asia / east asia / oceania |
| Sex-based differences in clinical features, management, and 28-day and 7-year prognosis of first MI. RESCATE II study | García-García et al. | 2014 | high income |
| Long-term survival of Icelandic women following MI | Gardarsdottir et al. | 2022 | high income |
| Evidence-based prescribing of drugs for secondary prevention of ACS in Aboriginal and non-Aboriginal patients admitted to Western Australian hospitals | Gausia et al. | 2014 | high income |
| Sex differences in Time trends in ACS management and in 12-month lethality: Data from the French MONICA registries | Gauthier et al. | 2022 | high income |
| Renal dysfunction in STEMI-patients undergoing primary angioplasty: higher prevalence but equal prognostic impact in female patients; an observational cohort study from the Belgian STEMI registry | Gevaert et al. | 2014 | high income |
| Gender, TIMI risk score and in-hospital Mortality in STEMI patients undergoing primary PCI: results from the Belgian STEMI registry | Gevaert et al. | 2013 | high income |
| Gender disparities in ACS: a closing gap in the short-term outcome | Ghadri et al. | 2015 | high income |
| Is female gender associated with worse outcome after ST elevation myocardial infarction? | Ghaffari et al. | 2017 | north africa / middle east |
| Gender, socioeconomic position, revascularization procedures and Mortality in patients presenting with MI in the era of primary PCI. Differences or inequities? | Gnavi et al. | 2014 | high income |
| GRACE risk score: Sex-based validity of in-hospital Mortality prediction in Canadian patients with ACS | Gong et al. | 2017 | high income |
| Patient-physician gender concordance and increased Mortality among female heart attack patients | Greenwood et al. | 2018 | high income |
| Gender differences in Angiography, subsequent interventions, and outcomes among patients with ACS | Gudnadottir et al. | 2017 | high income |
| Sex Differences in Characteristics, Treatments, and Outcomes Among Patients Hospitalized for Non-ST-Segment-Elevation Myocardial Infarction in China: 2006 to 2015 | Guo et al. | 2022 | southeast asia / east asia / oceania |
| Sex Differences in Timeliness of Reperfusion in Young Patients With ST-Segment-Elevation Myocardial Infarction by Initial Electrocardiographic Characteristics | Gupta et al. | 2018 | high income |
| Frequency and Effects of Excess Dosing of Anticoagulants in Patients ≤55 Years With MI Who Underwent Percutaneous Coronary Intervention (from the VIRGO Study) | Gupta et al. | 2015 | high income |
| Contemporary Sex-Based Differences by Age in Presenting Characteristics, Use of an Early Invasive Strategy, and Inhospital Mortality in Patients With Non-ST-Segment-Elevation Myocardial Infarction in the United States | Gupta et al. | 2018 | high income |
| Comfort Measures Only in Myocardial Infarction: Prevalence of This Status, Change Over Time, and Predictors From a Nationwide Study | Haghighat et al. | 2022 | high income |
| Sex differences in the Treatment and outcomes of patients hospitalized with ST-elevation myocardial infarction | Hannan et al. | 2020 | high income |
| Developments in the invasive Diagnosis-therapeutic cascade of women and men with ACS from 2005 to 2011: a nationwide cohort study | Hansen et al. | 2015 | high income |
| Clinical Characteristics of Patients With MI Who Did Not Undergo Primary Percutaneous Coronary Intervention- Report From the MIYAGI-MI Registry Study | Hao et al. | 2015 | high income |
| Sex Differences in In-Hospital Management and Outcomes of Patients With ACS | Hao et al. | 2019 | southeast asia / east asia / oceania |

| Title | First author | Publication year | GBD region |
|---|---|---|---|
| A real-world analysis of cardiac rupture on incidence, risk factors and in-hospital outcomes in 4190 ST-elevation myocardial infarction patients from 2004 to 2015 | Hao et al. | 2020 | southeast asia / east asia / oceania |
| The outcome of ST-elevation myocardial infarction by sex: a retrospective cohort study | Hassani et al. | 2023 | north africa / middle east |
| Gender differences in therapeutic recommendation after Diagnosis Angiography: insights from the Angiography and PCI Registry of the German Society of Cardiology | Heer et al. | 2015 | high income |
| Sex Differences in Percutaneous Coronary Intervention-Insights From the Angiography and PCI Registry of the German Society of Cardiology | Heer et al. | 2017 | high income |
| Sex-related differences in the management and outcomes of patients hospitalized with ST-elevation myocardial infarction: a comparison within four European myocardial infarction registries | Hellgren et al. | 2022 | multiple |
| The contribution of gender and age on early and late Mortality following ST-segment elevation myocardial infarction: results from the Korean MI National Registry with Registries | Her et al. | 2018 | multiple |
| Epidemiological Profile of Hispanics Admitted With MI in Puerto Rico: The Experience of 2007, 2009 and 2011 | Hernandez-Suarez et al. | 2017 | latin america / caribbean |
| Sex differences in crude Mortality rates and predictive value of intensive care unit-based scores when applied to the cardiac intensive care unit | Herscovici et al. | 2020 | high income |
| Gender inequality in the clinical outcomes of equally treated ACS patients in Saudi Arabia | Hersi et al. | 2013 | north africa / middle east |
| Sex-based differences in outcomes after percutaneous coronary intervention for MI: a report from TRANSLATE-ACS | Hess et al. | 2014 | high income |
| Disparities by sex in P2Y(12) inhibitor therapy duration, or differences in the balance of ischaemic-benefit and bleeding-risk clinical outcomes in older women versus comparable men following MI? A P2Y(12) inhibitor new user retrospective cohort analysis of US Medicare claims data | Hickson et al. | 2021 | high income |
| Gender-specific uncertainties in the Diagnosis of ACS | Hillinger et al. | 2017 | multiple |
| Impact of Regional Systems of Care on Disparities in Care Among Female and Black Patients Presenting With ST-Segment-Elevation Myocardial Infarction | Hinohara et al. | 2017 | high income |
| Optimal Medical Therapy Medication in Patients with ACS in the Netherlands: A Multicenter Pilot Registry | Hoedemaker et al. | 2021 | high income |
| Editor's Choice- Gender difference in prognostic impact of in-hospital bleeding after myocardial infarction - data from the SWEDEHEART registry | Holm et al. | 2016 | high income |
| Trends in the clinical and pathological characteristics of cardiac rupture in patients with MI over 35 years | Honda et al. | 2014 | high income |
| Sex Differences in the Treatment and Outcome of Korean Patients With MI Using the Korean National Health Insurance Claims Database | Hong et al. | 2015 | multiple |
| Sex differences in emergency medical services management of patients with myocardial infarction: analysis of routinely collected data for over 110,000 patients | Hsu et al. | 2021 | high income |
| Pre-hospital delay in patients with MI in China: findings from the Improving Care for Cardiovascular Disease in China-ACS (CCC-ACS) project | Hu et al. | 2022 | southeast asia / east asia / oceania |
| Gender disparities in MI management and outcomes among health professionals, their relatives, and non-health professionals in Taiwan from 1997 to 2007 | Huang et al. | 2013 | southeast asia / east asia / oceania |
| Sex related inequalities in the management and prognosis of ACS in Switzerland: cross sectional study | Huber et al. | 2022 | high income |
| 4-Step Protocol for Disparities in STEMI Care and Outcomes in Women | Huded et al. | 2018 | high income |

| Title | First author | Publication year | GBD region |
|---|---|---|---|
| Five years of a comprehensive ST-elevation myocardial infarction protocol and its association with sex disparities | Huded et al. | 2021 | high income |
| Sex Differences in Cardiac Biomarkers Testing in Patients Presenting to the Emergency Department with Suspected ACS / Chest pain | Humphries et al. | 2018 | high income |
| Characteristics and outcomes in MI hospitalizations among the older population (age ≥80 years) in the United States, 2004-2018 | Ijaz et al. | 2023 | high income |
| Performance of the Academic Research Consortium High Bleeding Risk Criteria in Patients With ST-Segment Elevation Myocardial Infarction: A Single Center Study | Inan et al. | 2022 | north africa / middle east |
| Impact of gender on use of revascularization in ACS: the national observational study of Diagnosis and interventional cardiac Angiography(ONACI) | Isorni et al. | 2015 | high income |
| Gender disparity in the use of drug-eluting stents during percutaneous coronary intervention for MI | Iyanoye et al. | 2015 | high income |
| Do younger women fare worse? Sex differences in MI hospitalization and early Mortality rates over ten years | Izadnegahdar et al. | 2014 | high income |
| Mortality of patients with ST-segment-elevation myocardial infarction without standard modifiable risk factors among patients without known CAD: Age-stratified and sex-related analysis from nationwide readmissions database 2010-2014 | Jang et al. | 2023 | high income |
| Creatinine clearance is key to solving the enigma of sex difference in in-hospital Mortality after STEMI: Propensity score matching and Medication analysis | Janjani et al. | 2023 | north africa / middle east |
| Dyslipidemia management in patients with CAD. Data from the POLASPIRE survey | Jankowski et al. | 2021 | central europe / eastern europe / asia |
| Gender differences in risk profile and outcome of Middle Eastern patients undergoing percutaneous coronary intervention | Jarrah et al. | 2017 | north africa / middle east |
| Do clinical factors explain persistent sex disparities in the use of acute reperfusion therapy in STEMI in Sweden and Canada? | Johnston et al. | 2013 | high income |
| Effect of Gender on Patients With ST-Elevation and Non-ST-Elevation Myocardial Infarction Without Obstructive CAD | Johnston et al. | 2015 | high income |
| Gender differences in the assessment and Treatment of myocardial infarction | Jortveit et al. | 2016 | high income |
| Gender is Not a Predictor of Mortality or Major Adverse Cardiovascular Events in Patients Undergoing Percutaneous Coronary Intervention for ACS | Josiah et al. | 2019 | high income |
| Comparison of hospital Mortality during ST-segment elevation myocardial infarction in the era of reperfusion therapy in women versus men and in older versus younger patients | Juliard et al. | 2013 | high income |
| Does Gender Have Prognostic Value Among Patients with Myocardial Infarction? Analysis of the Data from the Hungarian Myocardial Infarction Registry | Jánosi et al. | 2018 | central europe / eastern europe / asia |
| Women and acute kidney injury in myocardial infarction | Kanic et al. | 2018 | central europe / eastern europe / asia |
| Gender Related Survival Differences in ST-Elevation Myocardial Infarction Patients Treated with Primary PCI | Kanic et al. | 2016 | central europe / eastern europe / asia |
| Association between bleeding and Mortality among women and men with high-risk ACS: insights from the Early versus Delayed, Provisional Eptifibatide in ACS (EARLY ACS) trial | Kaul et al. | 2013 | multiple |
| Less revascularization in young women but impaired long-term outcomes in young men after myocardial infarction | Kerola et al. | 2022 | high income |
| Sex Differences in Cardiovascular Outcomes of Older Adults After Myocardial Infarction | Kerola et al. | 2021 | high income |

| Title | First author | Publication year | GBD region |
|---|---|---|---|
| Epidemiological variation of MI relevant to in-hospital outcomes-tertiary center experience-saudi arabia | Khaled et al. | 2020 | north africa / middle east |
| Clinical Characteristics and Short-Term Outcomes of Patients Presenting with MI having Multi-vessel disease - A Single Middle- eastern Tertiary-Care Center Experience | Khaled et al. | 2022 | north africa / middle east |
| Differences in management and outcomes for men and women with ST-elevation myocardial infarction | Khan et al. | 2018 | high income |
| Trends, Outcomes, and Predictors of Revascularization in Cardiogenic Shock | Khan et al. | 2020 | high income |
| Sex differences in prodromal symptoms in ACS in patients aged 55_years or younger | Khan et al. | 2017 | high income |
| Temporal Trends and Sex Differences in Revascularization and Outcomes of ST-Segment Elevation Myocardial Infarction in Younger Adults in the United States | Khera et al. | 2015 | multiple |
| ST-elevation myocardial infarction in the elderly--temporal trends in incidence, utilization of percutaneous coronary intervention and outcomes in the United States | Khera et al. | 2013 | multiple |
| Gender Differences among Patients with ACS in the Middle East | Khesroh et al. | 2017 | north africa / middle east |
| Sex disparities in the presentation, management and outcomes of patients with ACS: insights from the ACSQUIK trial | Khraishah et al. | 2021 | south asia |
| Clinical characteristics and cardiovascular outcomes among young patients with MI in Kerala, India: A secondary analysis of ACSQUIK trial | Khraishah et al. | 2022 | south asia |
| Short and Midterm Outcomes in Patients With MI: Results of the Nationwide TURKMI Registry | Kilickap et al. | 2021 | north africa / middle east |
| Contemporary Trends in Oral Antiplatelet Agent Use in Patients Treated with Percutaneous Coronary Intervention for ACS | Kim et al. | 2017 | multiple |
| Gender disparities in prevalence by Diagnosis criteria, Treatment and Mortality of newly diagnosed MI in Korean adults | Kim et al. | 2023 | multiple |
| Sex Differences in Delayed Hospitalization in Patients with Non-ST-Segment Elevation Myocardial Infarction Undergoing New-Generation Drug-Eluting Stent Implantation | Kim et al. | 2023 | multiple |
| Sex differences in investigations and outcomes among patients with type 2 myocardial infarction | Kimenai et al. | 2021 | high income |
| Coronary perforation complicating percutaneous coronary intervention in patients presenting with an ACS: An analysis of 1013 perforation cases from the British Cardiovascular Intervention Society database | Kinnaird et al. | 2020 | high income |
| Lipid-lowering pharmacotherapy and socioeconomic status: Atherosclerosis Risk In Communities (ARIC) surveillance study | Kitzmiller et al. | 2013 | high income |
| Radial versus femoral access, bleeding and ischemic Events in patients with non-ST-segment elevation ACS managed with an invasive strategy | Klutstein et al. | 2013 | multiple |
| Mortality of women with ST-segment elevation myocardial infarction and cardiogenic shock - Results from the PL-ACS registry | Ko_odziej et al. | 2016 | central europe / eastern europe / asia |
| Female sex as an independent predictor of high bleeding risk among East Asian percutaneous coronary intervention patients: A sex difference analysis | Kodaira et al. | 2021 | high income |
| Hospital stage of myocardial infarction Treatment in 13 regions of Russian Federation by results of the international research | Kontsevaya et al. | 2018 | central europe / eastern europe / asia |
| Persisting gender differences and attenuating age differences in cardiovascular drug use for prevention and Treatment of coronary heart disease, 1998-2010 | Koopman et al. | 2013 | high income |
| Infarct size, left ventricular function, and prognosis in women compared to men after primary percutaneous coronary intervention in ST-segment elevation myocardial infarction: results from an individual patient-level pooled analysis of 10 randomized trials | Kosmidou et al. | 2017 | multiple |

| Title | First author | Publication year | GBD region |
|---|---|---|---|
| Clinical outcomes following primary percutaneous coronary intervention for ST-elevation myocardial infarction according to sex and race | Krishnamurthy et al. | 2019 | high income |
| Trends in Incidence, Characteristics, and In-Hospital Outcomes of Patients Presenting With Spontaneous Coronary Artery Dissection (From a National Population-Based Cohort Study Between 2004 and 2015) | Krishnamurthy et al. | 2019 | high income |
| Gender differences in MI-A nationwide German real-life analysis from 2014 to 2017 | Kuehnemund et al. | 2021 | high income |
| Sex disparities in guideline-recommended therapies and outcomes after ST-elevation myocardial infarction in a contemporary nationwide cohort of patients over an eight-year period | Kuehnemund et al. | 2023 | high income |
| Gender difference in Treatment and Mortality of patients with ST-segment elevation myocardial infarction admitted to Victorian public hospitals: a retrospective database study | Kuhn et al. | 2015 | high income |
| Factors associated with delayed Treatment onset for MI in Victorian emergency departments: a regression tree analysis | Kuhn et al. | 2013 | high income |
| Gender comparisons in cardiogenic shock during ST elevation myocardial infarction treated by primary percutaneous coronary intervention | Kunadian et al. | 2013 | high income |
| Bilateral internal mammary artery grafting reverses the negative influence of gender on outcomes of coronary artery bypass grafting surgery | Kurlansky et al. | 2013 | multiple |
| Effect of access site, gender, and indication on clinical outcomes after percutaneous coronary intervention: Insights from the British Cardiovascular Intervention Society (BCIS) | Kwok et al. | 2015 | high income |
| Usage of PCI and long-term cardiovascular risk in post-myocardial infarction patients: a nationwide registry cohort study from Finland | Kytö et al. | 2019 | high income |
| Gender and in-hospital Mortality of ST-segment elevation myocardial infarction (from a multihospital nationwide registry study of 31,689 patients) | Kytö et al. | 2015 | high income |
| Sex Differences in Outcomes Following ACS Treated With Coronary Artery Bypass Surgery | Kytö et al. | 2021 | high income |
| Sex differences in clinical characteristics and outcomes after myocardial infarction: insights from the Valsartan in MI Trial (VALIANT) | Lam et al. | 2015 | high income |
| Transfer of patients with ST-elevation myocardial infarction for primary percutaneous coronary intervention: a province-wide evaluation of "door-in to door-out" delays at the first hospital | Lambert et al. | 2014 | high income |
| Gender-based outcome differences for emergency department presentation of non-STEMIACS | Langabeer et al. | 2019 | multiple |
| Sex-Based Differences in Discharge Disposition and Outcomes for ST-Segment Elevation Myocardial Infarction Patients Within a Regional Network | Langabeer et al. | 2018 | high income |
| Decision to deploy coronary reperfusion is not affected by the volume of ST-segment elevation myocardial infarction patients managed by prehospital emergency medical teams | Lapostolle et al. | 2019 | high income |
| Effect of age, gender, and Time of day on pain-to-call times in patients with acute ST-segment elevation myocardial infarction: the CLOC’AGE study | Lapostolle et al. | 2021 | high income |
| Gender-related Mortality and in-hospital Complications following ST-segment elevation myocardial infarction: data from a primary percutaneous coronary intervention cohort | Laufer-Perl et al. | 2015 | multiple |
| Gender differences in hospital admissions for major cardiovascular Events and procedures in people with and without diabetes in England: a nationwide study 2004-2014 | Laverty et al. | 2017 | high income |
| A gender perspective on short- and long term Mortality in ST-elevation myocardial infarction--a report from the SWEDEHEART register | Lawesson et al. | 2013 | high income |
| Sex differences in Treatment and outcomes amongst myocardial infarction patients presenting with and without obstructive coronary arteries: a prospective multicentre study | Lawless et al. | 2023 | high income |
| Sex Differences in Outcomes of Ticagrelor Therapy With or Without Aspirin After Percutaneous Coronary Intervention in Patients With ACS: A Post Hoc Secondary Analysis of the TICO Randomized Clinical Trial | Lee et al. | 2023 | multiple |

| Title | First author | Publication year | GBD region |
|---|---|---|---|
| Are there gender differences in CAD? The Malaysian National Cardiovascular Disease Database - Percutaneous Coronary Intervention (NCVD-PCI) Registry | Lee et al. | 2013 | southeast asia / east asia / oceania |
| Sex and gender differences in presentation, Treatment and outcomes in ACS, a 10 year study from a multi-ethnic Asian population: The Malaysian National Cardiovascular Disease Database-ACS (NCVD-ACS) registry | Lee et al. | 2022 | southeast asia / east asia / oceania |
| Contemporary Trends of Optimal Evidence-Based Medical Therapy at Discharge for Patients Surviving MI From the Korea MI Registry | Lee et al. | 2015 | multiple |
| Sex-Specific Thresholds of High-Sensitivity Biomarkers in Patients With Suspected ACS / Suspected ACS / Chest pain | Lee et al. | 2019 | high income |
| Gender differences in clinical outcomes of MI undergoing percutaneous coronary intervention: Insights from the KMIR-NIH Registry | Lee et al. | 2020 | multiple |
| Pre-hospital delay and emergency medical services in MI | Lee et al. | 2020 | multiple |
| Rates and impact of vascular Complications in mechanical circulatory support | Lemor et al. | 2022 | high income |
| Impact of gender difference in hospital outcomes following percutaneous coronary intervention. Results of the Belgian Working Group on Interventional Cardiology (BWGIC) registry | Lempereur et al. | 2016 | high income |
| Contemporary invasive management and in-hospital outcomes of patients with non-ST-segment elevation myocardial infarction in China: Findings from China MI (CMI) Registry | Leng et al. | 2019 | southeast asia / east asia / oceania |
| Gender differences in presentation, management and inhospital outcome in patients with ST-segment elevation myocardial infarction: data from 5000 patients included in the ORBI prospective French regional registry | Leurent et al. | 2014 | high income |
| Gender differences in use of invasive Diagnosis and therapeutic procedures for acute ischaemic heart disease in Chinese adults | Levy et al. | 2022 | southeast asia / east asia / oceania |
| A Novel Risk Score to Predict In-Hospital Mortality in Patients With MI: Results From a Prospective Observational Cohort | Li et al. | 2022 | southeast asia / east asia / oceania |
| Diagnosis Model of In-Hospital Mortality in Patients with Acute ST-Segment Elevation Myocardial Infarction Used Artificial Intelligence Methods | Li et al. | 2022 | multiple |
| Population-based multilevel models to estimate the management strategies for MI in older adults with dementia | Li et al. | 2021 | high income |
| Surgical revascularization for ACS: a report from the North Rhine-Westphalia surgical myocardial infarction registry | Liakopoulos et al. | 2020 | high income |
| Sex Differences in the Presentation and Perception of Symptoms Among Young Patients With Myocardial Infarction: Evidence from the VIRGO Study (Variation in Recovery: Role of Gender on Outcomes of Young MI Patients) | Lichtman et al. | 2018 | multiple |
| Sex differences in the Treatment and outcome of patients with ACS after percutaneous coronary intervention: a population-based study | Lin et al. | 2014 | southeast asia / east asia / oceania |
| Sex differences following percutaneous coronary intervention or coronary artery bypass surgery for MI | Lin et al. | 2022 | southeast asia / east asia / oceania |
| Differences Between Women and Men in Phase I Cardiac Rehabilitation After MI: A Nationwide Population-Based Analysis | Lin et al. | 2016 | southeast asia / east asia / oceania |
| Prevalence and management of hypertension in patients with ACS vary with gender: Observations from the Chinese registry of acute coronary Events(CRACE) | Lin et al. | 2013 | southeast asia / east asia / oceania |

| Title | First author | Publication year | GBD region |
|---|---|---|---|
| Associated factors with repeat coronary angioplasty during the drug eluting stent era: a high volume center investigation | Liu et al. | 2013 | southeast asia / east asia / oceania |
| Age-Stratified Sex Disparities in Care and Outcomes in Patients With ST-Elevation Myocardial Infarction | Liu et al. | 2020 | multiple |
| Are there sex differences in the effect of type 2 diabetes in the incidence and outcomes of myocardial infarction? A matched-pair analysis using hospital Discharge data | Lopez-de-Andres et al. | 2021 | high income |
| Sex differences in ACS in a multiethnic asian population: results of the malaysian national cardiovascular disease database-ACS (NCVD-ACS) registry | Lu et al. | 2014 | southeast asia / east asia / oceania |
| Sex differences in lipid profiles and Treatment utilization among young adults with MI: Results from the VIRGO study | Lu et al. | 2017 | multiple |
| Clinical Characteristics, Prognosis, and Gender Disparities in Young Patients With MI | Lv et al. | 2021 | southeast asia / east asia / oceania |
| Clinical characteristics and in-hospital evolution of women with MI in the SAC-FAC National Registry of Infarction | Macín et al. | 2018 | high income |
| Gender differences in the management of ACS patients: One year results from HPIAR (HP-India ACSRegistry) | Mahajan et al. | 2017 | south asia |
| Gender Impact on 30-Day Readmissions After Hospitalization With MI Complicated by Cardiogenic Shock (from the 2013 to 2014 National Readmissions Database) | Mahmoud et al. | 2018 | multiple |
| Comparison of Outcomes of Coronary Revascularization for MI in Men Versus Women | Mahowald et al. | 2020 | multiple |
| Higher in-hospital Mortality during weekend admission for ACS: a large-scale cross-sectional Italian study | Malanchini et al. | 2019 | high income |
| UA: Trends and characteristics associated with length of hospitalization in the face of diminishing frequency—an evidence of a paradigm shift | Malik et al. | 2018 | high income |
| Influence of gender on delays and early Mortality in ST-segment elevation myocardial infarction: Insight from the first French Metaregistry, 2005-2012 patient-level pooled analysis | Manzo-Silberman et al. | 2018 | high income |
| Sex Disparities in First Medical Contact of Patients with Suspected ACS / Suspected ACS / Chest pain Using Telemedicine Technology | Maor et al. | 2020 | high income |
| Trends and predictors of prehospital delay in patients undergoing primary coronary intervention | Margolis et al. | 2018 | high income |
| Gender differences in the Treatment of ACS: Results of the Epi-Cardio registry | Mariani et al. | 2013 | high income |
| Trends of Sex Differences in Clinical Outcomes After Myocardial Infarction in the United States | Matetic et al. | 2021 | multiple |
| Sex and Medium-term Outcomes of ST-Segment Elevation Myocardial Infarction in Kerala, India: A Propensity Score–Matched Analysis | Mathew et al. | 2021 | south asia |
| Complications in STEMI(ST segment elevation myocardial infarction) due to BMI (Body Mass Index) | McDonald et al. | 2020 | high income |
| Sex and Gender Equity in Prehospital Electrocardiogram Acquisition | McDonald et al. | 2022 | high income |
| Gender differences in patient and system delay for primary percutaneous coronary intervention: current trends in a Swiss ST-segment elevation myocardial infarction population | Meyer et al. | 2019 | high income |
| In-hospital Mortality of patients with MI before and after introduction of PCI in Split University Hospital Center, Croatia | Miri_ et al. | 2013 | central europe / eastern europe / asia |
| Sex differences in in-hospital Mortality following a first MI: symptomatology, delayed presentation, and hospital setting | Mnatzaganian et al. | 2016 | high income |

| Title | First author | Publication year | GBD region |
|---|---|---|---|
| Socioeconomic gradients in admission to coronary or intensive care units among Australians presenting with non-traumatic Suspected ACS / Chest pain in emergency departments | Mnatzaganian et al. | 2018 | high income |
| MI in Severe Mental Illness: Prevalence, Clinical Outcomes, and Process of Care in U.S. Hospitalizations | Mohamed et al. | 2019 | multiple |
| Trends in Inequities in the Treatment of and Outcomes for Women and Minorities with Myocardial Infarction | Montoy et al. | 2022 | high income |
| Treatment gaps, 1-year readmission and Mortality following myocardial infarction by diabetes status, sex and socioeconomic disadvantage | Morton et al. | 2022 | high income |
| Gradual decline in the age-adjusted in-hospital Mortality rate from STEMI-related cardiogenic shock irrespective of cause, race or gender with persistent higher Mortality rates in women despite multivariate adjustment | Movahed et al. | 2014 | multiple |
| Impact of Gender and Door-to-Balloon Times on Long-Term Mortality in Patients Presenting With ST-Elevation Myocardial Infarction | Murphy et al. | 2019 | high income |
| Sex-Related Differences in In-Hospital Mortality in Japanese ST-Elevation MI Patients Presenting to Hospital in the 24 Hours After Symptom Onset_- Results From K-ACTIVE | Nagumo et al. | 2019 | high income |
| Sex differences in prehospital analgesia in patients presenting with ACS and their association with clinical outcomes | Nan Tie et al. | 2022 | high income |
| Sex-Based Differences in Presentation, Treatment, and Complications Among Older Adults Hospitalized for MI: The SILVER-MI Study | Nanna et al. | 2019 | high income |
| Las mujeres jóvenes en Chile tienen elevado riesgo de muerte intrahospitalaria por infarto de miocardio | Nazzal et al. | 2013 | high income |
| Sex-specific outcomes in patients with ACS | Neumann et al. | 2020 | high income |
| An Asian Perspective on Gender Differences in In-Hospital and Long-Term Outcome of Cardiac Mortality and Ischemic Stroke after Primary Percutaneous Coronary Intervention for ST-Segment Elevation Myocardial Infarction | Ngiam et al. | 2022 | high income |
| Patient delay in patients with ST-elevation myocardial infarction: Time patterns and predictors for a prolonged delay | Nielsen et al. | 2017 | high income |
| Sex-specific differences in risk factors for in-hospital Mortality and Complications in patients with ACS : An observational cohort study | Novak et al. | 2017 | central europe / eastern europe / asia |
| Comparison of Outcomes of Women Versus Men With Non-ST-elevation ACS Undergoing Percutaneous Coronary Intervention (from the Japanese Nationwide Registry) | Numasawa et al. | 2017 | high income |
| The Impact of Gender and Race When Using the GRACE ACScore to Predict Mortality | Ogbu et al. | 2023 | high income |
| Sex differences in the contemporary management of HIV patients admitted for MI | Ogunbayo et al. | 2018 | multiple |
| Changes in Periprocedural Bleeding Complications Following Percutaneous Coronary Intervention in The United Kingdom Between 2006 and 2013 (from the British Cardiovascular Interventional Society) | Olier et al. | 2018 | high income |
| Gender-based outcomes of coronary bifurcation stenting: A report from the National Readmission Database | Osman et al. | 2022 | multiple |
| Age stratified sex-related differences in incidence, management, and outcomes of cardiogenic shock | Osman et al. | 2022 | multiple |
| Is the difference in outcome between men and women treated by primary percutaneous coronary intervention age dependent? Gender difference in STEMI stratified on age | Otten et al. | 2013 | high income |
| Prehospital Delay in Older Adults with MI: The ComprehenSIVe Evaluation of Risk Factors in Older Patients with MI Study | Ouellet et al. | 2017 | high income |
| Revascularization Trends in Patients With Diabetes Mellitus and Multivessel CAD Presenting With Non-ST Elevation Myocardial Infarction: Insights From the National Cardiovascular Data | P and ey et al. | 2016 | multiple |

| Title | First author | Publication year | GBD region |
|---|---|---|---|
| Registry Acute Coronary Treatment and Intervention Outcomes Network Registry-Get with the Guidelines (NCDR ACTION Registry-GWTG) | | | |
| Association between gender, process of care measures, and outcomes in ACS in India: results from the detection and management of coronary heart disease (DEMAT) registry | Pagidipati et al. | 2013 | south asia |
| Radial Versus Femoral Access for Angiography/Intervention in Women With ACS: Insights From the RIVAL Trial (Radial Vs femorAL access for coronary intervention) | Pandie et al. | 2015 | high income |
| Sex-related impacts on clinical outcomes after percutaneous coronary intervention | Park et al. | 2020 | multiple |
| Variation in Treatment strategy for non-ST segment elevation myocardial infarction: A multilevel methodological approach | Park et al. | 2021 | multiple |
| Sex Differences in the Presentation, Diagnosis, and Management of ACS: Findings From the Kerala-India ACSRegistry | Patel et al. | 2015 | south asia |
| Impact of Gender, Race, and Insurance Status on Inhospital Management and Outcomes in Patients With COVID-19 and ST-Elevation Myocardial Infarction (a Nationwide Analysis) | Patel et al. | 2023 | high income |
| Outcomes of hospitalized patients with myocardial infarction and immune thrombocytopenic purpura: A cross sectional study over 15 years | Patel et al. | 2020 | multiple |
| Gender Differences in Clinical Outcomes After Percutaneous Coronary Intervention-Analysis of 15,106 Patients from the Cardiac Registry of Pakistan Database | Peerwani et al. | 2023 | south asia |
| Sex-related differences in access to care among patients with ACS | Pelletier et al. | 2014 | high income |
| Age and sex inequalities in the Medication of evidence-based pharmacological therapy following an ACS in Portugal: the EURHOBOP study | Pereira et al. | 2014 | high income |
| Impact of female sex on long-term ACS outcomes | Perl et al. | 2014 | high income |
| Sex differences in Discharge Transfer following MI | Perl et al. | 2018 | high income |
| Outcomes of a routine invasive strategy in elderly patients with non-ST-segment elevation myocardial infarction from 2005 to 2014: results from the PL-ACSregistry | Pi_tek et al. | 2019 | central europe / eastern europe / asia |
| Gender-related disparities in the Treatment and outcomes in patients with non-st-segment elevation myocardial infarction: Results from the Polish Registry of ACS (PL-ACS) in the years 2012-2014 | Pia and cedil and tek et al. | 2020 | central europe / eastern europe / asia |
| Gender differences in short- and long-term Mortality in the Vienna STEMIregistry | Piackova et al. | 2017 | high income |
| Age- and Gender-related Disparities in Primary Percutaneous Coronary Interventions for Acute ST-segment elevation Myocardial Infarction | Pilgrim et al. | 2015 | high income |
| Contemporary differences between men and women with ACS: CIAM multicenter registry | Plaza-Martín et al. | 2019 | high income |
| Pre-hospital Delay and Its Contributing Factors in Patients with ST-Elevation Myocardial Infarction; a Cross sectional Study | Poorhosseini et al. | 2019 | north africa / middle east |
| Persistent sex disparities in clinical outcomes with percutaneous coronary intervention: Insights from 6.6 million PCI procedures in the United States | Potts et al. | 2018 | high income |
| Evaluating Sex Disparities in the Emergency Department Management of Patients With Suspected ACS / Suspected ACS / Chest pain | Preciado et al. | 2021 | high income |
| Clinical Profiles, Outcomes, and Sex Differences of Patients With STEMI: Findings From the NORIN-STEMIRegistry | Qamar et al. | 2023 | south asia |
| Effects of prehospital 12-lead ECG on processes of care and Mortality in ACS: a linked cohort study from the Myocardial Ischaemia National Audit Project | Quinn et al. | 2014 | high income |
| ST-segment elevation myocardial infarction in women with type 2 diabetes | Radomska et al. | 2013 | central europe / eastern europe / asia |

| Title | First author | Publication year | GBD region |
|---|---|---|---|
| Gender differences in the decrease of in-hospital Mortality in patients with MI during the last 20 years in Switzerland | Radovanovic et al. | 2017 | high income |
| Outcomes of thrombus aspiration during primary percutaneous coronary intervention for ST-elevation myocardial infarction | Rajakariar et al. | 2022 | high income |
| Coronary Artery Bypass Grafting Following ACS: Impact of Gender | Ram et al. | 2022 | high income |
| Baseline risk, timing of invasive strategy and guideline compliance in NSTEMI: Nationwide analysis from MINAP | Rashid et al. | 2020 | high income |
| Temporal trends and predictors of Time to Angiography following non-ST-elevation ACS in the USA | Rashid et al. | 2019 | high income |
| Characteristics and outcome among patients who dial for the EMS due to Suspected ACS / Chest pain | Rawshani et al. | 2014 | high income |
| Impact of regionalizing ST-elevation myocardial infarction care on sex differences in reperfusion times and clinical outcomes | Rayner-Hartley et al. | 2021 | high income |
| The pattern of risk-factor profile in Egyptian patients with ACS: phase II of the Egyptian cross-sectional CardioRisk project | Reda et al. | 2019 | north africa / middle east |
| Prevalence of atherosclerosis risk factors in Egyptian patients with ACS: Final data of the nationwide cross-sectional ‘CardioRisk’ project | Reda et al. | 2020 | north africa / middle east |
| Trends in Gender Differences in Cardiac Care and Outcome After MI in Western Sweden: A Report From the Swedish Web System for Enhancement of Evidence-Based Care in Heart Disease Evaluated According to Recommended Therapies (SWEDEHEART) | Redfors et al. | 2015 | high income |
| Primary percutaneous coronary intervention in octogenarians | Ricci et al. | 2016 | multiple |
| Changes in Treatment for NSTEMIin women and the elderly over the past 16_years in a large real-world population | Riehle et al. | 2020 | high income |
| Implementation of the ESC STEMI guidelines in female and elderly patients over a 20-year period in a large German registry | Riehle et al. | 2023 | high income |
| Impact of age, gender and indigenous status on access to Diagnosis Angiography for patients presenting with non-ST segment elevation ACS in Australia | Roe et al. | 2013 | high income |
| Gender-related Mortality trends among diabetic patients with ST-segment elevation myocardial infarction: insights from a nationwide registry 1997-2010 | Roffi et al. | 2013 | high income |
| Understanding a woman's heart: Lessons from 14 177 women with ACS | Roque et al. | 2020 | high income |
| Initial Complications and factors related to prehospital Mortality in MI with ST segment elevation | Rosell-Ortiz et al. | 2015 | high income |
| Sex differences in Mortality after an ACS increase with lower country wealth and higher income inequality | Rossello et al. | 2022 | multiple |
| Impact of Sex and Contact-to-Device Time on Clinical Outcomes in Acute ST-Segment Elevation Myocardial Infarction-Findings From the National Cardiovascular Data Registry | Roswell et al. | 2017 | high income |
| Differences in Presentation, Management and Outcomes in Women and Men Presenting to an Emergency Department With Possible Cardiac Suspected ACS / Chest pain | Ruane et al. | 2017 | high income |
| Sex differences in Treatment and prognosis of ACS with interventional management | Ruiz-Pizarro et al. | 2019 | high income |
| Twelve-Year History of STEM Management in Tehran Heart Center: Concomitant Reduction of In-Hospital Mortality and Hospitalization Length | Saadatagah et al. | 2020 | north africa / middle east |
| Sex Differences in the Management and 5-Year Outcome of Young Patients (<55 Years) with ACS | Sabbag et al. | 2017 | high income |
| Impact of Arterial Access Route on Bleeding Complications in Japanese Patients Undergoing Percutaneous Coronary Intervention- Insight From the PRASFIT Trial | Saito et al. | 2015 | high income |
| Sex bias in admission to tertiary-care centres for MI and cardiogenic shock | Sambola et al. | 2021 | high income |

| Title | First author | Publication year | GBD region |
|---|---|---|---|
| Impact of sex differences and network systems on the in-hospital Mortality of patients with ST-segment elevation MI | Sambola et al. | 2021 | high income |
| Invasive Angiography after Suspected ACS / Chest pain Presentations to Emergency Departments | Sanfilippo et al. | 2020 | high income |
| High-grade atrioventricular block in ACS: Portuguese experience | Santos et al. | 2021 | high income |
| Sex differences in Time to primary percutaneous coronary intervention and outcomes in patients presenting with ST-segment elevation myocardial infarction | Savage et al. | 2022 | high income |
| Gender Differences in Associations Between Intraprocedural Thrombotic Events During Percutaneous Coronary Intervention and Adverse Outcomes | Schoos et al. | 2016 | high income |
| Gender Differences in Patients Admitted to a Certified German Suspected ACS / Chest pain Unit: Results from the German Suspected ACS / Chest pain Unit Registry | Settelmeier et al. | 2020 | high income |
| Gender differences in the revascularization rates and in-hospital outcomes in hospitalizations with ST segment elevation myocardial infarction | Shah et al. | 2020 | high income |
| Cardiogenic Shock Among Patients with Acute ST-Segment Elevation Myocardial Infarction in a Middle Eastern Country: A Single-Center Experience | Shalaby et al. | 2022 | north africa / middle east |
| Epidemiological profile, management and outcomes of patients with ACS: Single centre experience from a tertiary care hospital in North India | Sharma et al. | 2021 | south asia |
| Outcomes and Prognostic Impact of Prophylactic Oral Anticoagulation in Anterior ST_Segment Elevation Myocardial Infarction Patients With Left Ventricular Dysfunction | Shavadia et al. | 2017 | high income |
| Predictors of reperfusion delay in patients with ST elevation myocardial infarction self-transported to the hospital (from the American Heart Association's Mission: Lifeline Program) | Shavelle et al. | 2014 | high income |
| Gender disparities in the presentation, management and outcomes of acute coronary syndrome patients: data from the 2nd Gulf Registry of Acute Coronary Events (Gulf RACE-2) | Shehab et al. | 2013 | north africa / middle east |
| Clinical Presentation, Quality of Care, Risk Factors and Outcomes in Women with Acute ST-Elevation Myocardial Infarction (STEMI): An Observational Report from Six Middle Eastern Countries | Shehab et al. | 2019 | north africa / middle east |
| Age-Related Sex Differences in Clinical Presentation, Management, and Outcomes in ST-Segment-Elevation Myocardial Infarction: Pooled Analysis of 15 532 Patients From 7 Arabian Gulf Registries | Shehab et al. | 2020 | north africa / middle east |
| Gender differences in ACS in Arab Emirati women--implications for clinical management | Shehab et al. | 2013 | north africa / middle east |
| Gender Differences in South Indians with Premature CAD (< 40 Years)-Insights from the PCAD Registry | Shetty et al. | 2021 | south asia |
| New risk factors for early- and late-onset cardiac rupture in ST-elevation myocardial infarction patients after primary percutaneous coronary intervention | Shoji et al. | 2022 | high income |
| Mortality and Heart Failure Hospitalization Among Young Adults With and Without Cardiogenic Shock After MI | Siddiqi et al. | 2023 | high income |
| The Effect of Periprocedural Clinical Factors Related to the Course of STEMI in Men and Women Based on the National Registry of Invasive Cardiology Procedures (ORPKI) between 2014 and 2019 | Sielski et al. | 2021 | central europe / eastern europe / asia |
| [MI in women. Initial characteristics, management and early outcome. The FAST-MI registry] | Simon et al. | 2013 | high income |
| Coronary revascularization for MI in the HIV population | Singh et al. | 2017 | high income |
| Frequency of Angiography and revascularization among men and women with myocardial infarction and their relationship to Mortality at one year: an analysis of the Geisinger myocardial infarction cohort | Skelding et al. | 2013 | high income |
| Mortality of Myocardial Infarction by Sex, Age, and Obstructive CAD Status in the ACTION Registry-GWTG (Acute Coronary Treatment and Intervention Outcomes Network Registry-Get With the Guidelines) | Smilowitz et al. | 2017 | high income |

| Title | First author | Publication year | GBD region |
|---|---|---|---|
| Are there gender differences in acute management and secondary prevention of ACS in Barbados? A cohort study | Sobers et al. | 2019 | latin america / caribbean |
| Prognostic Differences between Men and Women with ACS. Data from a Brazilian Registry | Soeiro et al. | 2018 | latin america / caribbean |
| Association of bleeding, Mortality and sex in ACS: the missing triangle | Solinas et al. | 2015 | high income |
| Traditional Chinese Medicine for MI in Western Medicine Hospitals in China | Spatz et al. | 2018 | southeast asia / east asia / oceania |
| Sex differences in Treatment and outcomes of patients with in-hospital ST-elevation myocardial infarction | Stehli et al. | 2022 | high income |
| Sex Differences in Prehospital Delays in Patients With ST-Segment-Elevation Myocardial Infarction Undergoing Percutaneous Coronary Intervention | Stehli et al. | 2021 | high income |
| Sex Differences in Radial Access for Percutaneous Coronary Intervention in ACS Are Independent of Body Size | Stehli et al. | 2021 | high income |
| Sex Differences Persist in Time to Presentation, Revascularization, and Mortality in Myocardial Infarction Treated With Percutaneous Coronary Intervention | Stehli et al. | 2019 | high income |
| Sex-Based Differences in Revascularization and 30-Day Readmission After ST-Segment-Elevation Myocardial Infarction in the United States | Steitieh et al. | 2021 | high income |
| Treatment Effect of Percutaneous Coronary Intervention in Men Versus Women With ST-Segment-Elevation Myocardial Infarction | Sulaiman et al. | 2021 | high income |
| Factors associated with longer Time to Treatment for patients with Suspected ACS / Suspected ACS / Chest pain: a cohort study | Sullivan et al. | 2014 | high income |
| The sex difference in 6-month Cardiovascular Complications and its explaining variables in MI survivors: Data from CPACS-3 study | Sun et al. | 2020 | southeast asia / east asia / oceania |
| Relations of Sex to Diagnosis and Outcomes in ACS | Sörensen et al. | 2018 | high income |
| Incidence, predictors and prognosis of acute kidney injury in acute ST-segment elevation myocardial infarction patients undergoing emergent Angiography/primary percutaneous coronary intervention | Tan et al. | 2023 | southeast asia / east asia / oceania |
| Evidence-based oral antiplatelet therapy among hospitalized Chinese patients with MI: results from the Chinese MI registry | Tang et al. | 2021 | southeast asia / east asia / oceania |
| The Insight into the Sex Differences in the Patients Diagnosed with ACS Undergoing Percutaneous Coronary Intervention | Tareen et al. | 2022 | south asia |
| Prehospital aspirin administration for ACS (ACS) in the USA: an EMS quality assessment using the NEMSIS 2011 database | Tataris et al. | 2015 | high income |
| Impact of Direct Cardiovascular Laboratory Activation by Emergency Physicians on False-Positive Activation Rates | Tay et al. | 2016 | high income |
| Sex differences in characteristics and outcome in ACS patients in the Netherlands | Ten Haaf et al. | 2019 | high income |
| Sex-related bleeding risk in ACS patients receiving dual antiplatelet therapy with aspirin and a P2Y12 inhibitor | Ten Haaf et al. | 2023 | high income |
| Non-ST-elevation myocardial infarction in the Netherlands: room for improvement! | Ten Have et al. | 2020 | high income |
| Characteristics of and outcomes for elderly patients with MI: differences between females and males | Thang et al. | 2016 | high income |
| Sociodemographic differences in utilization and outcomes for temporary cardiovascular mechanical support in the setting of cardiogenic shock | Thangam et al. | 2021 | high income |
| Unmet needs for emergency care and prevention of prehospital death in MI | Toshima et al. | 2021 | high income |

| Title | First author | Publication year | GBD region |
|---|---|---|---|
| Sex-based differences in clinical practice and outcomes for Japanese patients with MI undergoing primary percutaneous coronary intervention | Toyota et al. | 2013 | high income |
| Age and Sex Differences and Temporal Trends in the Use of Invasive and NonInvasive strategies in Patients Hospitalized With MI | Tran et al. | 2022 | high income |
| Established evidence-based Treatment guidelines help mitigate disparities in quality of emergency care | Trent et al. | 2021 | high income |
| Characteristics and Outcomes of Patients Admitted With Type 2 Myocardial Infarction | Tripathi et al. | 2021 | high income |
| Sex differences in the effectiveness of early coronary computed tomographic Angiography compared with standard emergency department evaluation for acute Suspected ACS / Chest pain: the rule-out myocardial infarction with Computer-Assisted Tomography (ROMICAT)-II Trial | Truong et al. | 2013 | high income |
| Predictors of Technical Failure in Transradial Angiography and Intervention | Tröbs et al. | 2017 | high income |
| Sustained sex-based Treatment differences in ACS care: Insights from the American Heart Association Get With The Guidelines CAD Registry | Udell et al. | 2018 | high income |
| Outcomes of Women and Men With ACS Treated With and Without Percutaneous Coronary Revascularization | Udell et al. | 2017 | high income |
| Primary Percutaneous Coronary Intervention in Elderly Patients With MI_- An Analysis From a Japanese Nationwide Claim-Based Database | Uemura et al. | 2019 | high income |
| Sex Disparities in the Use and Outcomes of Temporary Mechanical Circulatory Support for MI-Cardiogenic Shock | Vallabhajosyula et al. | 2020 | high income |
| Intravascular ultrasound, optical coherence tomography, and fractional flow reserve use in MI | Vallabhajosyula et al. | 2020 | high income |
| Extracorporeal Membrane Oxygenation Use in MI in the United States, 2000 to 2014 | Vallabhajosyula et al. | 2019 | high income |
| Utilization of Palliative Care for Cardiogenic Shock Complicating MI: A 15-Year National Perspective on Trends, Disparities, Predictors, and Outcomes | Vallabhajosyula et al. | 2019 | high income |
| Ten-year trends, predictors and outcomes of mechanical circulatory support in percutaneous coronary intervention for MI with cardiogenic shock | Vallabhajosyula et al. | 2021 | high income |
| Pulmonary artery catheter use in MI-cardiogenic shock | Vallabhajosyula et al. | 2020 | high income |
| Sex and Gender Disparities in the Management and Outcomes of MI-Cardiogenic Shock in Older Adults | Vallabhajosyula et al. | 2020 | high income |
| Sex disparities in acute kidney injury complicating MI with cardiogenic shock | Vallabhajosyula et al. | 2019 | high income |
| Sex Disparities in the Management and Outcomes of Cardiogenic Shock Complicating MI in the Young | Vallabhajosyula et al. | 2020 | high income |
| Variation in the Adoption of Transradial Access for ST-Segment Elevation Myocardial Infarction: Insights From the NCDR CathPCI Registry | Valle et al. | 2017 | high income |
| Disparities in the management of non-ST-segment elevation myocardial infarction in the United States | Varma et al. | 2023 | high income |
| Gender differences in case fatality rates of MI in Serbia | Vasiljevic et al. | 2014 | central europe / eastern europe / asia |
| Sex and age differences and outcomes in ACS | Vasiljevic-Pokrajcic et al. | 2016 | central europe / eastern europe / asia |
| Influence of gender on ischemic times and outcomes after ST-elevation myocardial infarction | Velders et al. | 2013 | high income |

| Title | First author | Publication year | GBD region |
|---|---|---|---|
| Prognostic Implications of High-Degree Atrio-Ventricular Block in Patients with MI in the Contemporary Era | Velásquez-Rodríguez et al. | 2023 | high income |
| High quality process of care increases one-year survival after MI (MI): A cohort study in Italy | Ventura et al. | 2019 | high income |
| Sex disparities in management and outcomes of cardiac arrest complicating MI in the United States | Verghese et al. | 2022 | high income |
| In-Hospital and Long-Term Mortality in 35,173 Chinese Patients Undergoing Coronary Artery Bypass Grafting in Beijing: Impact of Sex, Age, Myocardial Infarction, and Cardiopulmonary Bypass | Wang et al. | 2017 | southeast asia / east asia / oceania |
| Sex-related differences in clinical outcomes and predictive factors in the very elderly patients with ACS undergoing PCI | Wang et al. | 2022 | southeast asia / east asia / oceania |
| Sex Disparity in Characteristics, Management, and In-Hospital Outcomes of Patients with ST-Segment Elevated Myocardial Infarction: Insights from Henan STEMIRegistry | Wang et al. | 2022 | southeast asia / east asia / oceania |
| Trends in length of stay following ACS hospitalisation in New Zealand 2006-2016: ANZACS-QI 32 study | Wang et al. | 2020 | high income |
| Gender differences and bleeding Complications after PCI on first and second generation DES | Wanha et al. | 2017 | central europe / eastern europe / asia |
| Sex-based differences in quality of care and outcomes in a health system using a standardized STEMI protocol | Wei et al. | 2017 | high income |
| Incidence, Characteristics, and Outcomes of Ventricular Fibrillation Complicating MI in Women Admitted Alive in the Hospital | Weizman et al. | 2022 | high income |
| Very long-term outcomes after MI in young men and women: Insights from the FAST-MI program | Weizman et al. | 2023 | high income |
| Sex-Related Differences in Short- and Long-Term Outcome among Young and Middle-Aged Patients for ST-Segment Elevation Myocardial Infarction Underwent Percutaneous Coronary Intervention | Wen-Xia et al. | 2018 | southeast asia / east asia / oceania |
| Impact of Patient- and System-Level Delays on Reperfusion Among Patients With ST-Elevation Myocardial Infarction | Wenner et al. | 2020 | high income |
| Guideline adherence among prehospital emergency nurses when caring for patients with Suspected ACS / Chest pain: a prospective cohort study | Wibring et al. | 2021 | high income |
| Sex differences in quality indicator attainment for myocardial infarction: a nationwide cohort study | Wilkinson et al. | 2019 | high income |
| Gender difference in the use of coronary interventions for patients with ACS: Experience from a major metropolitan hospital in Melbourne, Australia | Worrall-Carter et al. | 2017 | high income |
| Gender Differences in Presentation, Coronary Intervention, and Outcomes of 28,985 ACS Patients in Victoria, Australia | Worrall-Carter et al. | 2016 | high income |
| Impact of comorbidities and gender on the use of coronary interventions in patients with high-risk non-ST-segment elevation ACS | Worrall-Carter et al. | 2016 | high income |
| Gender-stratified analyses of symptoms associated with ACS in telephone triage: a cross-sectional study | Wouters et al. | 2021 | high income |
| Gender-related differences in antiplatelet Treatment patterns and outcome: Insights from the GReekAntiPlatElet Registry | Xanthopoulou et al. | 2017 | high income |
| Sex and Age Differences in Patients With UAPectoris: A Single-Center Retrospective Study | Xu et al. | 2020 | southeast asia / east asia / oceania |
| Likelihood of Treatment in a coronary care unit for a first-Time myocardial infarction in relation to sex, country of birth and socioeconomic position in Sweden | Yang et al. | 2013 | high income |

| Title | First author | Publication year | GBD region |
|---|---|---|---|
| Sex Differences and Temporal Trends in Revascularization and Outcomes of ST-Elevation Myocardial Infarction in Older Adults in the United States | Ye et al. | 2022 | high income |
| Comparisons of Prehospital Delay and Related Factors Between Acute Ischemic Stroke and MI | Yoon et al. | 2022 | multiple |
| Sex-based differences in bleeding and long term adverse Events after percutaneous coronary intervention for MI: Three year results from the HORIZONS-MI trial | Yu et al. | 2014 | high income |
| Trends in sex differences in clinical characteristics, Treatment strategies, and Mortality in patients with ST-elevation myocardial infarction in Poland from 2005 to 2011 | Z and ecki et al. | 2017 | central europe / eastern europe / asia |
| Gender disparity in early death after ST-elevation myocardial infarction | ZHANG et al. | 2013 | southeast asia / east asia / oceania |
| Heterogeneity of the No-Reflow Group After Primary Percutaneous Coronary Intervention Due to ST-Segment Elevation Myocardial Infarction - Are There Sex Differences? | Zachura et al. | 2022 | central europe / eastern europe / asia |
| Gender-related differences in men and women with ST-segment elevation myocardial infarction and incomplete infarct-related artery flow restoration: A multicenter national registry | Zachura et al. | 2018 | central europe / eastern europe / asia |
| Sex differences in the management of ACS in Italy: data from the MANTRA registry | Zagnoni et al. | 2017 | high income |
| Incidence, in-hospital case-fatality rates, and management practices in Puerto Ricans hospitalized with MI | Zevallos et al. | 2013 | latin america / caribbean |
| Gender and Age Differences Associated With Prehospital Delay in Chinese Patients Presenting With ST-Elevation Myocardial Infarction | Zhang et al. | 2016 | southeast asia / east asia / oceania |
| Gender disparity in early death after ST-elevation myocardial infarction | Zhang et al. | 2013 | southeast asia / east asia / oceania |
| Sex Differences in 1-Year Rehospitalization for Heart Failure and Myocardial Infarction After Primary Percutaneous Coronary Intervention | Zheng et al. | 2019 | high income |
| Age-specific gender differences in early Mortality following ST-segment elevation myocardial infarction in China | Zheng et al. | 2015 | southeast asia / east asia / oceania |
| Sex differences in the incidence and outcomes of MI in Spain, 2016–2018: A matched-pair analysis | de Miguel-Yanes et al. | 2021 | high income |
| Cardiac rehabilitation uptake and its determinants in the Netherlands | van Engen-Verheul et al. | 2013 | high income |
| Sex-Based Differences in Unrecognized Myocardial Infarction | van der Ende et al. | 2020 | high income |

**Supplementary Material 6. Outcomes by care phase, region and direction.**

| Phase | CE. Europe, C. Asia | | | | High Income | | | | Latin America, Caribbean | | | |
|---|---|---|---|---|---|---|---|---|---|---|---|---|
| **Direction** | **Favors women** | **Favors men** | **Mixed** | **No difference** | **Favors women** | **Favors men** | **Mixed** | **No difference** | **Favors women** | **Favors men** | **Mixed** | **No difference** |
| **Pre-hospital** | | | | | | | | | | | | |
| Contact health services | 0 | 0 | 0 | 0 | 1 | 0 | 0 | 2 | 0 | 0 | 0 | 0 |
| Time to medical attention | 0 | 4 | 2 | 1 | 0 | 25 | 9 | 11 | 0 | 0 | 0 | 0 |
| Diagnosis | 0 | 0 | 0 | 0 | 0 | 9 | 3 | 2 | 0 | 0 | 0 | 0 |
| Treatment | 0 | 0 | 0 | 0 | 2 | 3 | 2 | 3 | 0 | 0 | 0 | 0 |
| Events | 1 | 0 | 0 | 0 | 4 | 2 | 0 | 4 | 0 | 0 | 0 | 0 |
| Other | 0 | 0 | 0 | 0 | 0 | 2 | 1 | 0 | 0 | 0 | 0 | 0 |
| **Diagnosis** | | | | | | | | | | | | |
| Time | 0 | 1 | 0 | 0 | 0 | 6 | 0 | 6 | 0 | 0 | 0 | 0 |
| Echocardiography | 0 | 0 | 0 | 1 | 0 | 1 | 1 | 0 | 0 | 0 | 0 | 0 |
| ECG | 0 | 0 | 0 | 0 | 0 | 1 | 0 | 2 | 0 | 0 | 0 | 0 |
| Biomarkers | 0 | 0 | 0 | 0 | 0 | 1 | 0 | 1 | 0 | 0 | 0 | 0 |
| Angiography | 0 | 5 | 0 | 1 | 2 | 48 | 5 | 5 | 0 | 2 | 0 | 0 |
| Imaging | 0 | 0 | 0 | 0 | 0 | 1 | 0 | 0 | 0 | 0 | 0 | 0 |
| Other | 0 | 0 | 0 | 0 | 0 | 2 | 0 | 0 | 0 | 0 | 0 | 0 |
| **Treatment** | | | | | | | | | | | | |
| Time | 0 | 3 | 2 | 2 | 4 | 31 | 26 | 27 | 0 | 0 | 0 | 1 |
| Revascularization | 1 | 5 | 4 | 2 | 2 | 71 | 29 | 16 | 0 | 5 | 1 | 1 |
| Reperfusion | 0 | 3 | 2 | 1 | 0 | 24 | 15 | 6 | 0 | 0 | 0 | 0 |
| Antithrombotics | 0 | 2 | 5 | 2 | 1 | 19 | 25 | 6 | 0 | 1 | 4 | 0 |
| Antihypertensives | 0 | 2 | 2 | 1 | 3 | 7 | 9 | 4 | 0 | 1 | 0 | 3 |
| Lipid-lowering drugs | 0 | 1 | 1 | 2 | 1 | 12 | 1 | 2 | 0 | 1 | 0 | 3 |
| Other medications | 0 | 0 | 1 | 0 | 2 | 2 | 0 | 3 | 0 | 0 | 0 | 0 |
| Other | 0 | 1 | 0 | 0 | 1 | 9 | 1 | 2 | 0 | 0 | 0 | 0 |
| **Events** | | | | | | | | | | | | |
| Complications | 0 | 6 | 3 | 1 | 6 | 31 | 26 | 22 | 0 | 0 | 1 | 2 |
| Mortality | 0 | 8 | 2 | 6 | 11 | 51 | 14 | 38 | 0 | 4 | 0 | 3 |
| **Discharge** | | | | | | | | | | | | |
| Antithrombotics | 0 | 1 | 0 | 0 | 2 | 9 | 17 | 9 | 0 | 0 | 0 | 1 |
| Antihypertensives | 0 | 0 | 1 | 0 | 0 | 8 | 16 | 7 | 0 | 0 | 1 | 0 |
| Lipid-lowering drugs | 0 | 2 | 0 | 0 | 1 | 18 | 4 | 11 | 0 | 0 | 0 | 1 |
| Other medications | 0 | 0 | 0 | 1 | 3 | 2 | 2 | 2 | 0 | 0 | 0 | 0 |
| Lifestyle advice | 0 | 0 | 0 | 0 | 0 | 1 | 0 | 0 | 0 | 0 | 0 | 0 |
| Cardiac Rehabilitation referral | 0 | 0 | 0 | 0 | 0 | 9 | 1 | 1 | 0 | 0 | 0 | 0 |
| Other | 0 | 0 | 0 | 0 | 0 | 1 | 0 | 0 | 0 | 0 | 0 | 0 |

| Phase | N. Africa and Middle-East | | | | SE. Asia and Oceania | | | | S. Asia | | | |
|---|---|---|---|---|---|---|---|---|---|---|---|---|
| Direction | Favors women | Favors men | Mixed | No difference | Favors women | Favors men | Mixed | No difference | Favors women | Favors men | Mixed | No difference |
| **Pre-hospital** | | | | | | | | | | | | |
| Contact health services | 0 | 0 | 0 | 0 | 0 | 0 | 0 | 0 | 0 | 0 | 0 | 0 |
| Time to medical attention | 0 | 3 | 0 | 0 | 0 | 10 | 0 | 0 | 0 | 3 | 0 | 0 |
| Diagnosis | 0 | 0 | 0 | 0 | 0 | 0 | 0 | 0 | 0 | 0 | 0 | 0 |
| Treatment | 0 | 1 | 0 | 0 | 0 | 0 | 0 | 0 | 0 | 0 | 0 | 0 |
| Events | 0 | 0 | 0 | 0 | 0 | 0 | 0 | 0 | 0 | 0 | 0 | 0 |
| Other | 0 | 0 | 0 | 0 | 0 | 0 | 0 | 0 | 0 | 0 | 0 | 0 |
| **Diagnosis** | | | | | | | | | | | | |
| Time | 0 | 2 | 0 | 0 | 0 | 1 | 0 | 0 | 0 | 0 | 0 | 0 |
| Echocardiography | 0 | 0 | 0 | 1 | 0 | 2 | 0 | 0 | 0 | 0 | 0 | 1 |
| ECG | 0 | 0 | 0 | 1 | 0 | 0 | 1 | 0 | 0 | 0 | 0 | 0 |
| Biomarkers | 0 | 0 | 0 | 1 | 0 | 1 | 0 | 0 | 0 | 0 | 0 | 0 |
| Angiography | 0 | 3 | 1 | 1 | 0 | 2 | 0 | 1 | 1 | 1 | 2 | 0 |
| Imaging | 0 | 0 | 0 | 1 | 0 | 1 | 0 | 0 | 0 | 0 | 0 | 0 |
| Other | 0 | 0 | 0 | 1 | 0 | 1 | 0 | 0 | 0 | 0 | 0 | 0 |
| **Treatment** | | | | | | | | | | | | |
| Time | 1 | 4 | 2 | 3 | 0 | 6 | 4 | 4 | 0 | 1 | 3 | 3 |
| Revascularization | 0 | 4 | 5 | 0 | 0 | 6 | 8 | 1 | 0 | 4 | 3 | 2 |
| Reperfusion | 1 | 0 | 2 | 3 | 0 | 5 | 0 | 2 | 0 | 0 | 3 | 1 |
| Antithrombotics | 0 | 2 | 8 | 2 | 0 | 7 | 5 | 4 | 0 | 1 | 3 | 2 |
| Antihypertensives | 0 | 0 | 7 | 3 | 0 | 1 | 4 | 9 | 0 | 0 | 2 | 2 |
| Lipid-lowering drugs | 1 | 2 | 0 | 4 | 1 | 3 | 1 | 9 | 1 | 0 | 0 | 3 |
| Other medications | 3 | 0 | 0 | 2 | 1 | 1 | 0 | 1 | 0 | 1 | 0 | 0 |
| Other | 0 | 0 | 0 | 0 | 2 | 1 | 0 | 1 | 0 | 0 | 0 | 1 |
| **Events** | | | | | | | | | | | | |
| Complications | 0 | 7 | 4 | 5 | 1 | 7 | 5 | 5 | 0 | 2 | 2 | 3 |
| Mortality | 1 | 12 | 1 | 6 | 1 | 10 | 0 | 10 | 0 | 5 | 1 | 4 |
| **Discharge** | | | | | | | | | | | | |
| Antithrombotics | 0 | 3 | 1 | 1 | 0 | 3 | 2 | 1 | 1 | 2 | 2 | 2 |
| Antihypertensives | 0 | 0 | 4 | 0 | 0 | 1 | 4 | 1 | 0 | 1 | 1 | 5 |
| Lipid-lowering drugs | 0 | 2 | 1 | 1 | 1 | 2 | 0 | 2 | 1 | 2 | 0 | 4 |
| Other medications | 1 | 1 | 0 | 0 | 0 | 0 | 0 | 0 | 0 | 2 | 0 | 0 |
| Lifestyle advice | 0 | 0 | 0 | 0 | 0 | 1 | 0 | 0 | 0 | 0 | 0 | 0 |
| Cardiac Rehabilitation referral | 0 | 0 | 0 | 0 | 0 | 0 | 0 | 0 | 0 | 0 | 0 | 0 |
| Other | 0 | 0 | 0 | 0 | 0 | 0 | 0 | 0 | 0 | 0 | 0 | 0 |

| Phase | Multiple | | | |
|---|---|---|---|---|
| **Direction** | **Favors women** | **Favors men** | **Mixed** | **No difference** |
| **Pre-hospital** | | | | |
| Contact health services | 0 | 0 | 0 | 0 |
| Time to medical attention | 0 | 3 | 0 | 0 |
| Diagnosis | 0 | 0 | 0 | 0 |
| Treatment | 0 | 0 | 0 | 0 |
| Events | 0 | 0 | 0 | 0 |
| Other | 0 | 0 | 0 | 0 |
| **Diagnosis** | | | | |
| Time | 0 | 0 | 0 | 0 |
| Echocardiography | 0 | 0 | 0 | 0 |
| ECG | 0 | 0 | 0 | 0 |
| Biomarkers | 0 | 0 | 0 | 0 |
| Angiography | 0 | 8 | 1 | 1 |
| Imaging | 0 | 0 | 0 | 0 |
| Other | 0 | 0 | 0 | 0 |
| **Treatment** | | | | |
| Time | 1 | 6 | 5 | 3 |
| Revascularization | 1 | 8 | 6 | 6 |
| Reperfusion | 0 | 5 | 1 | 0 |
| Antithrombotics | 1 | 5 | 2 | 1 |
| Antihypertensives | 0 | 2 | 1 | 0 |
| Lipid-lowering drugs | 1 | 2 | 0 | 1 |
| Other medications | 0 | 0 | 1 | 0 |
| Other | 0 | 3 | 1 | 0 |
| **Events** | | | | |
| Complications | 0 | 4 | 7 | 4 |
| Mortality | 0 | 12 | 4 | 4 |
| **Discharge** | | | | |
| Antithrombotics | 0 | 2 | 2 | 1 |
| Antihypertensives | 0 | 2 | 3 | 1 |
| Lipid-lowering drugs | 0 | 6 | 0 | 1 |
| Other medications | 0 | 0 | 0 | 0 |
| Lifestyle advice | 0 | 0 | 0 | 1 |
| Cardiac Rehabilitation referral | 0 | 0 | 0 | 0 |
| Other | 0 | 0 | 0 | 0 |

## Supplementary Material 7. Results of the sensitivity analysis.

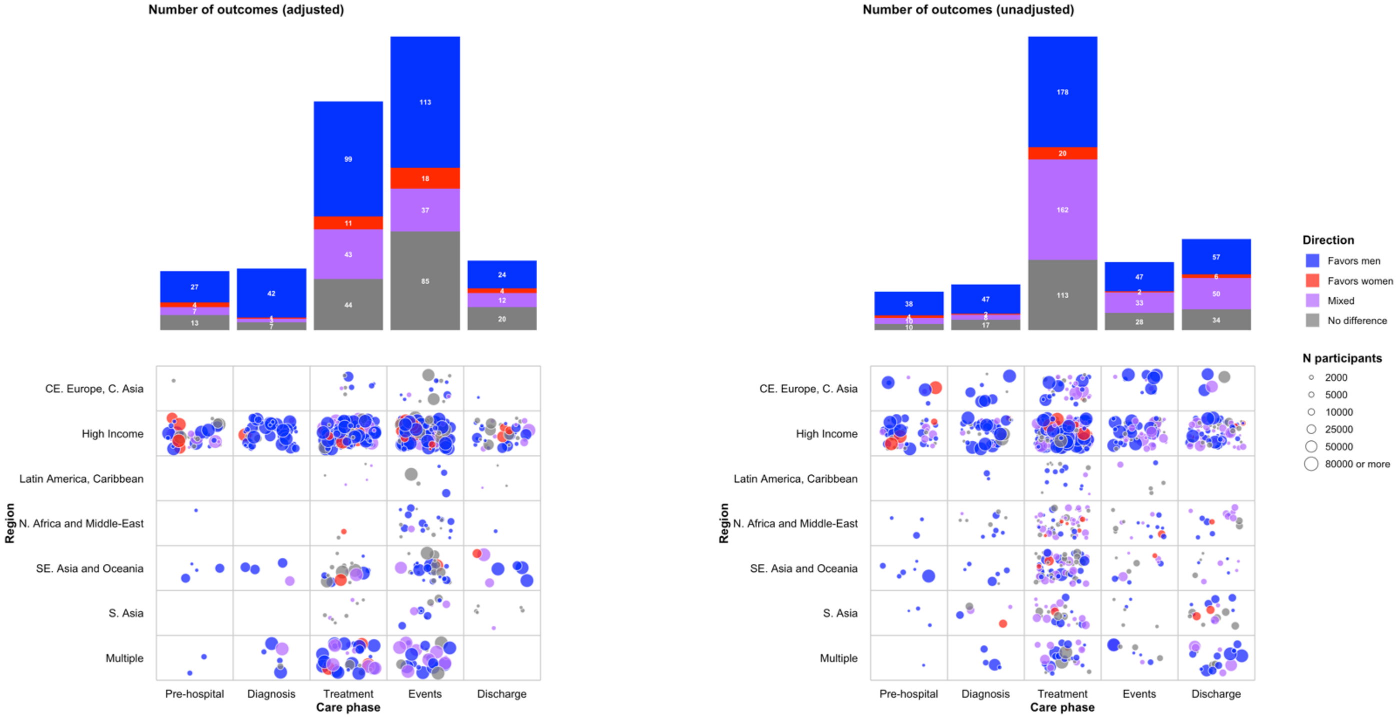

Adjustment by phase
(% by row)

| | Adjusted (N= 614) | Unadjusted (N=866) | Unclear (N=3) |
|---|---|---|---|
| All phases | 41.4 | 58.4 | 0.2 |
| Pre-hospital | 45.1 | 54.9 | 0.0 |
| Diagnosis | 41.4 | 57.8 | 0.8 |
| Treatment | 29.3 | 70.4 | 0.3 |
| Events | 69.7 | 30.3 | 0.0 |
| Discharge | 29.0 | 71.0 | 0.0 |

Direction of sex differences, stratified by adjustment

**All outcomes (% by column)**

| | Overall (N=1483) | Adjusted (N= 614) | Unadjusted (N= 866) | Unclear (N=3) |
|---|---|---|---|---|
| Favors men | 45.5 | 49.7 | 42.4 | 100.0 |
| Favors women | 4.9 | 6.2 | 3.9 | 0.0 |
| Mixed | 24.6 | 16.6 | 30.4 | 0.0 |
| No difference | 25.0 | 27.5 | 23.3 | 0.0 |

**Pre-hospital (% by column)**

| | Overall (N=113) | Adjusted (N=51) | Unadjusted (N=62) |
|---|---|---|---|
| Favors men | 57.5 | 52.9 | 61.3 |
| Favors women | 7.1 | 7.8 | 6.5 |
| Mixed | 15.0 | 13.7 | 16.1 |
| No difference | 20.4 | 25.5 | 16.1 |

**Diagnosis (% by column)**

| | Overall (N=128) | Adjusted (N=53) | Unadjusted (N=74) | Unclear (N=1) |
|---|---|---|---|---|
| Favors men | 70.3 | 79.2 | 63.5 | 100.0 |
| Favors women | 2.3 | 1.9 | 2.7 | 0.0 |
| Mixed | 8.6 | 5.7 | 10.8 | 0.0 |
| No difference | 18.8 | 13.2 | 23.0 | 0.0 |

**Treatment (% by column)**

| | Overall (N=672) | Adjusted (N=197) | Unadjusted (N=473) | Unclear (N=2) |
|---|---|---|---|---|
| Favors men | 41.5 | 50.3 | 37.6 | 100.0 |
| Favors women | 4.6 | 5.6 | 4.2 | 0.0 |
| Mixed | 30.5 | 21.8 | 34.2 | 0.0 |
| No difference | 23.4 | 22.3 | 23.9 | 0.0 |

**Events (% by column)**

| | Overall (N=363) | Adjusted (N=253) | Unadjusted (N=110) |
|---|---|---|---|
| Favors men | 44.1 | 44.7 | 42.7 |
| Favors women | 5.5 | 7.1 | 1.8 |
| Mixed | 19.3 | 14.6 | 30.0 |
| No difference | 31.1 | 33.6 | 25.5 |

**Discharge (% by column)**

| | Overall (N=207) | Adjusted (N=60) | Unadjusted (N=147) |
|---|---|---|---|
| Favors men | 39.1 | 40.0 | 38.8 |
| Favors women | 4.8 | 6.7 | 4.1 |
| Mixed | 30.0 | 20.0 | 34.0 |
| No difference | 26.1 | 33.3 | 23.1 |
